# A monitoring survey and health risk assessment for pesticide residues on Codonopsis Radix in China

**DOI:** 10.1038/s41598-022-11428-w

**Published:** 2022-05-17

**Authors:** Yanping Wang, Jiabin Han, Jinjin Zhang, Xue Li, Ruibin Bai, Fangdi Hu

**Affiliations:** The State Key Laboratory of Applied Organic Chemistry (SKLAOC), School of Pharmacy, Lanzhou University, 199 Dong-gang Road West, Lanzhou, 730000 China

**Keywords:** Health care, Risk factors

## Abstract

In recent years, the safety of Codonopsis Radix (CR) has attracted considerable attention. Pesticide residues is an important index to evaluate the safety of CR. The purpose of this study was to monitor pesticide residues in 164 batches of CR in China and assess dietary risk assessment. Firstly, a combined method of QuEChERS-GC–MS/MS and QuEChERS-LC–MS/MS was established for determination of 155 pesticide residues in CR. Second, 155 Pesticide residues in 3 CR cultivars from Gansu, Shanxi, Hubei, Guizhou and Chongqing were determined by this method. Finally, the risk score of pesticide residues in CR was evaluated, and the dietary health risk was evaluated based on the pesticide residues in CR. The results demonstrated that one or more pesticide residues were detected in 39 batches (23.78%) of 164 batches of CR. Of the 155 pesticide residues, 20 were detected. The most frequently detected pesticide residue was dimethomorph with a detection rate of 5.49%. Risk scores showed that 6 pesticides were at higher risk. Risk assessment based on the hazard quotient/hazard index (HQ/HI) approach revealed that exposure to pesticide residues which detected in CR were far below levels that might pose a health risk.

## Introduction

Statistically, about 3 million tons of pesticides are used globally each yea^1^, 500,000–1 million people are poisoned by pesticides, and 5000–20,000 died from pesticide poisoning every year^2^. In recent years, the use of pesticides has increased substantially to increase the yield of medicinal plants, reduce their storage losses and extend their shelf life^3,4^. At present, there are many studies on the detection of pesticide residues in Chinese herbal medicine, such as *Salvia miltiorrhiza*^5^, *Lycium barbaru*^6^, *Ginseng*^7,8^ and *Panax notoginseng*^9,10^. However, there is little reports on the monitoring and risk assessment of pesticide residues in Codonopsis Radix (CR).

CR is an important tonic in Chinese medicine. Modern pharmacological studies show that it has antioxidant^11,12^, immune enhancement^13,14^, anti-tumour^15,16^, anti-inflammatory^17^ and antiviral^18^ effects. With the rise of “returning to nature” in the world, people have shifted from paying for chemicals to paying for natural botanicals. In the area of disease prevention and treatment, there has been a shift from passive to active prevention and care. CR is an important raw material for both medicine and food use^19^. Its market demand is expanding and so is the area under cultivation. At present, the planting area of CR in China is about 800,000 mu (1 mu = 666.7 m^2^) with a yield of about 70,000 t, of which 40,000 t are used for medicinal diet therapy^19^.

CR is a kind of cultivated medicinal material. To prevent diseases, insects and pests in the growth process, pesticides such as methamidophos, dichlorvos, methomyl, etc., herbicides such as pendimethalin, clethodim, pretilachlor, etc., are widely used in cultivation process of CR^20^. Therefore, it inevitably brings hidden danger to the safety of CR. Risk assessments associated with pesticide residues in fruits and vegetables have been reported^21,22^, hazard quotient(HQ) and hazard index (HI) methods are commonly used to assess the potential risk of pesticide residues in these food products. This assessment method has a certain degree of recognition when assessing the risks posed by pesticides. Performance indicators are generally HQ and HI.

Refer to the list of 33 pesticides that must be tested with Chinese medicinal materials in the Chinese Pharmacopoeia (2020 edition) (Appendix 1), the list of 43 pesticides that must be tested in the National Food Safety Standard (Appendix 2), and the list of 178 pesticide residues that recommended and required be detected in the Group Standard “Pollution-free standard for ginseng” (Appendix 3) (three standards), we screened a total of 155 pesticides as target detection objects including 42 organophosphorus, 13 organochlorine, 11 pyrethroids, and 89 other types. The present study aimed to: (1) Establish a combined method of QuECHERS (quick, easy, cheap, effective, rugged and safe)-GC–MS/MS and QuECHERS-LC–MS/MS for the determination of 155 pesticide residues in 164 batches of CR. (2) Analyze the pesticide residues status of 3 varieties CR which were collected from 5 planting areas in China were systematically analyzed. (3) Risk score for pesticide residues detected in CR. 4) Assess the dietary risks of CR as a drug or food.

## Results

### Method validation

A method for the determination of pesticide residues in CR by LC–MS/MS and GC–MS/MS was established. Total ion chromatogram was shown in Figs. S1, S2, mass spectrum of 155 pesticide residues was seen in Fig. S3. External standards were used to identify and quantify 155 pesticides by comparing with the standard calibration curve with retention time, ion ratio (general value of ± 30%), and peak area as indicators. Linearity was assessed using matrix-matched calibration curves at concentration levels of 1, 10, 20, 40, 80, 160, 240, 320, and 560 ng/mL for GC–MS/MS analysis, 0, 20, 40, 80, 120, and 160 ng/mL for LC–MS/MS analysis. Limit of quantification (LOQ) refers to the minimum amount of a compound that can be quantitatively determined. The LOQ is generally determined by the concentration when the signal-to-noise ratio (S/N) was 10. The LOQ was calculated by injecting low concentrations of 1, 2, 5, 10, 20 and 30 ng/mL, and each treatment was performed in triplicate. As indicated in Tables S1 and S2, all calibration curves present good linearity in the calibration ranges for their coefficient of determination (r^2^) ranging from 0.9067 to 0.998, which were adequate for residue analysis. Recovery experiments were performed at three spik concentrations with three replicates at each level (20, 100, 200 ng/mL). It can be seen from Tables S1 and S2 that the spiking recoveries of 155 pesticides are ranged from 60.11 to 121.40%, which meets the European guidelines SANTE/12682/2019^23^. The multi-standard solution of 50 ng/mL was repeated 6 times on the same day, and the intra-day precision was calculated; the same multi-standard solution was measured continuously for 3 days, and the inter-day precision was calculated. Accuracy based on analysis of peak regions (expressed in RSD) ranged from 1.23 to 11.82% (intra-day) 7.00 to 18.16% (inter-day) (See supplementary materials Tables S1 and S2). The information of retention time, ion pairs, linear equation, correlation coefficient, linear range, LOQ, recovery and precision of 20 detected pesticide residues are shown in Table 1.

**Table 1:** Retention time, detected ion pairs, linear equation, correlation coefficient, linearity range, LOQ, mean recovery, intra-day RSD and inter-day RSD for pesticide residue detected in CR.

Detection method	Pesticide	Mw	Retention time	Product ion* (m/z)	Product ion (m/z)	Linear equation	Correlation coefficient	Linearity range (ng/mL)	LOQ (ng/mL)	Mean recovery (n = 3, %)			Intra-day RSD (n = 3, %)	Inter-day RSD (n = 3, %)
										20 ng/mL	100 ng/mL	200 ng/mL		
GC–MS-MS	Metalaxyl^a^	279.1471	9.12	192.0/160.1	160.0/145.1	Y = 541.44x − 428.60	0.9981	1.00–297.83	1	75.23	78.68	89.65	5.34	13.75
	p,p'-DDT	351.9147	12.88	235.0/165.1	235.0/199.1	Y = 3554.04x − 3712.00	0.9995	2.00–258.62	2	103.40	92.49	96.48	5.59	11.74
	Trifluralin	355.1093	7.15	305.9/264.0	264.0/160.1	Y = 1178.93x − 12522.46	0.9973	2.00–571.35	2	67.43	79.34	89.88	2.26	17.04
	Pyridaben^a^	364.1376	15.33	147.2/117.1	147.2/132.2	Y = 4510.27x − 29644.92	0.9996	2.00–526.63	2	80.51	96.65	98.39	6.67	11.11
	Acephate^a^	183.0119	5.44	136.0/94.0	142.0/96.0	Y = 386.53x − 929.42	0.9975	5.00–604.69	5	87.86	86.68	90.23	6.34	9.87
	Carbofuran^a^	221.1052	7.37	149.1/121.1	164.2/149.1	Y = 1622.50x − 4497.32	0.9986	5.00–483.92	5	74.29	90.38	93.23	10.71	14.66
	O-phenylphenol	170.0732	6.20	169.0/115.1	169.0/141.1	Y = 2613.75x − 6657.70	0.9992	1.00–579.44	1	93.21	98.34	94.56	6.26	15.70
	Diphenylamine	169.0891	6.87	169.0/168.2	168.0/167.2	Y = 4262.48x − 7058.58	0.9994	20.00–507.06	20	101.55	99.60	94.37	6.09	14.11
	Hexachlorobenzene	281.8131	7.64	283.8/213.9	283.8/248.8	Y = 1285.87x − 2377.10	0.9994	2.00–616.68	2	71.47	93.47	85.64	6.88	13.32
	Terbufos^a^	288.0441	7.98	230.9/129.0	230.9/175.0	Y = 2005.75x − 4387.14	0.9996	1.00–483.86	1	101.79	95.44	99.12	1.61	15.92
	Tebuconazole^a^	307.1451	13.06	250.0/125.0	125.0/89.0	Y = 1352.29x − 3380.57	0.9992	2.00–585.84	2	67.50	79.40	86.84	6.70	12.08
	Propargite	350.1552	13.25	135.0/107.1	135.0/77.1	Y = 924.49x + 3511.11	0.9981	1.00–498.75	1	121.40	112.32	104.67	1.63	15.60
LC–MS-MS	Metalaxyl^a^	279.1471	7.55	280.1/220.1	280.1/192.1	y = 422.83x − 1106.73	0.9961	1.00–159.00	1	75.09	98.38	97.73	3.60	15.44
	Clothianidin	249.0087	4.45	250.0/169.0	250.0/132.0	y = 37.263x − 94.2173	0.9942	1.00–157.60	1	115.94	108.03	103.45	8.22	14.65
	Thiamethoxam	291.0193	3.90	292.0/211.2	292.0/132.0	y = 122.285x − 262.599	0.9997	1.00–158.90	1	72.06	98.64	93.57	5.59	16.94
	Carbendazim	191.0695	2.93	192.1/160.1	192.1/132.1	y = 401.125x − 1563.53	0.9987	5.00–156.70	5	68.30	89.75	92.19	7.26	14.77
	Pyridaben^a^	364.1376	13.11	365.1/147.1	365.1/309.1	y = 761.085x − 1543.8	0.9952	1.00–155.40	1	80.02	84.38	89.39	8.62	13.88
	Azoxystrobin	403.1168	9.08	404.0/372.0	404.0/329.0	y = 449.848x − 1954.53	0.9988	2.00–156.00	2	113.06	83.29	87.62	6.32	16.38
	Acephate^a^	183.0119	1.35	184.1/143.0	184.1/125.1	y = 114.371x + 2006.83	0.9067	1.00–167.30	1	117.84	103.58	102.65	8.85	15.46
	Trichlorfon	255.9226	4.20	257.0/109.0	257.0/79.0	y = 73.5238x − 92.7058	0.9945	2.00–157.50	2	70.11	80.50	93.03	3.52	14.10
	Carbofuran^a^	221.1052	6.94	222.1/165.1	222.1/123.0	y = 232.003x − 453.307	0.9961	1.00–155.50	1	77.23	87.40	94.16	7.58	12.19
	Terbufos^a^	288.0441	12.07	289.0/57.2	289.0/103.0	y = 391.675x − 649.147	0.9948	5.00–156.30	5	75.34	80.46	89.47	3.50	14.30
	Phosfolan	255.0153	5.38	256.0/140.0	256.0/168.0	y = 214.288x − 426.581	0.9974	1.00–158.90	1	85.36	90.38	97.38	8.49	11.59
	Zoxamide	335.0247	10.71	336.0/187.1	336.0/159.0	y = 242.655x − 1197.23	0.9963	1.00–153.50	1	70.03	103.20	95.29	7.95	14.96
	Tebuconazole^a^	307.1451	9.44	308.0/70.1	308.0/125.0	y = 496.298x − 15.6863	0.9931	1.00–156.80	1	115.85	102.78	98.37	5.42	16.86
	Dimethomorph	387.1237	8.27	388.1/300.9	388.1/165.0	y = 37.1759x − 256.846	0.9552	2.00–170.50	2	81.51	87.30	89.29	5.78	15.23

^a^Represents that the pesticide residues were tested by both LC–MS/MS and GC–MS/MS.

*Represents quantitative ion pairs.

### Situation of pesticide residues in CR

20 kinds of pesticide residues were detected from CR, including metalaxyl, clothianidin, thiamethoxam, p,p′-DDT, trifluralin, carbendazim, carbofuran, pyridaben, azoxystrobin, acephate, dipterex, o-phenylphenol, diphenylamine, hexachlorobenzene, terbufos, phosfolan, zoxamide, tebuconazole, propargite and dimethomorph. Metalaxyl, pyridaben, acephate, carbofuran, terbufos and tebuconazole could be detected by LC–MS/MS and GC–MS/MS respectively. As the detection limit of the LC–MS/MS method was lower, the data of the above 6 kinds of pesticide residues were determined by LC–MS/MS.

**Figure 1:**
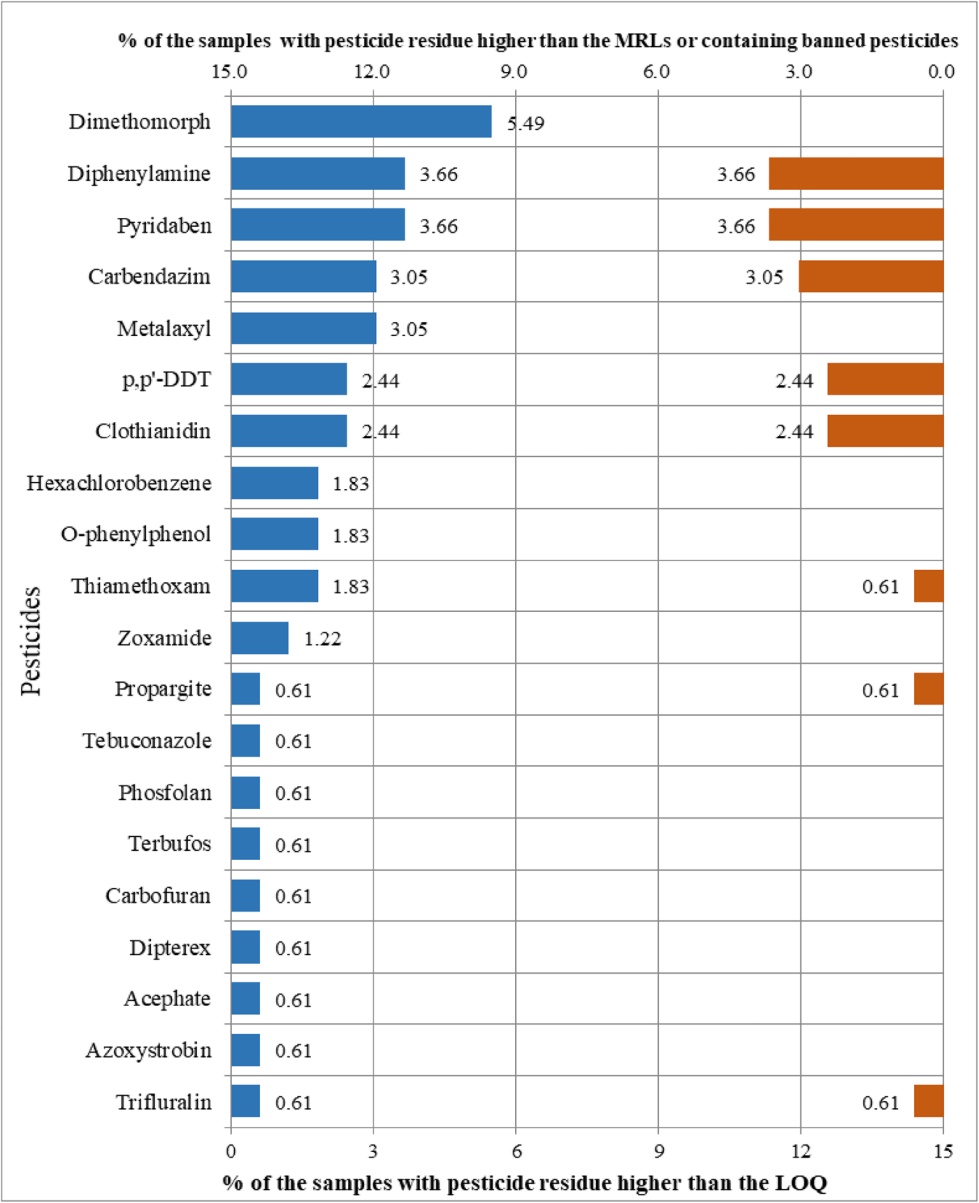
The percentage of pesticide residues detected in CR and the percentage of samples above the maximum detection limit. *Note* the blue bar indicates the proportion of the detected samples, and the results are shown at the bottom (0–15%). The red bar represents the percentage of samples above the maximum limit or containing banned pesticides, which is shown at the top (0–15%).

The usage, toxicity, maximum residue limits (MRLs), acceptable daily intake (ADI) value, acute reference dose (AR*f*D) value, and detection results of 20 kinds of pesticide residues detected in this experiment are shown in Table 2. Among the 20 kinds of pesticide residues detected, dimethomorph had the highest detection rate, with 9 batches of samples detected, and the detection rate was 5.49%. This was followed by pyridine and diphenylamine with a 3.66% detection rate for 6 batches. metalaxyl and carbendazim were detected in 5 batches of CR with a detection rate of 3.05%. Clothianidin, P,P′-DDT were detected in 4 batches of CR with a detection rate of 2.44%. Thiamethoxam, o-phenylphenol and hexachlorobenzene were detected in 3 batches of CR with a detection rate of 1.83%. Zoxamide was detected in 2 batches of CR with a detection rate of 1.22%. Trifluralin, azoxystrobin, acephate, dipterex, carbofuran, terbufos, phosfolan, tebuconazole, and propargite were detected in only 1 batch of CR with a detection rate was 0.61%, as shown in Fig. 1.

**Table 2:** Information and test results of pesticide residues detected.

NO	Names of pesticide residues	purpose	Oral LD_50_ for rat^a^ (mg/kg bw)	Toxicity class^b^	MRL^c^ (mg/kg)	ADI^d^ (mg/kg bw)	AR*f*D^d^ (mg/kg bw)	Positive samples ^f^ (%)	Illegal samples ^g^ (%)	mean^h^(mg/kg)	HR^i^ (mg/kg)
1	Metalaxyl	Bactericide	669	Low	0.05 (T/CATCM001-2018)	0.08	0.5	5 (3.05%)	0 (0.00%)	0.0232	0.0232
2	Clothianidin	Insecticide	> 5000	Low	banned^e^(T/CATCM001-2018)	0.097	0.1	4 (2.44%)	4 (2.44%)	0.0486	0.0486
3	Thiamethoxam	Insecticide	1563	Low	0.02 (T/CATCM001-2018)	0.026	0.5	3 (1.83%)	1 (0.61%)	0.0229	0.0229
4	p,p'-DDT	Insecticide	113(DDT)	Moderate	0.1 (Chinese pharmacopoeia), banned ^e^(T/CATCM001-2018)	0.01	Unnecessary	4 (2.44%)	4 (2.44%)	0.0150	0.0150
5	Trifluralin	Herbicide	> 10,000	Low	banned^e^ (T/CATCM001-2018)	0.015	Unnecessary	1 (0.61%)	1 (0.61%)	0.0537	0.0537
6	Carbendazim	Bactericide	> 5000 ~ 15,000	Low	banned^e^ (T/CATCM001-2018)	0.02	0.02	5 (3.05%)	5 (3.05%)	0.0135	0.0135
7	Pyridaben	Acaricide	1350	Low	banned^e^ (T/CATCM001-2018)	0.2	Unnecessary	6 (3.66%)	6 (3.66%)	0.0114	0.0114
8	Azoxystrobin	Bactericide	> 5000	Low	0.50(T/CATCM001-2018)	0.03	0.1	1 (0.61%)	0 (0.00%)	0.0225	0.0225
9	Acephate*	Insecticide	945	Low	-	0.045	0.1	1 (0.61%)	–	0.0137	0.0137
10	Dipterex*	Insecticide	450–500	Moderate	-	0.00015	0.00015	1 (0.61%)	–	0.0032	0.0032
11	Carbofuran	Insecticide	8–14	High	0.05 (Chinese pharmacopoeia and GB 2763.1–2018)	0.01	0.05	1 (0.61%)	0 (0.00%)	0.0105	0.0105
12	O-phenylphenol*	Bactericide	2700–3000	Low	-	0.4	Unnecessary	3 (1.83%)	–	0.0153	0.0153
13	Diphenylamine	Bactericide	2	Extremely high	0.01(GB 2763.1–2018)	0.075	Unnecessary	6 (3.66%)	6 (3.66%)	0.2529	0.2529
14	Hexachlorobenzene	Bactericide	3500	Low	0.50 (T/CATCM001-2018)	Unnecessary	Unnecessary	3 (1.83%)	0 (0.00%)	0.0128	0.0128
15	Terbufos	Insecticide	2.61	Extremely high	0.02 (Chinese pharmacopoeia)	0.0006	0.002	1 (0.61%)	0 (0.00%)	0.0159	0.0159
16	Phosfolan	Insecticide	0.4	Extremely high	0.03 (Chinese pharmacopoeia)	0.08	0.2	1 (0.61%)	0 (0.00%)	0.0170	0.0170
17	Zoxamide*	Bactericide	> 5000	Low	-	0.5	Unnecessary	2 (1.22%)	–	0.0126	0.0126
18	Tebuconazole	Bactericide	4000	Low	0.50(T/CATCM001-2018)	0.03	0.03	1 (0.61%)	0 (0.00%)	0.0101	0.0101
19	Propargite	Acaricide	2200	Low	banned^e^ (T/CATCM001-2018)	0.007	0.3	1 (0.61%)	1 (0.61%)	0.1629	0.1629
20	Dimethomorph	Bactericide	> 3900	Low	0.05(T/CATCM001-2018)	0.05	0.6	9 (5.49%)	0 (0.00%)	0.0023	0.0023

^a^LD_50_ data were taken from the China Pesticide Information Network (http://www.chinapesticide.gov.cn/).

^b^Toxicity class was according to the Chinese national standard GB 15670-1995.

^c^Maximum residue limits (MRLs) were referred to the Chinese national standard GB 2763.1–2018 for food, the Chinese pharmacopoeia for traditional Chinese medicine (TCM) and Group standard T/CATCM001-2018 for ginseng.

^d^Acceptable daily intakes (ADIs) and acute reference doses (ARfDs) were taken from the EU pesticides database (http://ec.europa.eu/food/plant/pesticides/eu-pesticidesdatabase).

^e^“Banned” indicates that the pesticide is not be detected on traditional Chinese medicine (TCM) in China according to the Chinese national standard GB 2763.1–2018, the Chinese pharmacopoeia and Group standard T/CATCM001-2018.

^f^Samples with pesticide residue higher than the LOQ.

^g^Samples with pesticide residue higher than the MRLs or containing banned pesticides.

^h^“Mean” is the mean residue.

^i^“HR” is the maximum residue.

*Indicates that no maximum limit is specified.

Of the 20 pesticide residues detected, p,p′-DDT, carbofuran, terbufos and phosfolan were required to be detected in the Chinese Pharmacopoeia, with maximum residues of 0.10, 0.05, 0.02 and 0.03 mg/kg, respectively. The content of these 4 pesticide residues detected in this study was lower than the maximum limits, in line with the requirements of the Chinese Pharmacopoeia. Carbendazim, carbofuran and diphenylamine are the pesticide residues with the maximum limits specified in National Food Safety Standard, and the maximum limits are 0.05, 0.05 and 0.01 mg/kg, respectively. The diphenylamine content in 6 batches of samples detected in this project all exceeded the standard. Clothianidin, p,p′-DDT, trifluralin, pyridaben, and propargite are the pesticide residues that are not allowed to be detected in the “Pollution-free standard for ginseng”. In this study, clothianidin was detected in 4 batches of samples, p,p′-DDT in 4 batches of samples, and trifluralin in 1 batch of the sample. 6 batches of samples were detected with pyridaben, 1 batch was detected with propargite, and 16 batches of samples detected the above five kinds of undetectable pesticide residues. The maximum limits of pesticide residues for metalaxyl, thiamethoxam, carbendazim, azoxystrobin, hexachlorobenzene, tebuconazole, and dimethomorph are specified in the “Pollution-free standard for ginseng”. The maximum limits were 0.05, 0.02, 0.10, 0.50, 0.05, 0.50 and 0.05 mg/kg, respectively. The content of thiamethoxam in 1 batch of the sample exceeded the standard, and the rest met the limit requirements. In conclusion, in 164 batches of CR, pesticide residues are in line with the standard of the Chinese Pharmacopoeia, but part of the samples are not in conformity with the National Food Safety Standard and corporate standards “Pollution-free standard for ginseng”. Therefore, we suggest that the control of pesticide residues in CR should be strengthened when it is used as food. According to the catalog and limit of pesticide residues in the three standards, clothianidin, p,p'-DDT, trifluralin, pyridaben, and diphenylamine were not allowed to be detected. 16 batches of 164 samples were considered to be unqualified, and the unqualified rate was 9.76%. Metalaxyl, thiamethoxam, p,p′-DDT, carbendazim, carbofuran, azoxystrobin, diphenylamine, hexachlorobenzene, terbufos, phosfolan, tebuconazole and dimethomorph are the detectable pesticide residues with the specified maximum limits, in this study, thiamethoxam in 1 batch and diphenylamine in 6 batches exceeded the standard, and the unqualified rate was 4.27%, the total unqualified rate was 14.03%.

**Figure 2:**
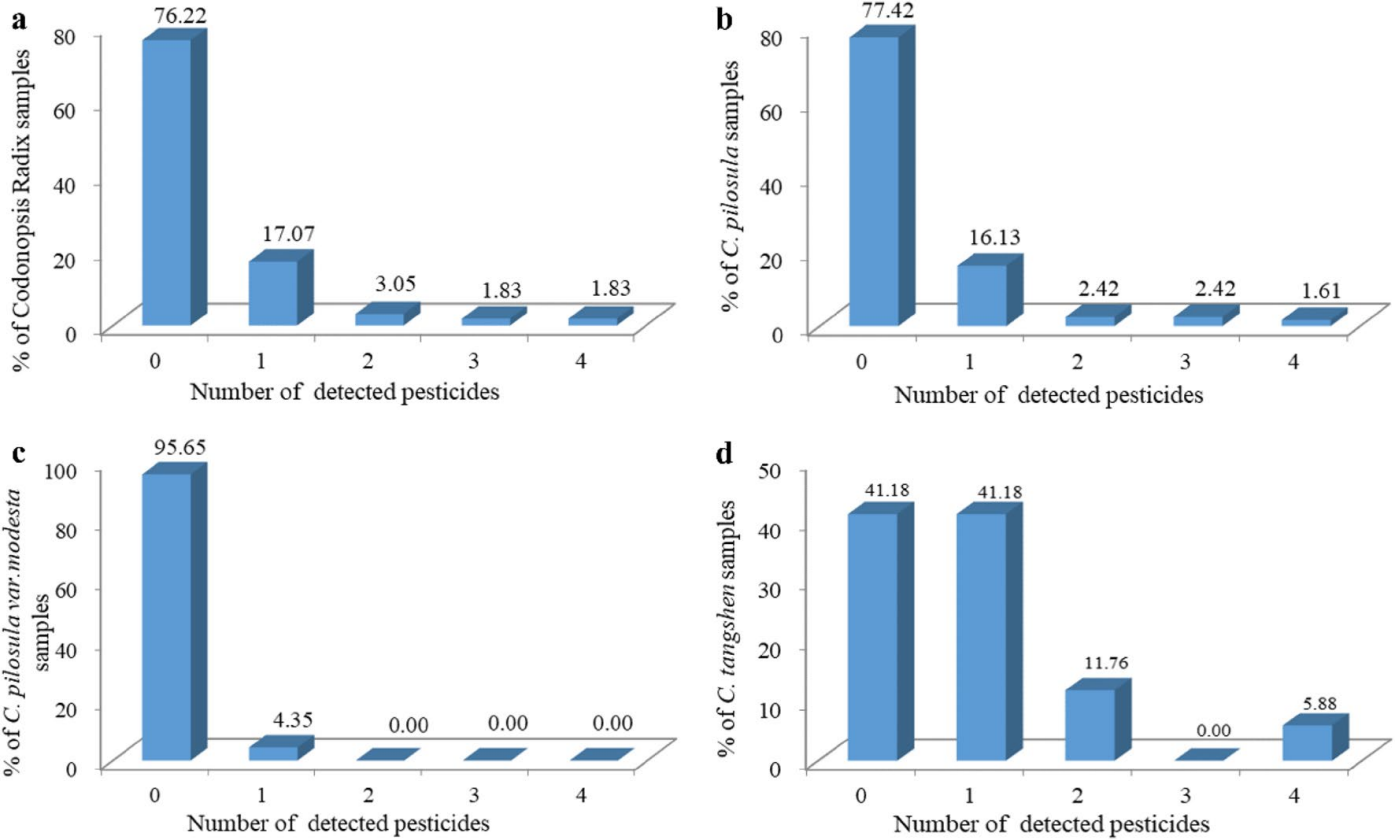
The number and proportion of pesticide residues detected in samples of CR (**a**), *C. pilosula* (**b**), *C. pilosula var. modesta* (**c**), and *C. tangshen* (**d**).

As shown in Fig. 2a, of the 164 CR samples analyzed (124 *Codonopsis Pilosula* (Franch.) Nannf (*C. pilosula*); 23 *Codonopsis Pilosula* Nannf. Var. *modesta* (Nannf.) L. T. Shen (*C. pilosula var. modesta*); 17 *Codonopsis tangshen* Oliv (*C. tangshen*)), 125 samples (76.22%) were residue-free, 39 batches (23.78%) detected one or more pesticide residues. One pesticide residue was detected in 28 batches (17.07%), two were detected in 5 batches (3.05%) and three or more species were detected in 6 batches (3.66%).

In addition, the detection of pesticide residues in different varieties of CR was compared. Among the 124 batches of *C. Pilosula* samples, 96 batches (77.42%) did not detect pesticide residues, 20 batches (16.13%) detected 1 pesticide residues, 3 batches (2.42%) detected 2 pesticide residues, 3 batches (2.42%) detected 3 pesticide residues and 2 batches (1.61%) detected 4 pesticide residues (Fig. 2b). The pesticide residues detected in *C. Pilosula* were acephate, o-phenylphenol, dphenylamine, trifluralin, hexachlorobenzene, carbofuran, metalaxyl, p,p'-DDT, coumaphos, carbendazim, clothianidin, thiacloprid, trichlorfon and dimethomorph, includeing 6 insecticides, 6 bactericides, 1 herbicide and 1 acaricide. Among 23 batches of *C. pilosula var. modesta* samples, 22 batches (95.65%) did not detect pesticide residues, while 1 batch (4.35%) detected 1 kind of pesticide residue (Fig. 2c). The pesticide residues detected in *C. pilosula var. modesta* was clothianidin, it’s belongs to insecticide. Among the 17 *C. tangshen* samples, 7 batches (41.18%) had no pesticide residues detected, 7 batches (41.18%) had 1 pesticide residue detected, 2 batches (11.76%) had 2 pesticide residues detected, and 1 batch (5.88%) detected 4 types of pesticide residues (Fig. 2d). The pesticide residues detected in *C. tangshen* were terbufos, tebuconazole, propargite, phosfolan, malathion and teflubenzuron, includeing 2 insecticides, 3 bactericide 1 acaricide. It can be seen from the above results that the detection rate of pesticide residues in *C. pilosula var. modesta* was low, and only 1 batch of the sample was detected. The detection rate of pesticide residues was higher in *C. tangshen*, and more than 50% of samples were detected with pesticide residues.

Finally, the number of pesticide residues in the 3 varieties of CR was statistically analyzed by t-test. The results showed that there was no significant difference in the number of pesticide residues between *C. pilosula* and *C. pilosula var. modesta*, but there was significant difference between *C. pilosula* (*C. pilosula var. modesta*) and *C. tangshen* (*P* < 0.05).

### Risk assessment

**Figure 3:**
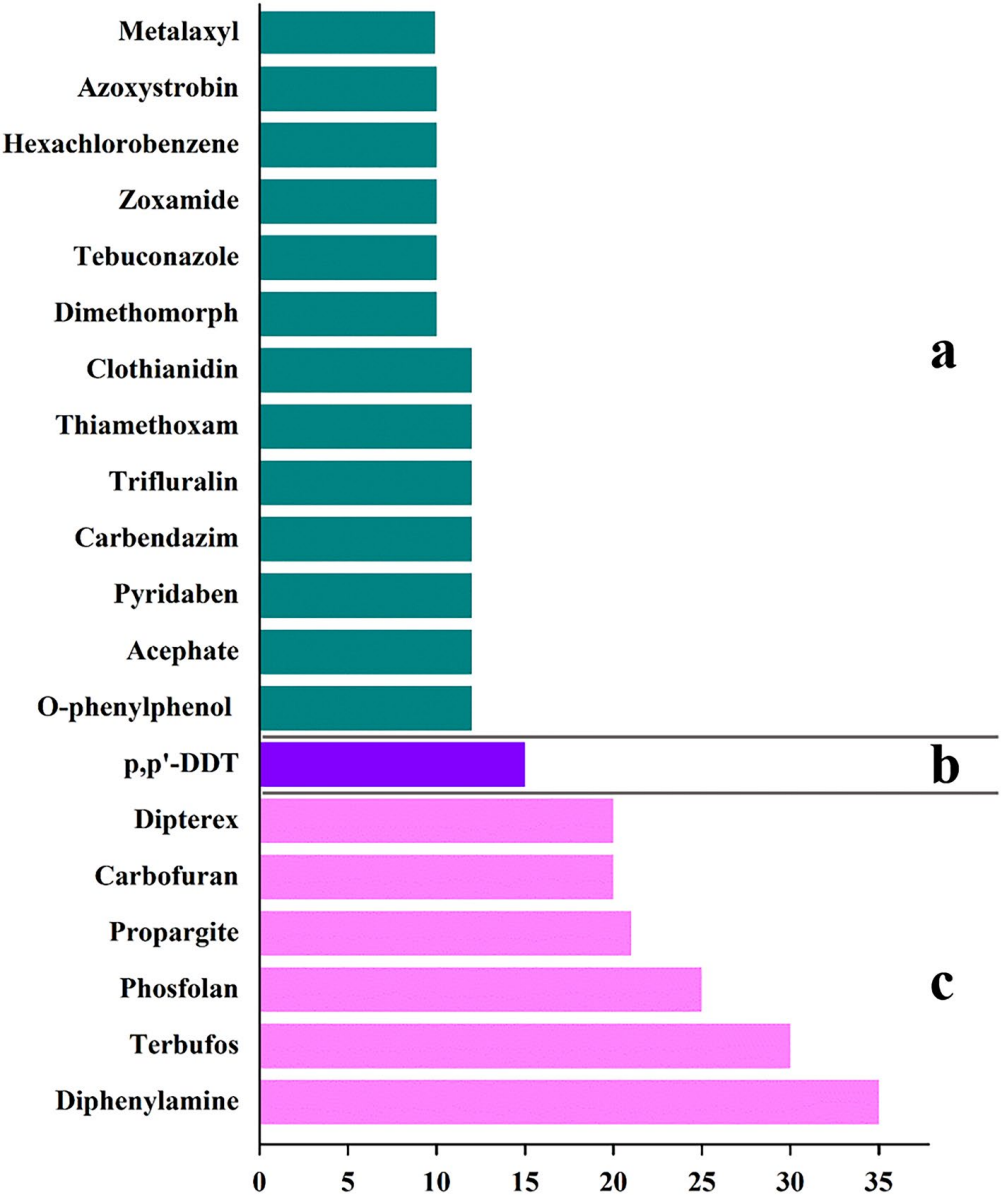
Risk scorings for 20 detected pesticides in CR. (**a**): Low-risk group that scored below 15.0; (**b)**: Medium-risk group that scored between 15.0 and 19.9; (**c**): High-risk group that scored above 20.0.

The risk score of each pesticide residue was calculated according to the criteria in Table 3. Specific as follows: according to the “Chinese Pharmacopoeia”, the daily dosage of CR is 9–30 g. Based on the maximum amount, it can be calculated that the proportion of CR in the diet of Chinese residents is less than 2.5%. Hence, the dietary proportion score (*C*) of CR is determined to be 0. According to the national standard for the rational use of pesticides, each pesticide can only be used up to 3 times in the CR. CR is a perennial herb, and the root development period is more than 180 days. Therefore, the use frequency of each pesticide calculated using formula (1) is less than 2.5%, and the use frequency score (*D*) of the pesticide is determined to be 0. Although there are differences in the consumption of CR among different groups in China, there is no relevant data to determine the existence of high exposure groups. Therefore, the score (*E*) of high exposure groups is determined to be 3. According to the content of each detected pesticide residue, the residue level (F) of 20 kinds of pesticide residues detected can be obtained respectively, among which, the pesticide residues without MRL value are calculated according to the specified value of 0.01 mg/kg. The risk scores of the 20 pesticide residues are shown in Fig. 3. It can be seen that the 20 pesticides can be divided into 3 categories. Category 1 is high-risk pesticides, and there are 6 species, which are diphenylamine, terbufos, phosfolan, propargite, carbofuran, and trichlorfon, with risk scores all ≥ 20. The second category is medium-risk pesticides, and there is only one, namely p,p′-DDT, with a risk score of 15. Category 3 is low-risk pesticides, with a total of 13 species, they are: o-phenylphenol, acephate, pyridaben, carbendazim, trifluralin, thiamethoxam, clothianidin, dimethomorph, tebuconazole, benzamide, hexachlorobenzene, azoxystrobin, metalaxyl, the risk score was less than 15.

**Table 3:** Definition and score of A-F indices for risk scoring.

Item	Item	Definition	Score	Definition	Score	Definition	Score	Definition	Score
A	Toxicity	Low	2	Moderate	3	High	4	Extremely high	5
B	Toxic potency (ADI, mg/kg)	> 1 × 10^–2^	0	1 × 10^–4^ ~ 1 × 10^–2^	1	1 × 10^–6^ ~ 1 × 10^–4^	2	< 1 × 10^–6^	3
C	Diet proportion (%)	< 2.5	0	2.5–20	1	20–50	2	50–100	3
D	Frequency of dosing (%)	< 2.5	0	2.5–20	1	20–50	2	50–100	3
E	Evidence of high exposure groups	No	0	Unlikely	1	Likely	2	Existing	3
F	Residue level (mg/kg)	Nd	1	< 1 MRL	2	≥ 1 MRL	3	≥ 10 MRL	4

*Nd* No evidence of detectable residues.

### Health risk assessment for the detected residues in CR

**Figure 4:**
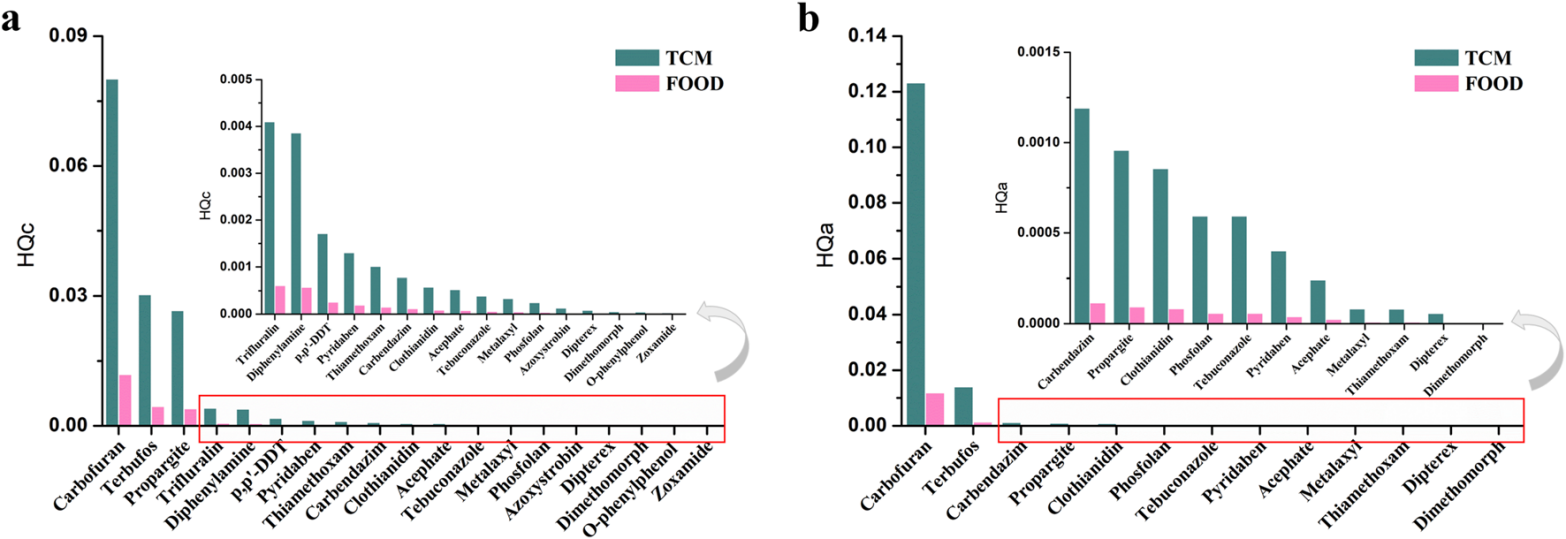
Results of Chronic (**a**) and Acute (**b**) risk assessment of pesticide residues in CR.

Among the 20 pesticide residues detected (metalaxyl, clothianidin, thiamethoxam, p,p′-DDT, trifluralin, carbendazim, carbofuran, pyridaben, azoxystrobin, acephate, dipterex, o-phenylphenol, diphenylamine, terbufos, phosfolan, zoxamide, tebuconazole, propargite, and dimethomorph), 19 had ADI value. HQc values were calculated according to the dosage of CR as medicine and food respectively. The results illustrated that the HQc values of 19 kinds of pesticide residues were less than 1, indicating that the chronic risks caused by 19 kinds of pesticide residues alone were within the tolerance range of humans (Fig. 4a). Furthermore, we calculated the chronic cumulative risk of several kinds of pesticide residues in the samples with the largest number of detected pesticide residues. Three batches of samples, S142, S145 and S163, were found to have the most pesticide residues, and four kinds of pesticide residues were detected respectively. Pyridaben, o-phenylphenol, diphenylamine and hexachlorobenzene were detected in S142, o-phenylphenol, diphenylamine, hexachlorobenzene and p,p′-DDT were detected in S145, and terbufos, phosfolan, tebuconazole and dimethomorph were detected in S163. Among them, pyridaben, diphenylamine, and P,P′-DDT were detected as unqualified samples (S142, S145). Chronic cumulative risk index (HIc) was calculated for the three samples as medicine and food respectively. The results showed that the HIc of S142 as medicine and food were 0.0052 and 0.0008 respectively. The HIc of S145 as medicine and food were 0.0056 and 0.0008 respectively. The HIc of S163 as medicine and food were 0.0310 and 0.0046 respectively. Based on the results, the chronic cumulative risk of CR was within the safe range even if the samples with the most pesticide residues were detected. Finally, we assumed that each batch of CR samples could detect 20 pesticide residues detected in this study, and calculated the HIc values of CR as medicine and food. The results indicated that the HIc was 0.15 when CR was used as medicine and 0.02 when CR was used as food, indicating that the chronic cumulative risk of CR was within the safety range even if the pesticide residues were detected in this study were detected in the same batch of CR.

Among the 20 pesticide residues detected, 13 had AR*f*D values (metalaxyl, clothianidin, thiamethoxam, carbendazim, carbofuran, azoxystrobin, acephate, dipterex, terbufos, phosfolan, tebuconazole, propargite and dimethomorph). HQa values were calculated according to the dosage of CR as medicine and food respectively. The results showed that HQa values of all 13 pesticide residues were < 1, indicating that chronic risks caused by 13 pesticide residues alone were within the tolerance range of humans (Fig. 4b). Further, we calculated the acute cumulative risk of several kinds of pesticide residues in the samples with the largest number of pesticide residues detected (S142, S145 and S163, with 3.2.1 for details). The HIc of S142 as medicine and food is 0.0004 and 0.0000 respectively. The HIc of S145 as medicine and food were 0.0000 and 0.0000 respectively. The HIc of S163 as medicine and food were 0.0148 and 0.0014 respectively. The results indicated that the acute cumulative risk of CR was within the safe range even if the samples with the most pesticide residues were detected. Finally, we assumed that each batch of CR samples could detect 20 pesticide residues detected in this study, and calculated the HIa values of CR as medicine and food. The results showed that when CR was used as medicine or food, the HIa of 20 pesticides were 0.14 and 0.04, which were all less than 1 and indicated that acute cumulative risk should be ignored in the short-term.

## Discussion

In this study, GC–MS/MS and LC–MS/MS methods were used to quantitatively analyze 155 pesticide residues in 164 batches of CR from 3 varieties and 5 major producing areas in China, and a total of 20 pesticide residues were detected. According to the three criteria referenced in this experiment, the unqualified rate of 164 batches of samples was 14.03%. The detection rate of pesticide residues in 3 varieties of CR was compared and analyzed. According to the results, the detection rate of pesticide residues in *C*. *pilosula* var. *modesta* was lower, but that in *C. tangshen* was higher. The producing environment of *C*. *pilosula* var. *modesta* and *C. tangshen* were investigated respectively. The main producing area of *C*. *pilosula* var. *modesta*, Wenxian, belongs to the transition zone from subtropical to warm temperate. *C*. *pilosula* var. *modesta* is most likely to grow in the middle of high mountains, where the climate is mild and cool, and the invasion of pests and grasses is less, the frequency of use of pesticides may be relatively less. *C tangshen* is mainly distributed in subtropical areas, where the air is humid and the precipitation is abundant, so traditional Chinese medicine is easy to be attacked by bacteria, fungi and algae, so the use of fungicides is relatively frequent.

In this study, 20 kinds of pesticide residues detected in CR were risk-scored by referring to the veterinary drug residue risk ranking standard of the British Veterinary Drug Residues Committee. The results showed that there were 6 pesticides with higher risk, namely: diphenylamine, terbution, phosfolan, propargite, carbofuran, and trichlorphon. Among them, trichlorphon is a carcinogen announced by the World Health Organization's International Agency for Research on Cancer. Studies have shown that trichlorfon causes oxidative stress, neurotoxicity, and immune responses in carp^24^^.^ Therefore, we believe that the pesticide residues in CR should not be ignored.

In addition, the study used the Health Risk Assessment Model (2000) developed by the U.S. Environmental Protection Agency to evaluate the chronic and acute health risks of CR as a drug and food. The results demonstrated that when CR was used as medicine or food, the HQc and HQa of 20 pesticide residues were less than 1, indicating that the risks of 20 pesticide residues were acceptable. At the same time, we calculated the cumulative risk index HI of CR as medicine and food. The results exhibited that HIc and HIa values of CR were less than 1 when they were used as medicine or food, indicating that the cumulative risk of CR was within the safety range even if all pesticide residues detected in this study could be detected by the same batch of CR. This experiment proves that when CR is used as a medicine, it will not cause chronic or acute health hazards to the human body if it is within the dose range (9–30 g) prescribed by the Chinese Pharmacopoeia, and it will not cause chronic or acute health hazards to the human body if it is taken for 15 years for adults and 60 days a year. When CR is utilized as food, according to the questionnaire, daily consumption is 20 g^25^. It is employed for 50 years in a person's life and takes 260 days a year. It will not lead to chronic or acute health hazards to humans.

An analysis of the uncertainty in exposure assessment is necessary for this experiment to properly interpret the assessment results. First of all, the consumption of CR as a drug comes from the dosage recommended by the Chinese Pharmacopoeia, not based on the actual dosage, which will lead to higher or lower risk estimates. Therefore, when accurate consumption data is available, a more precise risk assessment should be carried out. Second, The HI values in this study are additive and hypothetical, so the results of the cumulative health risks will be revised as further work on the mechanisms of interaction of these pesticide residues is clarified. Third, the HI value in this study only considered the risk accumulation of multiple pesticide residues in a single sample. However, when CR is used as a medicine, it is often mixed and decocted with other traditional Chinese medicines, which means that the cumulative HI value of pesticide residues in various traditional Chinese medicines may exceed 1.

## Conclusions

China is an important CR producer and commercial region. Therefore, it is necessary to know the actual situation of pesticide residues for CR at the national or regional level and its impact on health of the consumers. In this study, two methods (GC–MS/MS and LC–MS/MS) were established for the determination of 155 pesticide residues in CR. Based on the results, the new methods are suitable for the determination of pesticide residues in the CR. In this study, 40 pesticide residues were determined by two methods, and the results showed that LC–MS/MS had a lower detection limit. Among the pesticide residues detected, 6 repeated pesticide residues were detected by LC–MS/MS method. The monitoring results indicated that 164 CR samples were collected from the three varieties containing one or multiple pesticide residues in 23.78% of samples. Of the monitored samples, 14.03% were still substandard. The results of dietary risk assessment showed that when CR was used as a medicine or food, the HQc, HQa, HIc, and HIa values of the 20 pesticide residues detected were less than 1, indicating that the health risks caused by the detected 20 pesticide residues were acceptable. The 20 pesticides detected were ranked for their risk of ingestion according to a pre-set ranking matrix. The results reflected that 6 pesticides, diphenylamine, terbufos, thiocyclophosphine, propargite, carbofuran, and trichlorfon, had higher risks. Hence, we recommend: (1) The government should strengthen the management of banned and restricted pesticides, and speed up the process of delisting highly toxic pesticides. (2) Strengthen research on the prevention and control of CR disease, pests, weeds, and fungal diseases, and formulate pollution-free standards regarding ginseng medicinal materials as soon as possible. (3) It is recommended that local farmers learn more about physical and biological control methods and avoid the extensive use of pesticides, especially highly toxic pesticides.

## Materials and methods

### Instruments and reagents

7890B-7000D gas chromatograph-tandem mass spectrometry and HP-5MS UI gas chromatography column (30 m × 0.25 mm, 0.25 μm, Agilent, USA); 6460 Triple Quadrupole LC/MS (Agilent, USA); Centrifuge 5810R High-speed centrifuge (Eppendorf, Germany); ME204/02 Electronic Balance (Mettler AG, Switzerland); EVA 50A Nitrogen Blow Instrument (Beijing Pritech Instrument Co., Ltd.); KS501 Shaker (IKA, Germany); Vortex -5 Vortex Mixer (China Qilinbel Instrument Manufacturing Co., Ltd.); MS 205DU electronic balance (accurate to 0.1 mg, Mettler AG, Switzerland).

155 species of pesticide residue standards (See Tables S1 and S2, purity ≥ 95%, Drehrenstorfer, Germany). Acetonitrile, n-hexane (HPLC grade, Merck, Germany); QuEChERS extraction kit 5982-5650 (anhydrous magnesium sulfate, anhydrous sodium acetate, sodium chloride) and purification tube 5982–-5256[*N*-propylethylenediamine (PSA), graphitized carbon black (GCB) and magnesium sulfate] were purchased from Agilent, USA; The test water was Milli-Q ultrapure water. The other reagents have reached analytical purity.

### Sample collection

CR contains 3 varieties, including *C. pilosula*, *C. pilosula var. modesta* and *C. tangshen*. There are 5 main planting areas of CR, including Gansu province, Shanxi province, Hubei province, Guizhou province and Chongqing municipality. Gansu province mainly contains two varieties, *C. pilosula* and *C. pilosula var. modesta*. The Source of CR planted in Shanxi province was *C. pilosula*. The source of CR cultivated in Hubei province, Guizhou province, and Chongqing municipality was *C. tangshen*. After nearly 3 years of field research, 164 batches of samples from core producing areas of the above five provinces or municipalities were collected in this experiment. The CR samples collected in this study can represent the quality status of CR in various producing areas. The area of CR in Gansu province accounts for more than 80% of the whole of China, therefore, there are many CR samples collected in Gansu. Information of 164 batches of samples is shown in Table 4, and the details are given in Table S3. Samples of CR were gathered in the field between 2018 and 2020, and at least 2 kg were collected in each batch. After the fresh samples were washed with water, they were processed following the processing methods in the Chinese Pharmacopoeia, then the medicinal materials of CR (moisture ≤ 16.0%) were obtained. CR was stored in a sealed bag in a refrigerator at -20℃ until analysis, and samples were stored in the CR Research Institute of Lanzhou University.

**Table 4:** Information of CR.

Varieties	Production place	Number of samples
*Codonopsis pilosula*(Franch.)Nannf	Gansu province	113
	Shanxi province	11
*Codonopsis pilosula* Nannf. Var. *modesta*(Nannf.)L. T. Shen	Gansu province	23
*Codonopsis tangshen* Oliv	Hubei province	9
	Guizhou province	4
	Chongqing municipality	4

### Preparation of standard solution

Preparation of pesticide single standard solution: There are 101 reference substances for GC–MS/MS determination and 92 reference substances for LC–MS/MS determination. Precisely weigh 10 mg of each reference substance, dissolve in acetone and dilute to 10 mL to obtain a single standard stock solution of pesticides with a concentration of about 1000 μg/mL, and store at -18 °C.

Preparation of pesticide mixture standard solution: Take a certain volume of the single standard stock solution of 101 pesticides, dilute with n-hexane and dilute to 25 mL, prepare a mixed standard solution of about 8 mg/L, and store at 4 °C. Take a certain volume of a single standard stock solution of 94 pesticides, dilute with acetonitrile and dilute to 25 mL, prepare a mixed standard solution of about 8 mg/L, and store at 4 °C.

Preparation of matrix-matched mixed standard working solution: Firstly, a blank matrix solution was prepared by taking a sample of CR without any pesticide residues. Then, measure 1.0 mL of pesticide mixed standard solution, dilute it to 10 mL with CR blank matrix solution, and prepare a series of matrix-matched mixed standard working solutions.

### Sample preparation

Pre-treatment methods are extremely important for pesticide residue monitoring. The QuEChERS method was introduced in 2003 as a pre-treatment for pesticide monitoring and has been widely used by several governments and scientific standards organizations^26^. At present, QuEChERS method has been broadly applied in analysis of pesticide multi-residues in fruits and vegetables owing to its simplicity, low cost, speed and broad applicability to a wide range of analytes^27,28^. In this study, QuECHERS method was selected for the pretreatment of CR samples. The sample pretreatment of the GC–MS/MS and LC–MS/MS analysis procedures includes the following steps: (1) a portion of 2.0 g of pulverized CR sample was added into a 50 mL centrifuge tube. (2) 10 mL of ultrapure water was added into the tube and the tube was mixed evenly and soaked for 30 min. (3) 10 mL of acetonitrile and two ceramic homogenizers were added, and then the tube was shaken vigorously for 6 min, followed by adding QuEChERS extraction package and kept shaking for the same minutes. (4) the tube was centrifuged at 3900 rpm for 10 min and the supernatant was transferred into a QuEChERS purification tube containing decontaminant. (5) the tube was vortexes for 3 min and centrifuged at 3900 rpm for 10 min, and take the supernatant for use. (6) 2 mL of the supernatant was dried with nitrogen (40 °C) and then dissolved in 1 mL of n-hexane for GC–MS/MS analysis. (7) 2 mL of the supernatant was dried with nitrogen (40 °C) and then dissolved in 1 mL of 60% acetonitrile for LC–MS/MS analysis. Before the analysis, all of them were to be filtered through a nylon filter (0.22 μm).

### GC–MS/MS conditions

Gas chromatography separation was performed on an HP-5MS UI gas chromatography column (30 m × 0.25 mm × 0.25 μm). The gradient heating program was performed as follows: the initial temperature kept at 60 °C for 1 min; raising to 170 °C at 40 °C/min; raising to 310 °C at 10 °C/min and for 3 min. The inlet temperature was set at 280 °C and the carrier gas was high purity nitrogen at a flow rate of 1.2 mL/min. An aliquot of 1 mL of sample extract or standards was injected into the column without shunting.

The following general MS parameters were employed: EI source. The source temperature and gas chromatography-tandem mass spectrometry transmission line temperatures were 250 °C and 280 °C, respectively. The electron energy was 70 eV and the multiple reaction monitoring (MRM) scanning mode was adopted. Agilent Mass Hunter is a working software that was used for data processing and more details were shown in Tables S1 and S2.

### LC–MS/MS conditions

Liquid chromatographic separation was performed on an ACQUITY UPLC BEH C_18_ column (2.1 × 100 mm, 1.8 μm). A mobile phase consisting of eluent A (HPLC grade acetonitrile) and eluent B (0.1% formic acid in water and 5 mmol ammonium acetate) was operated at a flow rate of 0.3 mL/min. The gradient elution was performed as follows: 0–0.5 min, 10% (A); 0.5–12 min, 10–90% (A); 12–13 min, 90–10% (A); 13–15 min, 10% (A). The column temperature was 35 °C and the injection volume was 1 μL.

Mass analysis was performed using an ESI source. The nozzle voltage, dry temperature, dry gas flow rate and capillary voltage were 30 Psi, 500 °C, 900 L/h and 3800 V respectively.

### Risk scoring of pesticide residues

The matrix ranking scheme was developed by the Veterinary Residues Committee of the UK^29,30^. Use toxicity index instead of drug property index. Five other indicators, pesticide toxicity effect (ADI value), dietary ratio (percentage of CR in the total residents' diet, unit: %), frequency of use, high exposure population, and pesticide residue level, adopt the original assignment standards^31^. The assignment criteria for each indicator are shown in Table 3. Toxicity adopts acute oral toxicity and is divided into four categories: Extremely high, high, moderate, and low toxicity according to the oral median lethal dose (LD_50_). The LD_50_ of each pesticide is obtained from the China Pesticide Information Network^32^. The ADI value is obtained from the National Standard Network. The frequency of pesticide use (*FOD*) was calculated using formula (1). The residue risk score (*S*) for each pesticide in the sample is calculated using formula (2). The residue risk score of each pesticide is calculated as the average of the pesticide residue risk score in all samples, the higher the value, the greater the residue risk.

1 $$FOD = T/P \times 100$$

2 $$S = \left( {A + B} \right) \times \left( {C + D + E + F} \right)$$

In formula (1, 2), *P* represents the growth days of CR (the time from transplanting to maturity, unit: d), *T* represents the number of times the pesticide was used during the growth of CR, *A* is the toxicity score, *B* is the score of toxic potency, *C* is the score of the CR diet proportion in total, *D* is the score of the frequency of dosing with a particular pesticide, *E* is the score of the evidence of high exposure groups, and *F* is the score of the detectable pesticide residue level.

### Health risk assessment

According to the health risk assessment model established by US Environmental Protection Agency (2000), the chronic and acute risks caused by pesticide residues in CR were evaluated. The Entropy of chronic hazard (HQc) and the Entropy of acute Hazard (HQa) assess chronic and acute health risks, respectively. When HQ < 1 is considered an acceptable risk, it does not pose a health threat in the long or short term. The higher the HQc or HQa value, the greater the health risk^33^. Further, the cumulative risk of detected pesticides was assessed by HI method. HI is the sum of the HQ of each pesticide^34^. Where HI < 1 is considered an acceptable risk and does not pose a health threat, and HI > 1 is considered an unacceptable risk.

#### Chronic risk assessment

HQc was calculated using Eqs. (3) and (4). In the equation, EDI represents the daily intake of pesticide residues (μg/kg bw) in CR, HQc represents the entropy of chronic hazard.

3 $${\text{EDI}} = \frac{{{\text{EF}} \times {\text{Ed}} \times {\text{IR}} \times {\text{C}}_{1} }}{{{\text{AT}} \times {\text{W}}}}$$

4 $${\text{HQc}} = \frac{{{\text{EDI}} \times {\text{SF}}}}{{{\text{ADI}}}}$$

EF is equal to the number of exposure to toxic substances per year (d). Ed is lifetime exposure time, i.e. lifetime exposure years (y). IR is the daily intake of CR (g). C_1_ is the concentration of single pesticide residue detected in CR (mg/kg), and the average concentration of each pesticide residue is used in this equation. AT refers to the average exposure time of the average pesticide residue. Considering the average life, it is 70 y × 365 d. W is the average weight of adults, which is 60 kg^35^. Thinking that CR is a medicinal material of the same origin as both medicine and food, it can be used as both medicine and food. Therefore, the risk assessment is based on the evaluation of the two situations. Based on the results of the questionnaire, EF = 60 d, Ed = 15 y, and IR 19.5 g were calculated according to the average daily dosage of CR in the Chinese Pharmacopoeia (2020 edition) when CR is used as medicine. When CR is utilized as food, EF = 260 d, Ed = 50 y, IR is 20 g according to the questionnaire^25^.

SF stands for the safety factor. When CR was used as a medicine, according to the safety factors stipulated in Sect. 4 of the Chinese Pharmacopoeia (2020 edition) “Guidelines for establishing limits of harmful residues of traditional Chinese medicine (9302)”, SF means that the daily pesticide residues ingested from traditional Chinese medicine and its products should not exceed 1% of the total daily exposure (including food and drinking water), that is, SF = 100. When CR is used as food, SF = 1. The ADI represents the oral reference dose for pesticide residues and is the dose at which an individual can be continuously exposed to this level for a long period without harm^36^. Chronic exposure risk assessment could not be performed for hexachlorobenzene because there was no available data on AID value. The ADI values of the other 19 pesticide residues detected in this experiment are shown in Table 2.

#### Acute risk assessment

HQa was calculated using formulas (5) and (6). In the equation, EDI represents the daily intake of pesticide residues (μg/kg bw) in CR, HQa represents the entropy of acute hazard.

5 $${\text{EDI}} = \frac{{{\text{EF}} \times {\text{Ed}} \times {\text{IR}} \times {\text{C}}_{2} }}{{{\text{AT}} \times {\text{W}}}}$$

6 $${\text{HQa}} = \frac{{{\text{EDI}} \times {\text{SF}}}}{ARfD}$$

C_2_ is the detected concentration of a single pesticide residue in CR (mg/kg), where the maximum concentration of each pesticide residue is taken. When CR was used as a medicine, IR is 30 g according to the maximum daily dosage of CR in the Chinese Pharmacopoeia (2020 edition). When CR was used as food, IR took 20 g according to the questionnaire results. The meanings and values of other symbols are the same as 5.8.1.

AR*f*D represents the acute reference dose of pesticide residues in medicinal materials^35^. Acute exposure risk assessment could not be performed for p,p’-DDT, trifluralin, pyridaben, o-phenylphenol, diphenylamine, hexachlorobenzene, and zoxamide because the AR*f*D values had been deemed unnecessary for these compounds or because there were no available data on AR*f*D. The AR*f*D values of the other 13 pesticide residues detected in this study are given in Table 2.

#### Cumulative risk assessment

Exposure to two or more chemicals may lead to additives or other interactions, and additive risk usually requires that all components act according to the same mechanism. However, for the quantitative risk assessment of various chemicals, a risk additional hypothesis must be adopted. Therefore, the cumulative health risk caused by pesticide residues in CR was considered to evaluate the total hazard entropy of risks to health caused by multiple pesticide residues. The cumulative health risk represented by HI is calculated using Formula (7). HI < 1 is considered acceptable cumulative risk and does not pose a health threat, while HI > 1 is considered to pose an unacceptable risk^34^.

7 $${\text{HI}} = \mathop \sum \limits_{i = 1}^{n} HQ$$

### Sample collection

The authors declare that they have a license to collect three varieties of Codonopsis Radix (*Codonopsis pilosula* (Franch.) Nannf, *Codonopsis pilosula* Nannf. var. *modesta* (Nannf.) L. T. Shen, *Codonopsis tangshen* Oliv). The authors declare that they comply with the IUCN Policy Statement on Research Involving Species at Risk of Extinction and the Convention on the Trade in Endangered Species of Wild Fauna and Flora.

## Supplementary Information

Supplementary Information 1.

Supplementary Information 2.

Supplementary Information 3.

Supplementary Information 4.

Supplementary Information 5.

## Data Availability

All data generated or analysed during this study are included in this published article and its supplementary information files.
