# Supplementary material for: A monitoring survey and health risk assessment for pesticide residues on Codonopsis Radix in China
Source: Sci Rep. 2022 May 17;12:8133. doi: 10.1038/s41598-022-11428-w (PMC9114365; doi:10.1038/s41598-022-11428-w)
Supplement: Supplementary file 1 — Supplementary Information 1. [file 41598_2022_11428_MOESM1_ESM.docx]

**33 banned pesticides**

| Number | Residue | Quantification Limit（mg/kg） |
| --- | --- | --- |
| 1 | Methamidophos | 0.05 |
| 2 | Parathion-methyl | 0.02 |
| 3 | Parathion | 0.02 |
| 4 | Azodrin | 0.03 |
| 5 | Phosphamidon | 0.05 |
| 6 | Benzex | 0.1 |
| 7 | DDT | 0.1 |
| 8 | Chlordimeform | 0.02 |
| 9 | Nitrofen | 0.05 |
| 10 | Aldrin | 0.05 |
| 11 | Dieldrin | 0.05 |
| 12 | Fenamiphos | 0.02 |
| 13 | Fonofos | 0.02 |
| 14 | Cadusafos | 0.02 |
| 15 | Coumaphos | 0.05 |
| 16 | Sulfotep | 0.02 |
| 17 | Terbufos | 0.02 |
| 18 | Chlorsulfuron | 0.05 |
| 19 | Ethametsulfuron-methyl | 0.05 |
| 20 | Methsulfuron | 0.05 |
| 21 | Thimet | 0.02 |
| 22 | Isofenphos-methyl | 0.02 |
| 23 | Demeton | 0.02 |
| 24 | Carbofuran | 0.05 |
| 25 | Aldicarb | 0.1 |
| 26 | Ethoprophos | 0.02 |
| 27 | Isazofos | 0.01 |
| 28 | Isocarbophos | 0.05 |
| 29 | Endosulfan | 0.05 |
| 30 | Fipronil | 0.02 |
| 31 | Dicofol | 0.2 |
| 32 | Phosfolan | 0.03 |
| 33 | Posfolan-methyl | 0.03 |
