# Supplementary material for: A monitoring survey and health risk assessment for pesticide residues on Codonopsis Radix in China
Source: Sci Rep. 2022 May 17;12:8133. doi: 10.1038/s41598-022-11428-w (PMC9114365; doi:10.1038/s41598-022-11428-w)
Supplement: Supplementary file 2 — Supplementary Information 2. [file 41598_2022_11428_MOESM2_ESM.docx]

**List of 43 pesticide residues to be determined in food**

| Number | Name | Limit Requirements (mg/kg) |
| --- | --- | --- |
| 1 | Paraquat | 0.05 |
| 2 | Pyraclostrobin | 0.03 |
| 3 | Isopyrazam | 0.01 |
| 4 | Propiconazole | 0.01 |
| 5 | Prothioconazole | 0.01 |
| 6 | Profenofos | 0.02 |
| 7 | Glufosinate-ammonium | 0.05 |
| 8 | Tebufenozide | 0.02 |
| 9 | Diflubenzuron | 0.05 |
| 10 | Diquat | 0.05 |
| 11 | Dichlorvos | 0.01 |
| 12 | Fenpropimorph | 0.01 |
| 13 | Carbosulfan | 0.05 |
| 14 | Acetamiprid | 0.01 |
| 15 | Boscalid | 0.02 |
| 16 | Chlorpyrifos | 0.01 |
| 17 | Carbendazim | 0.05 |
| 18 | Spinosad | 0.01 |
| 19 | Famoxadone | 0.01 |
| 20 | Diphenylamine | 0.02 |
| 21 | Diazinon | 0.02 |
| 22 | Flutriafol | 1.0 |
| 23 | Dinotefuran | 0.02 |
| 24 | Flubendiamide | 1.0 |
| 25 | Fluopicolide | 0.01 |
| 26 | Fipronil | 0.02 |
| 27 | Sulfoxaflor | 0.1 |
| 28 | Flusilazole | 0.2 |
| 29 | Cyfluthrin and Beta-cyfluthrin | 0.01 |
| 30 | Emamectin Benzoate | 0.02 |
| 31 | Methamidophos | 0.01 |
| 32 | Phorate | 0.02 |
| 33 | Chlorpyrifos-methyl | 0.01 |
| 34 | Pirimiphos-methyl | 0.01 |
| 35 | Carbaryl | 0.05 |
| 36 | Carbofuran | 0.05 |
| 37 | Quinoxyfen | 0.05 |
| 38 | Dimethoate | 0.05 |
| 39 | Mandipropamid | 5.0 |
| 40 | Trifloxystrobin | 0.05 |
| 41 | Spinetoram | 0.05 |
| 42 | Etoxazole | 0.02 |
| 43 | Fenpyroximate | 0.05 |
