# Supplementary material for: A monitoring survey and health risk assessment for pesticide residues on Codonopsis Radix in China
Source: Sci Rep. 2022 May 17;12:8133. doi: 10.1038/s41598-022-11428-w (PMC9114365; doi:10.1038/s41598-022-11428-w)
Supplement: Supplementary file 3 — Supplementary Information 3. [file 41598_2022_11428_MOESM3_ESM.docx]

**Pollution-free Ginseng Radix et Rhizoma Agricultural Residues Catalogue and Limited Indicators**

1.1 Mandatory pesticide limit indicators

42 pesticides such as Aldrin, Chlorpyrifos, Chlordane, and Quintozene are mandatory inspection items, and their types and maximum residue limits are shown in Table 1.

Table 1 List of pesticide residues to be determined

| Number | Item | Limit Requirements (mg/g) |
| --- | --- | --- |
| 1 | Aldrin | Must not be checked out |
| 2 | Azoxystrobin | 0.50 |
| 3 | BHC(total α-BHC、β-BHC、γ-BHC、δ-BHC） | Must not be checked out |
| 4 | Carbendazim | 0.10 |
| 5 | Chlordane | Must not be checked out |
| 6 | Chlorfenapyr | Must not be checked out |
| 7 | Chlorobenzilate | 0.70 |
| 8 | Chlorpyrifos | 0.50 |
| 9 | Cyazofamid | 0.02 |
| 10 | Cyfluthrin | 0.05 |
| 11 | Cyhalothrin | 0.05 |
| 12 | Cypermethrin | 0.05 |
| 13 | Cyprodinil | 0.80 |
| 14 | total p,p’-DDD、o,p’-DDD、p,p’-DDE、o,p’-DDE、p,p’-DDT、o,p’-DDT | Must not be checked out |
| 15 | Difenoconazole | 0.20 |
| 16 | Dimethomorph | 0.05 |
| 17 | Dinotefuran | 0.05 |
| 18 | Fludioxonil | 0.70 |
| 19 | Flutolanil | 0.05 |
| 20 | Fonofos | Must not be checked out |
| 21 | Heptachlor | Must not be checked out |
| 22 | Hexachlorobenzene | 0.50 |
| 23 | Isofenphos-methyl | 0.02 |
| 24 | Kresoxim-Methyl | 0.10 |
| 25 | Metalaxyl | 0.05 |
| 26 | Methamidophos | Must not be checked out |
| 27 | Methoxyfenozide | 0.05 |
| 28 | Monocrotophos | Must not be checked out |
| 29 | Myclobutanil | 0.05 |
| 30 | Parathion-methyl | Must not be checked out |
| 31 | Pencycuron | 0.05 |
| 32 | Pentachloroaniline(PCA) | 0.02 |
| 33 | Pentachlorothioanisole(PCTA) | 0.01 |
| 34 | Phorate | Must not be checked out |
| 35 | Phoxim | Must not be checked out |
| 36 | Procymidone | 0.20 |
| 37 | Propiconazole | 0.50 |
| 38 | Pyraclostrobin | 0.50 |
| 39 | Quintozene(PCNB) | 0.10 |
| 40 | Tebuconazole | 0.50 |
| 41 | Thiamethoxam | 0.02 |
| 42 | Triflumizole | 0.10 |

1.2 Limit indicators of the pesticide residue for inspecetion

126 pesticides, such as Acephate, Acetamiprid, and Alachlor, are considered items for inspection, and their types and maximum residue limits are shown in Table 2.

Table 2 Pollution-free ginseng radix et rhizoma and decoction pieces——Maximum residue limit of the pesticide residue for inspecetion

| Number | Item | Maximum residue limit (mg/kg) |
| --- | --- | --- |
| 1 | Acephate | Must not be checked out |
| 2 | Acetamiprid | Must not be checked out |
| 3 | Alachlor | Must not be checked out |
| 4 | Avermectin B1a | Must not be checked out |
| 5 | Bentazone | Must not be checked out |
| 6 | β-Benzoepin | Must not be checked out |
| 7 | α-BHC | Must not be checked out |
| 8 | β-BHC | Must not be checked out |
| 9 | γ-BHC | Must not be checked out |
| 10 | δ-BHC | Must not be checked out |
| 11 | Bifenthrin | Must not be checked out |
| 12 | Bromopropylate | Must not be checked out |
| 13 | Buprofezin | Must not be checked out |
| 14 | Butachlor | Must not be checked out |
| 15 | Carbaryl | Must not be checked out |
| 16 | Carbofuran | Must not be checked out |
| 17 | Carbofuran-3-hydroxy | Must not be checked out |
| 18 | Chlorobenzuron | Must not be checked out |
| 19 | cis-Chlordane | Must not be checked out |
| 20 | trans-Chlordane | Must not be checked out |
| 21 | oxy-Chlordane | Must not be checked out |
| 22 | Chlorpyrifos-methyl | Must not be checked out |
| 23 | Chromafenozide | Must not be checked out |
| 24 | Clomeprop | Must not be checked out |
| 25 | Clothianidin | Must not be checked out |
| 26 | Coumatetralyl | Must not be checked out |
| 27 | Cyanophos | Must not be checked out |
| 28 | Cyfluthrin 1 | Must not be checked out |
| 29 | Cyfluthrin 2 | Must not be checked out |
| 30 | Cyfluthrin 3 | Must not be checked out |
| 31 | Cypermethrin | Must not be checked out |
| 32 | o,p'-DDE | Must not be checked out |
| 33 | o,p'-DDT | Must not be checked out |
| 34 | p,p'-DDD | Must not be checked out |
| 35 | p,p'-DDE | Must not be checked out |
| 36 | p,p'-DDT | Must not be checked out |
| 37 | Deltamethrin | Must not be checked out |
| 38 | Diafenthiuron | Must not be checked out |
| 39 | Diazinon | Must not be checked out |
| 40 | Dichlorvos(DDVP) | Must not be checked out |
| 41 | Dicofol | Must not be checked out |
| 42 | Diflufenican | Must not be checked out |
| 43 | Dimethametryn | Must not be checked out |
| 44 | Dimethoate | Must not be checked out |
| 45 | Dimuron | Must not be checked out |
| 46 | Edifenphos | Must not be checked out |
| 47 | Endosulfan sulfate | Must not be checked out |
| 48 | Endrin | 0.02 |
| 49 | EPN | Must not be checked out |
| 50 | Esprocarb | Must not be checked out |
| 51 | Ethion | Must not be checked out |
| 52 | Ethiprole | Must not be checked out |
| 53 | Ethofenprox | Must not be checked out |
| 54 | Ethychlozate | Must not be checked out |
| 55 | Etoxazole | Must not be checked out |
| 56 | Fenbuconazole | Must not be checked out |
| 57 | Fenitrothion(MEP) | Must not be checked out |
| 58 | Fenpropathrin | Must not be checked out |
| 59 | Fenpyroximate | Must not be checked out |
| 60 | Fenthion | Must not be checked out |
| 61 | Fenvalerate 1 | Must not be checked out |
| 62 | Fenvalerate 2 | Must not be checked out |
| 63 | Flonicamid | Must not be checked out |
| 64 | Fluazifop-butyl | Must not be checked out |
| 65 | Fluazinam | Must not be checked out |
| 66 | Flufenoxuron | Must not be checked out |
| 67 | Furametpyr | Must not be checked out |
| 68 | Haloxyfop-methyl | Must not be checked out |
| 69 | cis Heptachlorepoxide | Must not be checked out |
| 70 | Imazosulfuron | Must not be checked out |
| 71 | Imibenconazole | Must not be checked out |
| 72 | Imibenconazole | Must not be checked out |
| 73 | Imibenconazole metabolite | Must not be checked out |
| 74 | Indanofarn | Must not be checked out |
| 75 | Indoxacarb | Must not be checked out |
| 76 | Ioxynil | Must not be checked out |
| 77 | Ipconazole | Must not be checked out |
| 78 | Isocarbofos | Must not be checked out |
| 79 | Isoxathion | Must not be checked out |
| 80 | Ketoconazole | Must not be checked out |
| 81 | Linuron | Must not be checked out |
| 82 | Linuronufenuron | Must not be checked out |
| 83 | Malathion | Must not be checked out |
| 84 | MCPA | Must not be checked out |
| 85 | MCPA-ethyl | Must not be checked out |
| 86 | MCPA-thioethyl | Must not be checked out |
| 87 | Mefenacet | Must not be checked out |
| 88 | Methidathion | Must not be checked out |
| 89 | Methomyl | Must not be checked out |
| 90 | Metolachlor | Must not be checked out |
| 91 | Omethoate | Must not be checked out |
| 92 | Oxaziclomefone | Must not be checked out |
| 93 | Parathion | Must not be checked out |
| 94 | Pendimethalin | 0.01 |
| 95 | Pentoxazone | Must not be checked out |
| 96 | cis-Permethrin | Must not be checked out |
| 97 | trans-Permethrin | Must not be checked out |
| 98 | Phenthoate | Must not be checked out |
| 99 | Phosalone | Must not be checked out |
| 100 | Phthalide | Must not be checked out |
| 101 | Piperonyl butoxide | Must not be checked out |
| 102 | Pirimiphos-methyl | Must not be checked out |
| 103 | Prochloraz | Must not be checked out |
| 104 | Profenofos | Must not be checked out |
| 105 | Prometryn | Must not be checked out |
| 106 | Propanil | Must not be checked out |
| 107 | Propargite | Must not be checked out |
| 108 | Pyridaben | Must not be checked out |
| 109 | Pyrimidifen | Must not be checked out |
| 110 | Pyriminobac-methyl E | Must not be checked out |
| 111 | Pyriminobac-methyl Z | Must not be checked out |
| 112 | Pyroquilone | Must not be checked out |
| 113 | Silafluofen | Must not be checked out |
| 114 | Simeconazole | Must not be checked out |
| 115 | Tebufenozide | Must not be checked out |
| 116 | Tetradifon | Must not be checked out |
| 117 | Thiacloprid | Must not be checked out |
| 118 | Thifensulfuron-methyl | Must not be checked out |
| 119 | Thiobencarb | Must not be checked out |
| 120 | Thiodicarb | Must not be checked out |
| 121 | Thiophanate | Must not be checked out |
| 122 | Tolclofos-methyl | Must not be checked out |
| 123 | Tolfenpyrad | Must not be checked out |
| 124 | Triazophos | Must not be checked out |
| 125 | Triflumizole metabolite | Must not be checked out |
| 126 | Trifluralin | Must not be checked out |
