# Supplementary material for: A monitoring survey and health risk assessment for pesticide residues on Codonopsis Radix in China
Source: Sci Rep. 2022 May 17;12:8133. doi: 10.1038/s41598-022-11428-w (PMC9114365; doi:10.1038/s41598-022-11428-w)
Supplement: Supplementary file 4 — Supplementary Information 4. [file 41598_2022_11428_MOESM4_ESM.docx]

**A monitoring survey and health risk assessment for pesticide residues on Codonopsis Radix in China**

Yanping Wang, Jiabin Han, Jinjin Zhang, Xue Li, Ruibin Bai, Fangdi Hu^*^

School of Pharmacy @ the State Key Laboratory of Applied Organic Chemistry (SKLAOC), Lanzhou University Lanzhou, 730000, China

^*^Corresponding author

Fangdi Hu, Ph.D., School of Pharmacy @ the State Key Laboratory of Applied Organic Chemistry (SKLAOC), Lanzhou University, 199 Dong-gang Road West, Lanzhou 730000, China. Tel.: +86 0931 8911865/8911895; Fax: +86 0931 8915686. E-mail address: hufd@lzu.edu.cn (F. Hu).

**In total: 26 pages**

**Tables: 3**

**Figures: 3**

**Table S1 Retention time, detected ion pairs, Linear equation, Correlation coefficient, Linearity range, LOQ, Mean recovery, Intra-day RSD and Inter-day RSD for pesticide detection by GC-MS-MS**

| Number | Compound | Mw | Retention time(min) | Product ion pair^*^ (*m/z*) | Product ion pair (*m/z*) | Linear | r^2^ | Range（ng/mL） | LOQ (ng/mL) | Mean recovery (n=3, %) | | | | Intra-day RSD (n=3, %) | | Inter-day RSD (n=3, %) |
| --- | --- | --- | --- | --- | --- | --- | --- | --- | --- | --- | --- | --- | --- | --- | --- | --- |
|  |  |  |  |  |  |  |  |  |  | 20 ng/mL | 100 ng/mL | 200 ng/mL | |  |  |  |
| 1 | Methamidophos^a^ | 141.0013 | 4.52 | 141.0/95.0 | 95.0/79.0 | Y=571.50x-4682.34 | 0.9970 | 1.00~493.04 | 1 | 78.23 | 85.48 | 93.42 | | 4.52 | | 9.23 |
| 2 | Dichlorvos^a^ | 219.9459 | 4.529 | 184.9/93.0 | 108.9/79.0 | Y=590.83x-772.31 | 0.9995 | 2.00~383.73 | 2 | 75.34 | 88.36 | 78.65 | | 7.32 | | 10.21 |
| 3 | Acephate^a^ | 183.0119 | 5.44 | 136.0/94.0 | 142.0/96.0 | Y=386.53x-929.42 | 0.9975 | 5.00~604.69 | 5 | 87.86 | 86.68 | 90.23 | | 6.34 | | 9.87 |
| 4 | [O-Phenylphenol](file:///D:\AN\Dict\8.9.4.0\resultui\html\index.html#/javascript:;) | 170.0732 | 6.193 | 169.0/115.1 | 169.0/141.1 | Y=2613.75x-6657.70 | 0.9992 | 1.00~579.44 | 1 | 93.21 | 98.34 | 94.56 | | 6.26 | | 15.70 |
| 5 | Molinate | 187.1031 | 6.263 | 126.2/55.1 | 169.0/141.1 | Y=2013.78x+1866.04 | 0.9991 | 2.00~518.07 | 2 | 103.25 | 94.50 | 92.39 | | 4.48 | | 14.90 |
| 6 | Omethoate^a^ | 213.0225 | 6.431 | 155.9/110.0 | 109.9/79.0 | Y=1076.91x-12353.08 | 0.9964 | 5.00~504.02 | 5 | 113.98 | 99.23 | 103.48 | | 5.56 | | 15.84 |
| 7 | Tecnazene | 258.8761 | 6.796 | 258.9/201.0 | 214.9/9.0 | Y=392.43x-788.81 | 0.9981 | 10.00~245.41 | 10 | 102.35 | 101.45 | 105.49 | | 2.76 | | 13.71 |
| 8 | Diphenylamine | 169.0891 | 6.87 | 169.0/168.2 | 168.0/167.2 | Y=4262.48x-7058.58 | 0.9994 | 20.00~507.06 | 20 | 101.55 | 99.60 | 94.37 | | 6.09 | | 14.11 |
| 9 | Ethoprophos^a^ | 242.0564 | 6.874 | 157.9/97.0 | 138.9/97.0 | Y=1085.59x-612.30 | 0.9991 | 20.00~257.89 | 20 | 97.34 | 91.40 | 97.45 | | 7.20 | | 15.15 |
| 10 | [Chlorpropham](file:///D:\AN\Dict\8.9.4.0\resultui\html\index.html#/javascript:;) | 213.0557 | 7.036 | 127.0/65.1 | 171.0/127.1 | Y=1129.83x-1402.61 | 0.9997 | 5.00~462.27 | 5 | 112.09 | 104.60 | 109.35 | | 2.13 | | 8.17 |
| 11 | Chlordimeform | 196.0767 | 7.202 | 117.0/89.0 | 117.0/90.0 | Y=577.17x-910.11 | 0.9994 | 1.00~524.99 | 1 | 88.65 | 93.45 | 97.34 | | 4.14 | | 17.68 |
| 12 | Bifenazate^a^ | 300.1474 | 7.087 | 184.0/141.1 | 184.0/169.2 | Y=86.58x-613.34 | 0.9986 | 1.00~505.02 | 1 | 83.44 | 88.56 | 89.46 | | 8.59 | | 11.83 |
| 13 | [Trifluralin](file:///D:\AN\Dict\8.9.4.0\resultui\html\index.html#/javascript:;) | 355.1093 | 7.145 | 305.9/264.0 | 264.0/160.1 | Y=1178.93x-12522.46 | 0.9973 | 2.00~571.35 | 2 | 67.43 | 79.34 | 89.88 | | 2.26 | | 17.04 |
| 14 | Monocrotophos^a^ | 223.0610 | 7.283 | 127.1/109.0 | 127.1/95.0 | Y=1433.37x-10810.85 | 0.9974 | 1.00~492.70 | 1 | 78.89 | 94.39 | 87.45 | | 6.65 | | 16.65 |
| 15 | Sulfotep^a^ | 322.0227 | 7.204 | 201.8/145.9 | 321.8/145.8 | Y=791.95x+6894.89 | 0.9941 | 2.00~489.81 | 2 | 70.30 | 84.56 | 86.45 | | 3.00 | | 13.20 |
| 16 | Phorate^a^ | 260.0128 | 7.366 | 121.0/65.0 | 121.0/47.0 | Y=812.07x-823.37 | 0.9996 | 5.00~259.84 | 5 | 72.98 | 90.23 | 94.58 | | 5.18 | | 11.87 |
| 17 | Cadusafos^a^ | 270.0877 | 7.29 | 158.8/97.0 | 126.9/98.9 | Y=2323.23x-1407.10 | 0.9989 | 1.00~243.24 | 1 | 70.79 | 86.78 | 91.25 | | 1.73 | | 9.57 |
| 18 | Hexachlorobenzene | 281.8131 | 7.64 | 283.8/213.9 | 283.8/248.8 | Y=1285.87x-2377.10 | 0.9994 | 2.00~616.68 | 2 | 71.47 | 93.47 | 85.64 | | 6.88 | | 13.32 |
| 19 | [Dicloran](file:///D:\AN\Dict\8.9.4.0\resultui\html\index.html#/javascript:;) | 205.9650 | 7.683 | 160.1/124.1 | 124.1/73.0 | Y=334.08x-1199.02 | 0.9998 | 1.00~570.05 | 1 | 82.29 | 95.67 | 99.24 | | 3.31 | | 14.75 |
| 20 | Dimethoate^a^ | 228.9996 | 7.71 | 86.9/46.0 | 92.0/63.0 | Y=733.98x-2444.94 | 0.9983 | 20.00~491.43 | 20 | 76.59 | 88.57 | 94.57 | | 7.24 | | 10.28 |
| 21 | Carbofuran^a^ | 221.1052 | 7.365 | 149.1/121.1 | 164.2/149.1 | Y=1622.50x-4497.32 | 0.9986 | 5.00~483.92 | 5 | 74.29 | 10.71 | | 14.66 | |  |  |
| 22 | Dimethipin | 2121 | 7.798 | 124.0/76.0 | 118.0/58.1 | Y=94.22x-176.16 | 0.9987 | 30.00~495.43 | 30 | 84.08 | 89.56 | 90.76 | | 6.85 | | 14.57 |
| 23 | Terbufos^a^ | 288.0441 | 7.975 | 230.9/129.0 | 230.9/175.0 | Y=2005.75x-4387.14 | 0.9996 | 1.00~483.86 | 1 | 101.79 | 95.44 | 99.12 | | 1.61 | | 15.92 |
| 24 | Quintozene | 292.8372 | 8.085 | 248.8/213.8 | 295.0/236.8 | Y=468.22x-3017.86 | 0.9993 | 20.00~613.70 | 20 | 82.68 | 94.57 | 98.73 | | 6.42 | | 15.92 |
| 25 | Fonofos^a^ | 246.0302 | 7.819 | 108.9/80.9 | 149.0/77.0 | Y=951.80x+1325.86 | 0.9991 | 10.00~484.79 | 10 | 91.10 | 94.66 | 98.34 | | 4.64 | | 13.53 |
| 26 | Diazinon^a^ | 304.1010 | 7.708 | 137.1/84.0 | 137.1/54.0 | Y=587.58x-937.49 | 0.9995 | 10.00~502.22 | 10 | 105.68 | 104.58 | 103.21 | | 5.72 | | 16.21 |
| 27 | δ-HCH | 287.8601 | 8.385 | 181.0/145.0 | 181.0/109.0 | Y=581.89x-846.47 | 0.9992 | 20.00~288.57 | 20 | 78.16 | 89.56 | 94.58 | | 2.92 | | 11.24 |
| 28 | Chlorothalonil | 263.8816 | 8.481 | 263.8/168.0 | 263.8/229.0 | Y=847.10x-5274.45 | 0.9979 | 20.00~494.15 | 20 | 78.29 | 90.46 | 98.26 | | 6.25 | | 16.13 |
| 29 | Pirimicarb^a^ | 238.1430 | 8.594 | 238.0/166.2 | 166.0/55.1 | Y=1694.73x-2575.40 | 0.9992 | 20.00~488.87 | 20 | 94.67 | 99.56 | 103.43 | | 7.36 | | 15.74 |
| 30 | Phosphamidon^a^ | 299.0689 | 8.784 | 127.0/109.0 | 127.0/95.0 | Y=901.38x-2822.12 | 0.9988 | 2.00~523.12 | 1 | 82.12 | 89.45 | 88.58 | | 2.29 | | 12.29 |
| 31 | [Vinclozolin](file:///D:\AN\Dict\8.9.4.0\resultui\html\index.html#/javascript:;) | 284.9959 | 8.956 | 187.0/124.0 | 197.9/145.0 | Y=510.12x-743.17 | 0.9991 | 2.00~531.68 | 2 | 75.34 | 79.46 | 85.76 | | 4.30 | | 10.96 |
| 32 | Parathion-methyl | 263.0017 | 9.131 | 109.0/79.0 | 262.9/109.0 | Y=404.17x-1704.24 | 0.9972 | 1.00~499.66 | 1 | 83.23 | 94.21 | 91.20 | | 8.75 | | 8.66 |
| 33 | C[hlorpyrifos-methyl](file:///D:\AN\Dict\8.9.4.0\resultui\html\index.html#/javascript:;) | 320.8950 | 9.296 | 285.9/92.9 | 287.9/92.9 | Y=1055.63x-2907.87 | 0.9996 | 10.00~501.98 | 10 | 106.27 | 103.40 | 102.74 | | 2.42 | | 12.41 |
| 34 | [Tolclofos-methyl](file:///D:\AN\Dict\8.9.4.0\resultui\html\index.html#/javascript:;) | 299.9544 | 9.071 | 265.0/250.0 | 265.0/93.0 | Y=2452.50x-2715.65 | 0.9995 | 10.00~459.51 | 10 | 69.76 | 85.79 | 94.59 | | 6.81 | | 13.84 |
| 35 | Heptachlor | 369.8211 | 8.8 | 271.7/236.9 | 273.7/238.9 | Y=1478.97x-1697.65 | 0.9994 | 2.00~484.44 | 2 | 79.03 | 89.59 | 94.78 | | 3.16 | | 9.37 |
| 36 | Metalaxyl^a^ | 279.1471 | 9.115 | 192.0/160.1 | 160.0/145.1 | Y=541.44x-428.60 | 0.9981 | 1.00~297.83 | 1 | 75.23 | 78.68 | 89.65 | | 5.34 | | 13.75 |
| 37 | [Fenitrothion](file:///D:\AN\Dict\8.9.4.0\resultui\html\index.html#/javascript:;) | 277.0174 | 9.15 | 125.1/47.0 | 277.0/260.1 | Y=1071.87x-4437.61 | 0.9988 | 20.00~485.53 | 20 | 86.74 | 94.67 | 97.43 | | 1.89 | | 13.66 |
| 38 | [Pirimiphos-methyl](file:///D:\AN\Dict\8.9.4.0\resultui\html\index.html#/javascript:;) | 305.0963 | 9.815 | 290.0/125.0 | 304.9/180.0 | Y=1001.74x-1309.86 | 0.9994 | 20.00~501.57 | 20 | 105.88 | 104.53 | 102.89 | | 7.04 | | 15.01 |
| 39 | [Malathion](file:///D:\AN\Dict\8.9.4.0\resultui\html\index.html#/javascript:;) | 330.0361 | 9.597 | 172.9/99.0 | 126.9/99.0 | Y=1827.43x-4698.94 | 0.9990 | 10.00~549.65 | 10 | 79.34 | 88.98 | 90.64 | | 3.47 | | 9.01 |
| 40 | Fenthion^a^ | 278.0200 | 9.673 | 278.0/109.0 | 124.9/47.0 | Y=1744.21x-1145.77 | 0.9998 | 5.00~488.76 | 5 | 72.17 | 87.01 | 94.50 | | 7.40 | | 12.62 |
| 41 | [Aldrin](file:///D:\AN\Dict\8.9.4.0\resultui\html\index.html#/javascript:;) | 361.8757 | 9.531 | 262.9/192.9 | 262.9/190.0 | Y=466.54x+172.13 | 0.9988 | 1.00~242.17 | 1 | 92.30 | 89.54 | 96.47 | | 8.34 | | 15.30 |
| 42 | Chlorpyrifos^a^ | 348.9263 | 9.778 | 314.0/258.0 | 314.0/286.0 | Y=900.19x-255.18 | 0.9992 | 20.00~310.12 | 20 | 77.76 | 88.58 | 89.20 | | 7.01 | | 10.33 |
| 43 | [Parathion](file:///D:\AN\Dict\8.9.4.0\resultui\html\index.html#/javascript:;) | 291.0330 | 9.788 | 290.9/109.0 | 138.9/109.0 | Y=816.11x-7571.47 | 0.9974 | 1.00~492.22 | 1 | 109.74 | 104.38 | 102.21 | | 1.77 | | 11.64 |
| 44 | Triadimefon^a^ | 293.0931 | 9.737 | 208.0/181.0 | 208.0/111.0 | Y=842.22x-1023.33 | 0.9996 | 1.00~593.86 | 1 | 91.31 | 97.58 | 90.30 | | 5.72 | | 10.51 |
| 45 | Isocarbofos^a^ | 289.0538 | 10.107 | 135.9/108.0 | 120.0/92.0 | Y=1983.13x-4271.54 | 0.9994 | 5.00~486.30 | 5 | 77.64 | 85.69 | 85.90 | | 1.91 | | 12.06 |
| 46 | Cyprodinil | 225.1266 | 10.142 | 225.2/224.3 | 224.2/208.2 | Y=3644.78x-4026.09 | 0.9995 | 10.00~548.77 | 10 | 95.59 | 96.78 | 98.38 | | 11.82 | | 11.71 |
| 47 | [Isofenphos-methyl](file:///D:\AN\Dict\8.9.4.0\resultui\html\index.html#/javascript:;) | 331.1007 | 10.604 | 199.0/121.0 | 241.0/121.0 | Y=2963.34x-2612.57 | 0.9989 | 1.00~248.18 | 1 | 94.15 | 99.60 | 104.58 | | 7.75 | | 13.56 |
| 48 | Pendimethalin^a^ | 281.1376 | 10.495 | 251.8/162.2 | 251.8/161.1 | Y=542.97x-5756.66 | 0.9978 | 2.00~561.89 | 2 | 92.65 | 90.48 | 98.62 | | 6.40 | | 10.69 |
| 49 | [Probenazole](file:///D:\AN\Dict\8.9.4.0\resultui\html\index.html#/javascript:;) | 201.0361 | 10.684 | 201.0/174.0 | 128.9/102.0 | Y=2784.85x-10789.87 | 0.9948 | 1.00~248.65 | 1 | 82.88 | 89.78 | 95.89 | | 3.87 | | 14.09 |
| 50 | Toluene fluoride Sulfonamide | 345.9780 | 10.518 | 136.9/91.1 | 237.9/137.0 | Y=1663.56x-6867.10 | 0.9988 | 20.00~497.27 | 20 | 100.64 | 92.78 | 95.48 | | 8.96 | | 10.72 |
| 51 | [Fipronil](file:///D:\AN\Dict\8.9.4.0\resultui\html\index.html#/javascript:;) | 435.9387 | 10.705 | 350.7/254.9 | 254.8/228.0 | Y=81.49x+103.17 | 0.9994 | 2.00~320.30 | 2 | 82.91 | 89.67 | 95.49 | | 6.93 | | 15.00 |
| 52 | Heptachlor epoxide | 385.8160 | 10.531 | 182.9/154.9 | 182.9/118.9 | Y=195.51x+170.58 | 0.9983 | 2.00~435.72 | 2 | 71.34 | 87.58 | 90.46 | | 10.83 | | 14.17 |
| 53 | Triadimenol^a^ | 295.1088 | 10.603 | 168.0/70.0 | 128.0/65.0 | Y=1381.13x+5996.14 | 0.9991 | 5.00~539.45 | 5 | 76.45 | 89.48 | 94.32 | | 2.66 | | 11.41 |
| 54 | [Procymidone](file:///D:\AN\Dict\8.9.4.0\resultui\html\index.html#/javascript:;) | 283.0167 | 10.705 | 282.8/96.0 | 282.8/68.1 | Y=1142.73x-1380.39 | 0.9996 | 1.00~449.06 | 1 | 92.00 | 94.78 | 98.32 | | 3.67 | | 8.76 |
| 55 | Triflumizole | 345.0856 | 10.486 | 206.0/179.0 | 206.0/186.0 | Y=820.90x-1609.10 | 0.9997 | 5.00~491.36 | 5 | 94.76 | 93.21 | 90.31 | | 8.82 | | 13.86 |
| 56 | Methidathion^a^ | 301.9619 | 10.806 | 144.9/85.0 | 144.9/58.1 | Y=3447.40x-10688.01 | 0.9995 | 10.00~488.51 | 10 | 81.21 | 87.34 | 90.10 | | 8.31 | | 7.00 |
| 57 | [Chlordan](file:///D:\AN\Dict\8.9.4.0\resultui\html\index.html#/javascript:;) | 278.0853 | 10.826 | 271.9/236.9 | 372.9/265.9 | Y=38.47x-65.39 | 0.9993 | 20.00~516.56 | 20 | 69.88 | 78.30 | 84.75 | | 7.03 | | 13.41 |
| 58 | [Fenoxaprop-p-ethyl](file:///D:\AN\Dict\8.9.4.0\resultui\html\index.html#/javascript:;) | 333.0404 | 10.861 | 160.1/72.1 | 72.0/56.0 | Y=2031.03x-2622.06 | 0.9992 | 2.00~545.71 | 2 | 94.68 | 89.54 | 90.29 | | 5.53 | | 13.33 |
| 59 | [Flumetralin](file:///D:\AN\Dict\8.9.4.0\resultui\html\index.html#/javascript:;) | 421.0452 | 11.049 | 143.0/107.1 | 143.0/117.0 | Y=1032.24x-11471.87 | 0.9964 | 1.00~529.36 | 1 | 83.27 | 80.32 | 89.49 | | 7.41 | | 11.23 |
| 60 | [Endosulfan](file:///D:\AN\Dict\8.9.4.0\resultui\html\index.html#/javascript:;) | 403.8169 | 11.081 | 194.0/159.0 | 194.9/125.0 | Y=85.04x+797.57 | 0.9994 | 2.00~485.32 | 2 | 91.28 | 94.32 | 95.69 | | 7.53 | | 11.18 |
| 61 | Fenamiphos^a^ | 303.1058 | 11.077 | 154.0/139.0 | 217.0/202.1 | Y=612.16x-462.43 | 0.9997 | 5.00~531.88 | 5 | 81.59 | 98.47 | 95.45 | | 6.69 | | 13.31 |
| 62 | Hexaconazole^a^ | 313.0749 | 11.751 | 231.0/175.0 | 256.0/159.0 | Y=262.27x-3.19 | 0.9997 | 1.00~497.64 | 1 | 78.79 | 89.23 | 90.48 | | 7.86 | | 12.73 |
| 63 | [Isoprothiolane](file:///D:\AN\Dict\8.9.4.0\resultui\html\index.html#/javascript:;) | 290.0647 | 11.257 | 162.1/85.0 | 162.1/134.0 | Y=1245.73x-2086.27 | 0.9996 | 2.00~483.39 | 2 | 87.45 | 93.84 | 89.38 | | 6.12 | | 15.19 |
| 64 | Profenofos^a^ | 371.9351 | 11.369 | 338.8/268.7 | 207.9/63.0 | Y=523.48x-1252.13 | 0.9989 | 1.00~497.96 | 1 | 98.45 | 89.67 | 93.60 | | 7.37 | | 15.68 |
| 65 | Pretilachlor^a^ | 311.1652 | 11.396 | 162.1/132.2 | 162.1/147.2 | Y=1474.70x-1258.58 | 0.9997 | 2.00~452.88 | 2 | 78.56 | 98.30 | 94.63 | | 4.35 | | 15.35 |
| 66 | [Endrin](file:///D:\AN\Dict\8.9.4.0\resultui\html\index.html#/javascript:;) | 377.8706 | 12.03 | 262.8/193.0 | 244.8/173.0 | Y=242.82x-58.52 | 0.9997 | 1.00~241.06 | 1 | 80.17 | 94.30 | 92.10 | | 7.47 | | 16.20 |
| 67 | [Dieldrin](file:///D:\AN\Dict\8.9.4.0\resultui\html\index.html#/javascript:;) | 377.8706 | 11.683 | 262.9/193.0 | 277.0/241.0 | Y=236.22x-132.94 | 0.9996 | 1.00~242.13 | 1 | 84.62 | 89.43 | 88.72 | | 4.32 | | 11.76 |
| 68 | Myclobutanil^a^ | 288.1142 | 11.54 | 179.0/125.1 | 150.0/123.0 | Y=2109.87x-1930.23 | 0.9995 | 2.00~520.24 | 2 | 93.50 | 92.52 | 96.78 | | 6.03 | | 15.37 |
| 69 | [Flusilazole](file:///D:\AN\Dict\8.9.4.0\resultui\html\index.html#/javascript:;) | 315.1003 | 11.554 | 233.0/165.1 | 314.7/232.9 | Y=887.29987.84x- | 0.9993 | 5.00~530.85 | 5 | 79.35 | 80.56 | 87.49 | | 7.55 | | 13.09 |
| 70 | Chlorfenapyr | 405.9605 | 12.071 | 136.9/102.0 | 246.9/227.0 | Y=95.54x-195.23 | 0.9997 | 5.00~487.33 | 5 | 104.28 | 99.23 | 97.47 | | 2.94 | | 14.68 |
| 71 | Oxadixyl | 278.1267 | 12.127 | 163.0/132.1 | 163.0/117.1 | Y=1479.92x-3051.08 | 0.9990 | 2.00~502.32 | 2 | 78.54 | 90.64 | 96.07 | | 5.76 | | 8.13 |
| 72 | Clethodim^a^ | 359.1322 | 12.389 | 205.0/176.0 | 164.0/81.0 | Y=205.21x-2868.19 | 0.9958 | 10.00~474.98 | 10 | 79.48 | 86.04 | 88.59 | | 3.98 | | 9.10 |
| 73 | Triazophos^a^ | 313.0650 | 12.43 | 161.2/134.2 | 161.1/106.1 | Y=778.51x-2480.94 | 0.9994 | 1.00~663.97 | 1 | 101.11 | 87.40 | 88.03 | | 5.06 | | 8.96 |
| 74 | Ediphenghos | 310.0251 | 12.795 | 172.9/109.0 | 108.9/65.1 | Y=3306.85x-17702.79 | 0.9988 | 1.00~584.56 | 1 | 103.72 | 98.34 | 89.68 | | 2.26 | | 10.60 |
| 75 | p,p'-DDT | 351.9147 | 12.878 | 235.0/165.1 | 235.0/199.1 | Y=3554.04x-3712.00 | 0.9995 | 2.00~258.62 | 2 | 103.40 | 92.49 | 96.48 | | 5.59 | | 11.74 |
| 76 | Tebuconazole^a^ | 307.1451 | 13.058 | 250.0/125.0 | 125.0/89.0 | Y=1352.29x-3380.57 | 0.9992 | 2.00~585.84 | 2 | 67.50 | 79.40 | 86.84 | | 6.70 | | 12.08 |
| 77 | Propargite | 350.1552 | 13.252 | 135.0/107.1 | 135.0/77.1 | Y=924.49x+3511.11 | 0.9981 | 1.00~498.75 | 1 | 121.40 | 112.32 | 104.67 | | 1.63 | | 15.60 |
| 78 | Biobenzfurethrin | 338.1882 | 13.255 | 171.0/128.1 | 123.0/81.1 | Y=1321.07x-3396.37 | 0.9996 | 2.00~664.79 | 2 | 104.43 | 102.43 | 89.35 | | 3.64 | | 13.83 |
| 79 | [Iprodione](file:///D:\AN\Dict\8.9.4.0\resultui\html\index.html#/javascript:;) | 329.0334 | 13.507 | 313.8/55.9 | 313.8/244.9 | Y=173.34x-1570.30 | 0.9955 | 2.00~530.08 | 2 | 107.50 | 103.48 | 95.69 | | 8.09 | | 12.10 |
| 80 | [Carbosulfan](file:///D:\AN\Dict\8.9.4.0\resultui\html\index.html#/javascript:;) | 380.2134 | 13.75 | 118.0/76.0 | 149.0/77.0 | Y=736.91x-22581.37 | 0.9661 | 2.00~572.05 | 2 | 102.60 | 94.30 | 97.04 | | 1.76 | | 15.52 |
| 81 | Phosmet^a^ | 301.0174 | 14.02 | 160.0/77.1 | 160.0/133.1 | Y=3642.63x-20201.00 | 0.9990 | 1.00~574.93 | 1 | 82.56 | 89.48 | 98.39 | | 6.15 | | 15.15 |
| 82 | [Bromopropylate](file:///D:\AN\Dict\8.9.4.0\resultui\html\index.html#/javascript:;) | 425.9466 | 13.66 | 183.0/155.0 | 185.0/157.0 | Y=1442.53x-1251.04 | 0.9995 | 1.00~502.21 | 1 | 69.50 | 78.53 | 87.1- | | 2.50 | | 15.80 |
| 83 | Bifenthrin | 422.1260 | 13.927 | 181.1/165.1 | 166.0/165.1 | Y=6066.44x-2921.73 | 0.9995 | 5.00~264.62 | 5 | 79.47 | 81.25 | 89.43 | | 4.68 | | 16.03 |
| 84 | [Fenpropathrin](file:///D:\AN\Dict\8.9.4.0\resultui\html\index.html#/javascript:;) | 349.1678 | 13.92 | 208.0/181.1 | 181.1/152.1 | Y=511.83x-731.31 | 0.9996 | 2.00~260.51 | 2 | 118.40 | 105.19 | 98.30 | | 1.23 | | 12.69 |
| 85 | Phosalone^a^ | 366.9869 | 14.386 | 182.0/111.0 | 182.0/75.1 | Y=2184.69x-4026.47 | 0.9993 | 2.00~458.49 | 2 | 80.51 | 84,29 | 89.30 | | 6.67 | | 11.11 |
| 86 | Cyfluthrin and Beta-cyfluthrin | 449.1006 | 14.94 | 207.9/181.1 | 196.9/141.1 | Y=1594.64x-4749.37 | 0.9992 | 3.00~608.14 | 3 | 103.76 | 105.54 | 102.34 | | 8.19 | | 12.66 |
| 87 | [Dechlorane](file:///D:\AN\Dict\8.9.4.0\resultui\html\index.html#/javascript:;) | 539.6262 | 14.711 | 271.8/236.8 | 273.8/238.8 | Y=4020.01x-5577.97 | 0.9995 | 2.00~483.71 | 2 | 108.40 | 94.12 | 91.05 | | 3.58 | | 12.31 |
| 88 | Biphentriazol^a^ | 337.1790 | 15.333 | 170.1/115.0 | 170.1/141.1 | Y=3537.44x-2822.49 | 0.9996 | 5.00~241.59 | 5 | 117.40 | 104.12 | 98.01 | | 6.40 | | 14.16 |
| 89 | Cyfluthrin and Beta-cyfluthrin | 433.0648 | 16.052 | 162.9/127.0 | 162.9/90.9 | Y=549.78x-2805.10 | 0.9993 | 10.00~599.10 | 10 | 103.11 | 102.84 | 96.01 | | 4.62 | | 11.29 |
| 90 | Pyridaben^a^ | 364.1376 | 15.325 | 147.2/117.1 | 147.2/132.2 | Y=4510.27x-29644.92 | 0.9996 | 2.00~526.63 | 2 | 80.51 | 96.65 | 98.39 | | 5.70 | | 14.69 |
| 91 | Coumaphos^a^ | 362.0145 | 15.695 | 361.9/109.0 | 210.0/182.0 | Y=795.15x-2799.64 | 0.9990 | 1.00~518.80 | 1 | 115.80 | 103.29 | 99.37 | | 2.90 | | 11.32 |
| 92 | Prochloraz | 375.0308 | 15.737 | 180.0/138.0 | 310.0/69.8 | Y=480.54x-2923.93 | 0.9991 | 2.00~610.77 | 2 | 104.31 | 96.38 | 98.47 | | 6.23 | | 15.60 |
| 93 | Permethrin | 390.0789 | 15.524 | 162.9/127.1 | 162.9/91.1 | Y=544.14x+58.15 | 0.9996 | 1.00~475.77 | 1 | 85.32 | 98.39 | 99.23 | | 7.34 | | 14.77 |
| 94 | Fenbuconazole^a^ | 336.1142 | 16.233 | 162.9/127.1 | 162.9/91.1 | Y=2781.97x-8907.81 | 0.9994 | 1.00~534.04 | 1 | 87.45 | 98.36 | 99.12 | | 2.27 | | 12.01 |
| 95 | Cypermethrin and beta-cypermethrin | 415.0742 | 16.366 | 163.0/127.0 | 163.0/91.0 | Y=716.84x-4425.84 | 0.9994 | 1.00~516.58 | 1 | 103.68 | 101.09 | 98.46 | | 4.28 | | 7.36 |
| 96 | Ethofenprox | 376.2038 | 16.226 | 163.0/107.1 | 163.0/135.1 | Y=8874.38x-8347.06 | 0.9996 | 1.00~321.31 | 1 | 69.88 | 78.17 | 90.06 | | 8.73 | | 14.46 |
| 97 | Flucythrinate | 451.1595 | 16.592 | 199.1/107.1 | 199.1/157.0 | Y=2921.13x-11248.14 | 0.9994 | 5.00~449.87 | 5 | 76.44 | 90.49 | 95.69 | | 2.40 | | 7.60 |
| 98 | Fenvalerate and S-fenvalerate | 419.1288 | 17.336 | 167.0/125.2 | 181.0/151.8 | Y=1723.59x-6756.60 | 0.9992 | 5.00~496.48 | 5 | 102.59 | 97.02 | 94.50 | | 6.79 | | 14.01 |
| 99 | [Fluvalinate](file:///D:\AN\Dict\8.9.4.0\resultui\html\index.html#/javascript:;) | 502.1271 | 17.426 | 249.9/55.1 | 249.9/200.2 | Y=1861.80x-14195.21 | 0.9984 | 5.00~515.31 | 5 | 67.55 | 87.40 | 94.29 | | 3.14 | | 13.93 |
| 100 | Difenoconazole^a^ | 405.0647 | 17.678 | 322.9/265.0 | 264.9/201.9 | Y=2596.17x-6167.59 | 0.9994 | 2.00~510.89 | 2 | 103.76 | 99.30 | 94.46 | | 5.32 | | 11.83 |
| 101 | [Deltamethrin](file:///D:\AN\Dict\8.9.4.0\resultui\html\index.html#/javascript:;) | 502.9732 | 17.735 | 181.0/152.1 | 252.8/172.0 | Y=571.86x-5134.35 | 0.9987 | 5.00~563.26 | 5 | 92.45 | 98.38 | 96.12 | | 1.87 | | 11.78 |

^a^ Represents that the pesticide residues were tested by both LC-MS/MS and GC-MS/MS.

^*^Represents quantitative ion pairs.

**Table S2 Retention time, detected ion pairs, Linear equation, Correlation coefficient, Linearity range, LOQ, Mean recovery,** **Intra-day RSD and Inter-day RSD for pesticide detection by LC-MS-MS**

| Number | Compound | Mw | Retention time (min) | Product ion pair ^*^ (*m/z*) | Product ion pair (*m/z*) | Linear | r^2^ | Range（ng/mL） | LOQ (ng/mL) | Mean recovery (n=3, %) | | | Intra-day RSD (n=3, %) | Inter-day RSD (n=3, %) |
| --- | --- | --- | --- | --- | --- | --- | --- | --- | --- | --- | --- | --- | --- | --- |
|  |  |  |  |  |  |  |  |  |  | 20 ng/mL | 100 ng/mL | 200 ng/mL |  |  |
| 1 | Methamidophos^a^ | 141.0013 | 1.200 | 142.0/93.9 | 142.0/124.9 | y=174.748x-310.057 | 0.9951 | 1.00~152.20 | 1 | 75.09 | 89.35 | 96.49 | 3.60 | 15.44 |
| 2 | Methomyl | 162.0463 | 3.190 | 163.0/106.0 | 163.0/88.0 | y=44.9927x-53.5949 | 0.9944 | 2.00~153.60 | 2 | 85.87 | 98.38 | 96.29 | 7.88 | 16.29 |
| 3 | Cyromazine | 166.0967 | 1.060 | 167.0/60.2 | 167.0/108.1 | y=119.182x-446.5 | 0.9995 | 10.00~157.70 | 10 | 107.30 | 104.23 | 102.01 | 11.18 | 11.85 |
| 4 | Acephate^a^ | 183.0119 | 1.350 | 184.1/143.0 | 184.1/125.1 | y=114.371x+2006.83 | 0.9067 | 1.00~167.30 | 1 | 117.84 | 103.58 | 102.65 | 8.85 | 15.46 |
| 5 | Propamocarb | 188.1525 | 1.980 | 189.1/102.0 | 189.1/144.0 | y=736.626x + -1096.76 | 0.9965 | 2.00~154.70 | 2 | 83.34 | 85.13 | 87.49 | 7.70 | 13.18 |
| 6 | Carbendazim | 191.0695 | 2.930 | 192.1/160.1 | 192.1/132.1 | y=401.125x-1563.53 | 0.9987 | 5.00~156.70 | 5 | 68.30 | 89.75 | 92.19 | 7.26 | 14.77 |
| 7 | Isoprocarb | 193.1103 | 7.840 | 194.1/95.1 | 194.1/137.1 | y=311.024x-407.172 | 0.9953 | 2.00~153.90 | 2 | 77.87 | 90.20 | 97.13 | 5.87 | 12.22 |
| 8 | Pyrimethanil | 199.1109 | 8.160 | 200.0/107.0 | 200.0/82.0 | y=129.728x-645.732 | 0.9945 | 1.00~153.30 | 1 | 118.23 | 103.94 | 96.49 | 8.59 | 13.19 |
| 9 | Carbary | 201.0790 | 7.320 | 202.0/145.0 | 202.0/117.0 | y=45.6539x-657.184 | 0.9582 | 2.00~156.70 | 2 | 81.90 | 94.58 | 98.64 | 5.18 | 9.05 |
| 10 | Dinotefuran | 202.1066 | 1.950 | 203.1/114.1 | 203.1/129.0 | y= 64.3239x-476.504 | 0.9963 | 1.00~155.90 | 1 | 76.84 | 88.47 | 89.57 | 3.63 | 10.69 |
| 11 | Aldicarb | 190.0776 | 5.870 | 213.1/116.1 | 213.1/89.1 | y=1.29231x-43.9041 | 0.9222 | 1.00~170.70 | 1 | 67.39 | 87.45 | 98.48 | 4.60 | 11.83 |
| 12 | Omethoate^a^ | 213.0225 | 1.530 | 214.1/183.1 | 214.1/125.1 | y=158.447x-799.612 | 0.9933 | 1.00~161.90 | 1 | 118.87 | 103.76 | 98.38 | 4.46 | 12.17 |
| 13 | Pymetrozine | 217.0964 | 1.310 | 218.0/105.0 | 218.0/79.0 | y=199.342x-528.679 | 0.9953 | 1.00~155.30 | 1 | 92.34 | 94.58 | 98.46 | 6.10 | 15.69 |
| 14 | Dichlorvos^a^ | 219.9459 | 6.490 | 221.0/109.0 | 221.0/79.0 | y=15.8363x+35.2119 | 0.9618 | 1.00~167.20 | 1 | 69.76 | 89.53 | 90.47 | 7.24 | 13.92 |
| 15 | Carbofuran^a^ | 221.1052 | 6.940 | 222.1/165.1 | 222.1/123.0 | y=232.003x-453.307 | 0.9961 | 1.00~155.50 | 1 | 77.23 | 87.40 | 94.16 | 7.58 | 12.19 |
| 16 | Acetamiprid | 222.0672 | 5.050 | 223.0/126.0 | 223.0/56.1 | y= 342.011x-733.859 | 0.9948 | 2.00~153.10 | 2 | 89.06 | 90.48 | 96.39 | 11.10 | 15.61 |
| 17 | Monocrotophos^a^ | 223.0610 | 3.210 | 224.1/127.1 | 224.1/98.1 | y= 235.516x-934.028 | 0.9977 | 1.00~155.00 | 1 | 74.85 | 89.37 | 94.09 | 9.33 | 15.24 |
| 18 | Cyprodinil | 225.1266 | 9.780 | 226.0/93.0 | 226.0/108.0 | y= 154.339x-526.118 | 0.9904 | 2.00~155.30 | 2 | 109.84 | 105.38 | 102.18 | 7.60 | 15.89 |
| 19 | Dimethoate^a^ | 228.9996 | 4.880 | 230.1/199.0 | 230.1/125.0 | y= 207.865x-976.211 | 0.9966 | 1.00~153.10 | 1 | 93.11 | 97.56 | 99.03 | 4.02 | 16.12 |
| 20 | Carbofuran-3-hydroxy | 237.1001 | 4.530 | 238.0/181.0 | 238.0/163.0 | y=81.6781x-309.794 | 0.9981 | 2.00~157.10 | 2 | 87.47 | 89.32 | 90.34 | 3.65 | 12.78 |
| 21 | Pirimicarb^a^ | 238.1430 | 5.530 | 239.1/72.0 | 239.1/182.1 | y=819x-1619.71 | 0.9972 | 1.00~158.80 | 1 | 115.76 | 106.24 | 101.02 | 7.30 | 8.63 |
| 22 | Ethoprophos^a^ | 242.0564 | 9.270 | 243.2/131.0 | 243.2/97.0 | y= 217.334x-1371.71 | 0.9948 | 1.00~151.80 | 1 | 80.06 | 89.34 | 85.38 | 5.53 | 13.01 |
| 23 | Fonofos^a^ | 246.0302 | 10.930 | 247.1/137.0 | 247.1/109.0 | y= 62.3933x-678.666 | 0.9769 | 2.00~156.00 | 2 | 114.51 | 105.37 | 106.97 | 8.19 | 10.21 |
| 24 | Forchlorfenuron | 247.0512 | 7.350 | 248.1/129.0 | 248.1/93.0 | y= 401.1x-1616.34 | 0.996 | 1.00~154.00 | 1 | 109.11 | 107.36 | 106.32 | 4.04 | 13.54 |
| 25 | Clothianidin | 249.0087 | 4.450 | 250.0/169.0 | 250.0/132.0 | y=37.263x-94.2173 | 0.9942 | 1.00~157.60 | 1 | 115.94 | 108.03 | 103.45 | 8.22 | 14.65 |
| 26 | Thiacloprid | 252.0236 | 5.770 | 253.0/126.0 | 253.0/90.1 | y=533.025x-715.741 | 0.9968 | 1.00~154.60 | 1 | 107.85 | 102.01 | 97.24 | 4.01 | 9.58 |
| 27 | Phosfolan | 255.0153 | 5.380 | 256.0/140.0 | 256.0/168.0 | y= 214.288x-426.581 | 0.9974 | 1.00~158.90 | 1 | 85.36 | 90.38 | 97.38 | 8.49 | 11.59 |
| 28 | Propyzamide | 255.0218 | 9.300 | 256.1/190.0 | 256.1/173.0 | y= 38.6957x-347.237 | 0.9492 | 2.00~140.60 | 2 | 85.23 | 90.20 | 93.56 | 7.11 | 16.04 |
| 29 | Imidacloprid | 255.0523 | 4.700 | 256.1/209.1 | 256.1/175.1 | y=42.6526x-54.6693 | 0.9916 | 5.00~153.10 | 5 | 94.87 | 97.13 | 98.30 | 7.96 | 15.71 |
| 30 | Trichlorfon | 255.9226 | 4.200 | 257.0/109.0 | 257.0/79.0 | y=73.5238x-92.7058 | 0.9945 | 2.00~157.50 | 2 | 70.11 | 80.50 | 93.03 | 3.52 | 14.10 |
| 31 | Phorate^a^ | 260.0128 | 11.080 | 261.0/75.0 | 261.0/97.0 | y=45.3481x-518.4 | 0.9625 | 1.00~138.80 | 1 | 99.11 | 101.99 | 99.05 | 7.13 | 10.45 |
| 32 | Diethofencarb | 267.1471 | 8.770 | 268.0/226.0 | 268.0/124.0 | y=92.8966x-72.8754 | 0.9966 | 2.00~159.40 | 2 | 113.02 | 106.83 | 98.45 | 4.85 | 12.63 |
| 33 | Cadusafos^a^ | 270.0877 | 10.690 | 271.1/159.0 | 271.1/131.0 | y=437.559x-877.515 | 0.995 | 1.00~156.80 | 1 | 76.11 | 89.46 | 93.97 | 6.44 | 9.18 |
| 34 | Fenthion^a^ | 278.0200 | 10.590 | 279.1/247.1 | 279.1/169.1 | y=11.7836x-123.708 | 0.9764 | 1.00~174.00 | 1 | 114.90 | 107.82 | 102.93 | 3.73 | 14.33 |
| 35 | Metalaxyl^a^ | 279.1471 | 7.550 | 280.1/220.1 | 280.1/192.1 | y=422.83x-1106.73 | 0.9961 | 1.00~159.00 | 1 | 75.09 | 98.38 | 97.73 | 3.98 | 10.76 |
| 36 | Pendimethalin^a^ | 281.1376 | 12.290 | 282.2/212.2 | 282.2/194.1 | y= 59.227x-881.826 | 0.9721 | 1.00~158.00 | 1 | 91.94 | 94.60 | 98.57 | 2.89 | 14.69 |
| 37 | Penconazole | 283.0643 | 9.700 | 284.0/70.1 | 284.0/159.0 | y=369.887x-203.932 | 0.9815 | 10.00~145.30 | 10 | 83.11 | 90.29 | 89.30 | 2.36 | 18.16 |
| 38 | Terbufos^a^ | 288.0441 | 12.070 | 289.0/57.2 | 289.0/103.0 | y=391.675x-649.147 | 0.9948 | 5.00~156.30 | 5 | 75.34 | 80.46 | 89.47 | 3.50 | 14.30 |
| 39 | Myclobutanil^a^ | 288.1142 | 9.120 | 289.1/70.2 | 289.1/125.1 | y=190.578x+64.0642 | 0.9898 | 5.00~151.20 | 5 | 68.36 | 89.45 | 93.76 | 3.84 | 9.06 |
| 40 | Isocarbofos^a^ | 289.0538 | 10.790 | 291.1/121.1 | 191.1/231.1 | y=62.8161-267.841 | 0.9796 | 1.00~159.80 | 1 | 82.94 | 87.38 | 89.46 | 7.36 | 13.64 |
| 41 | Thiamethoxam | 291.0193 | 3.900 | 292.0/211.2 | 292.0/132.0 | y=122.285x-262.599 | 0.9997 | 1.00~158.90 | 1 | 72.06 | 98.64 | 93.57 | 5.59 | 16.94 |
| 42 | Triadimefon^a^ | 293.0931 | 9.190 | 294.1/69.3 | 294.1/197.2 | y= 143.519x+832.411 | 0.986 | 1.00~155.30 | 1 | 103.47 | 103.09 | 102.41 | 3.86 | 16.61 |
| 43 | Paclobutrazol | 293.1295 | 8.440 | 294.1/70.2 | 294.1/125.1 | y= 497.124x-1520.99 | 0.9968 | 2.00~154.90 | 2 | 90.39 | 97.38 | 90.37 | 7.28 | 17.46 |
| 44 | Triadimenol^a^ | 295.1088 | 8.450 | 296.1/70.2 | 296.1/99.1 | y=172.171x-2221.34 | 0.9731 | 2.00~149.10 | 2 | 102.76 | 98.49 | 102.28 | 6.91 | 13.02 |
| 45 | Phoxim | 298.0541 | 11.060 | 299.0/153.0 | 299.0/129.0 | y= 24.4373x-88.9975 | 0.9443 | 5.00~179.90 | 5 | 71.87 | 89.48 | 92.76 | 7.56 | 16.63 |
| 46 | Phosphamidon^a^ | 299.0689 | 5.970 | 300.1/174.1 | 300.1/127.1 | y= 254.388x-1092.45 | 0.9976 | 5.00~155.80 | 5 | 66.02 | 87.59 | 88.42 | 7.79 | 14.35 |
| 47 | Bifenazate^a^ | 300.1474 | 9.550 | 301.1/198.0 | 301.1/170.0 | y=150.179x+673.06 | 0.9917 | 1.00~153.80 | 1 | 74.76 | 86.05 | 89.30 | 4.45 | 15.94 |
| 48 | Fenhexamid | 301.0636 | 9.290 | 302.1/97.2 | 302.1/55.3 | y= 124.371x+1149.34 | 0.9684 | 1.00~153.80 | 1 | 91.76 | 89.10 | 93.47 | 5.89 | 9.39 |
| 49 | Methidathion^a^ | 301.9619 | 8.720 | 303.0/85.1 | 303.0/145.0 | y= 143.973x-480.625 | 0.9962 | 2.00~153.30 | 2 | 107.76 | 105.69 | 102.61 | 3.27 | 10.36 |
| 50 | Clofentezine | 302.0126 | 10.910 | 303.0/138.0 | 303.0/102.0 | y=15.3842x-89.5101 | 0.987 | 1.00~149.80 | 1 | 68.76 | 89.29 | 90.47 | 4.42 | 10.22 |
| 51 | Fenamiphos^a^ | 303.1058 | 9.040 | 304.1/217.1 | 304.1/202.1 | y=345.136x-500.569 | 0.99 | 1.00~156.70 | 1 | 66.11 | 89.49 | 95.21 | 5.85 | 11.86 |
| 52 | Diazinon^a^ | 304.1010 | 10.750 | 305.1/169.0 | 305.1 > 96.9 | y=127.638x-1202.56 | 0.9897 | 1.00~157.10 | 1 | 67.51 | 89.49 | 90.57 | 7.70 | 13.00 |
| 53 | Fenazaquin | 306.1732 | 12.290 | 307.2/161.0 | 307.2/57.2 | y=0.400754x+33.500 | 0.9086 | 1.00~168.90 | 1 | 111.47 | 104.30 | 102.02 | 4.80 | 13.34 |
| 54 | Tebuconazole^a^ | 307.1451 | 9.440 | 308.0/70.1 | 308.0/125.0 | y=496.298x-15.6863 | 0.9931 | 1.00~156.80 | 1 | 115.85 | 102.78 | 98.37 | 5.42 | 16.86 |
| 55 | Hexaconazole^a^ | 313.0749 | 9.710 | 314.0/70.1 | 314.0/159.0 | y=340.364x-554.803 | 0.9926 | 2.00~150.10 | 2 | 60.11 | 89.58 | 93.28 | 5.68 | 15.09 |
| 56 | Isazophos | 313.0417 | 10.160 | 314.0/162.1 | 314.0/120.0 | y=347.965x-876.086 | 0.9954 | 5.00~157.60 | 5 | 74.11 | 89.46 | 85.04 | 6.60 | 13.36 |
| 57 | Triazophos^a^ | 313.0650 | 9.770 | 314.1/161.9 | 314.1/118.9 | y= 447.255x-269.556 | 0.9954 | 1.00~152.00 | 1 | 107.11 | 102.71 | 98.43 | 2.31 | 16.78 |
| 58 | Phosmet^a^ | 301.0174 | 8.970 | 318.0/160.0 | 318.0/77.0 | y=72.368x+387.242 | 0.9454 | 2.00~149.00 | 2 | 86.76 | 89.59 | 97.40 | 9.73 | 16.41 |
| 59 | Pyriproxifen | 321.1365 | 12.060 | 322.1/227.1 | 322.1/96.0 | y=185.319x-933.986 | 0.9933 | 1.00~152.50 | 1 | 90.51 | 92.48 | 96.59 | 8.01 | 17.06 |
| 60 | Sulfotep^a^ | 322.0227 | 11.000 | 323.0/97.0 | 323.0/171.0 | y=304.365x-709.623 | 0.9936 | 1.00~167.20 | 1 | 74.94 | 86.70 | 89.38 | 7.89 | 17.29 |
| 61 | Cyazofamid | 324.0448 | 10.310 | 325.0/107.9 | 325.0/261.0 | y=83.5831x-1282.74 | 0.9738 | 5.00~147.70 | 5 | 70.47 | 87.49 | 89.32 | 7.57 | 13.95 |
| 62 | Benalaxyl | 325.1678 | 10.340 | 326.1/148.0 | 326.1/91.0 | y= 508.276x-2652.96 | 0.996 | 10.00~153.90 | 10 | 91.85 | 96.38 | 91.20 | 7.46 | 9.80 |
| 63 | Malathion | 330.0361 | 9.710 | 331.0/127.0 | 331.0/99.0 | y= 382.982x-3195.54 | 0.9868 | 1.00~148.50 | 1 | 106.51 | 102.31 | 98.70 | 7.47 | 12.75 |
| 64 | Zoxamide^a^ | 335.0247 | 10.710 | 336.0/187.1 | 336.0/159.0 | y=242.655x-1197.23 | 0.9963 | 2.00~153.50 | 2 | 70.03 | 103.20 | 95.29 | 10.13 | 12.14 |
| 65 | Fenbuconazole^a^ | 336.1142 | 9.640 | 337.0/70.1 | 337.0/125.0 | y=147.656x+1788.97 | 0.9873 | 1.00~159.70 | 1 | 77.03 | 84.39 | 87.49 | 7.95 | 14.96 |
| 66 | Biphentriazol^a^ | 337.1790 | 9.720 | 338.1/99.1 | 338.1/70.1 | y=127.062x+691.768 | 0.9624 | 1.00~141.20 | 1 | 116.09 | 102.12 | 98.10 | 5.25 | 13.18 |
| 67 | Propiconazole | 341.0698 | 9.990 | 342.0/69.0 | 342.0/159.0 | y=265.957x-298.14 | 0.9882 | 1.00~151.40 | 1 | 73.90 | 89.18 | 93.48 | 2.43 | 14.26 |
| 68 | Boscalid | 342.0327 | 9.260 | 342.9/139.9 | 342.9/307.0 | y=65.1848x+701.663 | 0.9784 | 2.00~143.50 | 2 | 109.03 | 104.30 | 93.20 | 10.73 | 11.46 |
| 69 | Thiophanate | 342.0456 | 6.670 | 343.0/151.0 | 343.0/93.0 | y=409.718x-736.875 | 0.9963 | 5.00~155.10 | 5 | 93.03 | 98.30 | 92.13 | 7.53 | 14.79 |
| 70 | Chlorpyrifos | 348.9263 | 12.290 | 349.9/97.0 | 349.9/198.0 | y=22.1864x-77.9013 | 0.9896 | 1.00~172.50 | 1 | 83.09 | 89.30 | 95.60 | 8.87 | 15.90 |
| 71 | Fenpropathrin | 349.1678 | 12.260 | 350.1/97.0 | 350.1/125.0 | y= 21.5954x-125.104 | 0.9683 | 1.00~155.30 | 1 | 84.03 | 98.40 | 92.19 | 5.51 | 10.83 |
| 72 | Hexythiazox | 352.1012 | 12.280 | 353.0/228.1 | 353.0/168.1 | y=105.052x-564.919 | 0.9955 | 2.00~159.90 | 2 | 91.30 | 93.49 | 95.69 | 5.52 | 12.84 |
| 73 | Tebufenozide | 352.2151 | 10.100 | 353.1/133.0 | 353.1/297.1 | y=317.867x-186.302 | 0.9895 | 1.00~146.70 | 1 | 82.30 | 89.40 | 93.48 | 8.05 | 17.29 |
| 74 | Clethodim^a^ | 359.1322 | 11.600 | 360.0/164.0 | 360.0/268.1 | y= 62.8926x-743.193 | 0.9801 | 2.00~144.00 | 2 | 117.03 | 107.21 | 102.81 | 5.98 | 10.96 |
| 75 | Coumaphos^a^ | 362.0145 | 10.800 | 363.0/307.0 | 363.0/289.0 | y=18.0328x-11.893 | 0.9287 | 2.00~131.90 | 2 | 76.36 | 87.39 | 88.42 | 3.40 | 15.35 |
| 76 | Pyridaben^a^ | 364.1376 | 13.110 | 365.1/147.1 | 365.1/309.1 | y= 761.085x-1543.8 | 0.9952 | 5.00~155.40 | 5 | 80.02 | 84.38 | 89.39 | 5.72 | 11.70 |
| 77 | Phosalone^a^ | 366.9869 | 11.110 | 367.9/181.9 | 367.9/110.9 | y= 74.1172x-293.643 | 0.9617 | 1.00~172.80 | 1 | 80.08 | 89.38 | 87.48 | 8.62 | 13.88 |
| 78 | Profenofos^a^ | 371.9351 | 11.400 | 372.9/127.9 | 372.9/302.6 | y=16.2924x-317.308 | 0.9678 | 1.00~177.90 | 1 | 94.23 | 94.39 | 94.81 | 5.58 | 10.43 |
| 79 | Pretilachlor^a^ | 311.1652 | 9.840 | 376.0/70.1 | 376.0/307.1 | y= 187.934x-1115.63 | 0.9892 | 3.00~147.70 | 3 | 84.84 | 94.39 | 98.63 | 6.85 | 15.58 |
| 80 | Haloxyfop | 375.0485 | 11.160 | 376.0/91.1 | 376.0/316.1 | y= 198.444x-432.575 | 0.9949 | 20.00~155.70 | 20 | 81.39 | 83.29 | 87.49 | 5.78 | 12.01 |
| 81 | Teflubenzuron | 379.9742 | 11.170 | 380.9/158.0 | 380.9/140.9 | y=2.20487x-9.37679 | 0.9573 | 5.00~168.70 | 5 | 106.39 | 98.39 | 96.30 | 8.34 | 14.69 |
| 82 | Dimethomorph | 387.1237 | 8.270 | 388.1/300.9 | 388.1/165.0 | y=37.1759x-256.846 | 0.9552 | 5.00~170.50 | 5 | 73.39 | 87.30 | 89.29 | 4.98 | 11.80 |
| 83 | Azoxystrobin | 403.1168 | 9.080 | 404.0/372.0 | 404.0/329.0 | y= 449.848x-1954.53 | 0.9988 | 2.00~156.00 | 2 | 113.06 | 83.29 | 87.62 | 5.78 | 15.23 |
| 84 | Difenoconazole* | 405.0647 | 10.420 | 406.0/251.1 | 406.0/111.1 | y=305.643x-640.625 | 0.9929 | 2.00~151.80 | 2 | 108.06 | 102.41 | 98.16 | 6.32 | 16.38 |
| 85 | Trifloxystrobin | 408.1297 | 11.360 | 409.0/186.0 | 409.0/145.0 | y=676.708x-4856.23 | 0.9992 | 1.00~157.20 | 1 | 78.47 | 89.39 | 89.32 | 3.49 | 11.15 |
| 86 | Spirodiclofen | 410.1052 | 13.270 | 411.1/71.2 | 411.1/313.0 | y=216.367x-537.364 | 0.9925 | 2.00~153.20 | 2 | 63.76 | 89.49 | 94.01 | 6.87 | 13.23 |
| 87 | Novaluron | 492.0123 | 13.270 | 493.0/158.0 | 493.0/141.0 | y=30.4961x-343.899 | 0.9936 | 1.00~158.00 | 1 | 82.76 | 89.38 | 99.48 | 4.55 | 17.81 |
| 88 | Metominostrobin | 284.1161 | 8.190 | 285.1/196.2 | 285.2/238.2 | y= 473.384x-951.063 | 0.9975 | 1.00~159.20 | 1 | 84.11 | 87.39 | 89.74 | 3.67 | 11.29 |
| 89 | Dimoxystrobin | 223.1109 | 9.900 | 327.1/205.2 | 327.1/116.1 | y= 422.934x-1980.47 | 0.9856 | 1.00~146.40 | 1 | 68.85 | 77.48 | 78.30 | 5.48 | 15.81 |
| 90 | Boscalid | 342.0327 | 10.440 | 368.0/145.1 | 368.0/205.1 | y=598.23x-1911.45 | 0.9973 | 1.00~154.10 | 1 | 89.02 | 85.58 | 87.37 | 7.32 | 12.05 |
| 91 | Pyraclostrobin | 387.0986 | 10.800 | 388.1/193.9 | 388.1/163.0 | y=211.859x-1154.81 | 0.9879 | 1.00~148.60 | 1 | 72.02 | 90.39 | 97.39 | 4.99 | 14.30 |
| 92 | Orysastrobin | 391.1856 | 9.140 | 329.2/205.3 | 329.2/116.2 | y=857.521x-2722.87 | 0.9965 | 1.00~155.50 | 1 | 93.84 | 88.49 | 94.37 | 6.20 | 10.74 |
| 93 | Enestroburin | 404.1629 | 11.880 | 400.1/178.2 | 400.1/137.1 | y=997.608x-3336.21 | 0.993 | 2.00~152.40 | 2 | 110.76 | 103.29 | 104.38 | 5.17 | 16.05 |
| 94 | Fluoxastrobin | 458.0783 | 9.890 | 459.0/427.0 | 459.0/188.0 | y=225.819x-946.463 | 0.9936 | 5.00~124.00 | 5 | 66.06 | 87.30 | 89.49 | 4.58 | 12.37 |

^a^ Represents that the pesticide residues were tested by both LC-MS/MS and GC-MS/MS.

^*^Represents quantitative ion pairs.

**Table S3 Sample information of Codonopsis Radix**

| Number | Base source | Production Place | Growth years | Collection date |
| --- | --- | --- | --- | --- |
| S1 | *Codonopsis pilosula* (Franch.) Nannf. | Gansu Province | 2 | 2019/11/28 |
| S2 | *Codonopsis pilosula* (Franch.) Nannf. | Gansu Province | 2 | 2019/11/28 |
| S3 | *Codonopsis pilosula* (Franch.) Nannf. | Gansu Province | 2 | 2019/11/28 |
| S4 | *Codonopsis pilosula* (Franch.) Nannf. | Gansu Province | 2 | 2019/11/28 |
| S5 | *Codonopsis pilosula* (Franch.) Nannf. | Gansu Province | 2 | 2019/11/9 |
| S6 | *Codonopsis pilosula* (Franch.) Nannf. | Gansu Province | 2 | 2019/11/23 |
| S7 | *Codonopsis pilosula* (Franch.) Nannf. | Gansu Province | 2 | 2019/11/23 |
| S8 | *Codonopsis pilosula* (Franch.) Nannf. | Gansu Province | 2 | 2019/11/23 |
| S9 | *Codonopsis pilosula* (Franch.) Nannf. | Gansu Province | 2 | 2019/11/23 |
| S10 | *Codonopsis pilosula* (Franch.) Nannf. | Gansu Province | 2 | 2019/11/23 |
| S11 | *Codonopsis pilosula* (Franch.) Nannf. | Gansu Province | 2 | 2019/11/23 |
| S12 | *Codonopsis pilosula* (Franch.) Nannf. | Gansu Province | 2 | 2019/11/21 |
| S13 | *Codonopsis pilosula* (Franch.) Nannf. | Gansu Province | 2 | 2019/11/23 |
| S14 | *Codonopsis pilosula* (Franch.) Nannf. | Gansu Province | 2 | 2019/11/23 |
| S15 | *Codonopsis pilosula* (Franch.) Nannf. | Gansu Province | 2 | 2019/11/23 |
| S16 | *Codonopsis pilosula* (Franch.) Nannf. | Gansu Province | 2 | 2019/11/23 |
| S17 | *Codonopsis pilosula* (Franch.) Nannf. | Gansu Province | 2 | 2019/11/23 |
| S18 | *Codonopsis pilosula* (Franch.) Nannf. | Gansu Province | 2 | 2019/11/23 |
| S19 | *Codonopsis pilosula* (Franch.) Nannf. | Gansu Province | 2 | 2019/11/23 |
| S20 | *Codonopsis pilosula* (Franch.) Nannf. | Gansu Province | 2 | 2019/11/23 |
| S21 | *Codonopsis pilosula* (Franch.) Nannf. | Gansu Province | 2 | 2019/11/23 |
| S22 | *Codonopsis pilosula* (Franch.) Nannf. | Gansu Province | 2 | 2019/11/23 |
| S23 | *Codonopsis pilosula* (Franch.) Nannf. | Gansu Province | 2 | 2019/11/23 |
| S24 | *Codonopsis pilosula* (Franch.) Nannf. | Gansu Province | 2 | 2019/11/23 |
| S25 | *Codonopsis pilosula* (Franch.) Nannf. | Gansu Province | 2 | 2019/11/23 |
| S26 | *Codonopsis pilosula* (Franch.) Nannf. | Gansu Province | 2 | 2019/11/23 |
| S27 | *Codonopsis pilosula* (Franch.) Nannf. | Gansu Province | 2 | 2019/11/29 |
| S28 | *Codonopsis pilosula* (Franch.) Nannf. | Gansu Province | 2 | 2019/11/23 |
| S29 | *Codonopsis pilosula* (Franch.) Nannf. | Gansu Province | 2 | 2019/11/23 |
| S30 | *Codonopsis pilosula* (Franch.) Nannf. | Gansu Province | 2 | 2019/11/23 |
| S31 | *Codonopsis pilosula* (Franch.) Nannf. | Gansu Province | 2 | 2019/11/23 |
| S32 | *Codonopsis pilosula* (Franch.) Nannf. | Gansu Province | 2 | 2019/11/23 |
| S33 | *Codonopsis pilosula* (Franch.) Nannf. | Gansu Province | 2 | 2019/11/23 |
| S34 | *Codonopsis pilosula* (Franch.) Nannf. | Gansu Province | 2 | 2019/11/23 |
| S35 | *Codonopsis pilosula* (Franch.) Nannf. | Gansu Province | 2 | 2019/11/23 |
| S36 | *Codonopsis pilosula* (Franch.) Nannf. | Gansu Province | 2 | 2019/11/28 |
| S37 | *Codonopsis pilosula* (Franch.) Nannf. | Gansu Province | 2 | 2019/11/28 |
| S38 | *Codonopsis pilosula* (Franch.) Nannf. | Gansu Province | 2 | 2019/11/11 |
| S39 | *Codonopsis pilosula* (Franch.) Nannf. | Gansu Province | 2 | 2019/11/11 |
| S40 | *Codonopsis pilosula* (Franch.) Nannf. | Gansu Province | 2 | 2019/11/11 |
| S41 | *Codonopsis pilosula* (Franch.) Nannf. | Gansu Province | 2 | 2019/11/11 |
| S42 | *Codonopsis pilosula* (Franch.) Nannf. | Gansu Province | 2 | 2019/11/11 |
| S43 | *Codonopsis pilosula* (Franch.) Nannf. | Gansu Province | 2 | 2019/11/13 |
| S44 | *Codonopsis pilosula* (Franch.) Nannf. | Gansu Province | 2 | 2019/11/13 |
| S45 | *Codonopsis pilosula* (Franch.) Nannf. | Gansu Province | 2 | 2019/11/14 |
| S46 | *Codonopsis pilosula* (Franch.) Nannf. | Gansu Province | 2 | 2019/11/14 |
| S47 | *Codonopsis pilosula* (Franch.) Nannf. | Gansu Province | 2 | 2019/11/13 |
| S48 | *Codonopsis pilosula* (Franch.) Nannf. | Gansu Province | 2 | 2019/11/13 |
| S49 | *Codonopsis pilosula* (Franch.) Nannf. | Gansu Province | 2 | 2019/11/13 |
| S50 | *Codonopsis pilosula* (Franch.) Nannf. | Gansu Province | 2 | 2019/11/16 |
| S51 | *Codonopsis pilosula* (Franch.) Nannf. | Gansu Province | 2 | 2019/11/16 |
| S52 | *Codonopsis pilosula* (Franch.) Nannf. | Gansu Province | 2 | 2019/11/16 |
| S53 | *Codonopsis pilosula* (Franch.) Nannf. | Gansu Province | 2 | 2019/11/16 |
| S54 | *Codonopsis pilosula* (Franch.) Nannf. | Gansu Province | 2 | 2019/11/16 |
| S55 | *Codonopsis pilosula* (Franch.) Nannf. | Gansu Province | 2 | 2019/11/16 |
| S56 | *Codonopsis pilosula* (Franch.) Nannf. | Gansu Province | 2 | 2019/11/16 |
| S57 | *Codonopsis pilosula* (Franch.) Nannf. | Gansu Province | 2 | 2019/11/22 |
| S58 | *Codonopsis pilosula* (Franch.) Nannf. | Gansu Province | 2 | 2019/11/23 |
| S59 | *Codonopsis pilosula* (Franch.) Nannf. | Gansu Province | 2 | 2019/11/24 |
| S60 | *Codonopsis pilosula* (Franch.) Nannf. | Gansu Province | 2 | 2019/11/25 |
| S61 | *Codonopsis pilosula* (Franch.) Nannf. | Gansu Province | 2 | 2019/11/25 |
| S62 | *Codonopsis pilosula* (Franch.) Nannf. | Gansu Province | 2 | 2019/11/25 |
| S63 | *Codonopsis pilosula* (Franch.) Nannf. | Gansu Province | 2 | 2019/11/13 |
| S64 | *Codonopsis pilosula* (Franch.) Nannf. | Gansu Province | 2 | 2019/11/13 |
| S65 | *Codonopsis pilosula* (Franch.) Nannf. | Gansu Province | 2 | 2019/11/13 |
| S66 | *Codonopsis pilosula* (Franch.) Nannf. | Gansu Province | 2 | 2019/11/13 |
| S67 | *Codonopsis pilosula* (Franch.) Nannf. | Gansu Province | 2 | 2019/11/13 |
| S68 | *Codonopsis pilosula* (Franch.) Nannf. | Gansu Province | 2 | 2019/11/13 |
| S69 | *Codonopsis pilosula* (Franch.) Nannf. | Gansu Province | 2 | 2019/11/13 |
| S70 | *Codonopsis pilosula* (Franch.) Nannf. | Gansu Province | 2 | 2019/11/13 |
| S71 | *Codonopsis pilosula* (Franch.) Nannf. | Gansu Province | 2 | 2019/11/13 |
| S72 | *Codonopsis pilosula* (Franch.) Nannf. | Gansu Province | 2 | 2019/11/13 |
| S73 | *Codonopsis pilosula* (Franch.) Nannf. | Gansu Province | 2 | 2019/11/13 |
| S74 | *Codonopsis pilosula* (Franch.) Nannf. | Gansu Province | 2 | 2019/11/13 |
| S75 | *Codonopsis pilosula* (Franch.) Nannf. | Gansu Province | 2 | 2019/11/13 |
| S76 | *Codonopsis pilosula* (Franch.) Nannf. | Gansu Province | 2 | 2019/11/13 |
| S77 | *Codonopsis pilosula* (Franch.) Nannf. | Gansu Province | 2 | 2019/11/13 |
| S78 | *Codonopsis pilosula* (Franch.) Nannf. | Gansu Province | 2 | 2019/11/13 |
| S79 | *Codonopsis pilosula* (Franch.) Nannf. | Gansu Province | 2 | 2019/11/13 |
| S80 | *Codonopsis pilosula* (Franch.) Nannf. | Gansu Province | 2 | 2019/11/13 |
| S81 | *Codonopsis pilosula* (Franch.) Nannf. | Gansu Province | 2 | 2019/11/13 |
| S82 | *Codonopsis pilosula* (Franch.) Nannf. | Gansu Province | 2 | 2019/11/23 |
| S83 | *Codonopsis pilosula* (Franch.) Nannf. | Gansu Province | 2 | 2019/11/23 |
| S84 | *Codonopsis pilosula* (Franch.) Nannf. | Gansu Province | 2 | 2019/11/23 |
| S85 | *Codonopsis pilosula* (Franch.) Nannf. | Gansu Province | 2 | 2019/11/23 |
| S86 | *Codonopsis pilosula* (Franch.) Nannf. | Gansu Province | 2 | 2019/11/23 |
| S87 | *Codonopsis pilosula* Nannf*.* var*. modesta*（Nannf. ）L. T. Shen | Gansu Province | 3 | 2019/11/13 |
| S88 | *Codonopsis pilosula* Nannf*.* var*. modesta*（Nannf. ）L. T. Shen | Gansu Province | 3 | 2019/11/17 |
| S89 | *Codonopsis pilosula* Nannf*.* var*. modesta*（Nannf. ）L. T. Shen | Gansu Province | 3 | 2019/11/15 |
| S90 | *Codonopsis pilosula* Nannf*.* var*. modesta*（Nannf. ）L. T. Shen | Gansu Province | 6 | 2019/11/20 |
| S91 | *Codonopsis pilosula* Nannf*.* var*. modesta*（Nannf. ）L. T. Shen | Gansu Province | 4 | 2019/11/20 |
| S92 | *Codonopsis pilosula* Nannf*.* var*. modesta*（Nannf. ）L. T. Shen | Gansu Province | 4 | 2019/11/18 |
| S93 | *Codonopsis pilosula* Nannf*.* var*. modesta*（Nannf. ）L. T. Shen | Gansu Province | 4 | 2019/11/20 |
| S94 | *Codonopsis pilosula* Nannf*.* var*. modesta*（Nannf. ）L. T. Shen | Gansu Province | 4 | 2019/11/20 |
| S95 | *Codonopsis pilosula* Nannf*.* var*. modesta*（Nannf. ）L. T. Shen | Gansu Province | 2 | 2019/11/30 |
| S96 | *Codonopsis pilosula* Nannf*.* var*. modesta*（Nannf. ）L. T. Shen | Gansu Province | 4 | 2019/11/30 |
| S97 | *Codonopsis pilosula* Nannf*.* var*. modesta*（Nannf. ）L. T. Shen | Gansu Province | 3 | 2019/12/1 |
| S98 | *Codonopsis pilosula* Nannf*.* var*. modesta*（Nannf. ）L. T. Shen | Gansu Province | 3 | 2019/12/3 |
| S99 | *Codonopsis pilosula* Nannf*.* var*. modesta*（Nannf. ）L. T. Shen | Gansu Province | 3 | 2019/12/3 |
| S100 | *Codonopsis pilosula* Nannf*.* var*. modesta*（Nannf. ）L. T. Shen | Gansu Province | 3 | 2019/12/3 |
| S101 | *Codonopsis pilosula* Nannf*.* var*. modesta*（Nannf. ）L. T. Shen | Gansu Province | 3 | 2019/12/3 |
| S102 | *Codonopsis pilosula* Nannf*.* var*. modesta*（Nannf. ）L. T. Shen | Gansu Province | 3 | 2019/12/3 |
| S103 | *Codonopsis pilosula* Nannf*.* var*. modesta*（Nannf. ）L. T. Shen | Gansu Province | 3 | 2019/11/12 |
| S104 | *Codonopsis pilosula* Nannf*.* var*. modesta*（Nannf. ）L. T. Shen | Gansu Province | 3 | 2019/11/12 |
| S105 | *Codonopsis pilosula* Nannf*.* var*. modesta*（Nannf. ）L. T. Shen | Gansu Province | 2 | 2019/11/12 |
| S106 | *Codonopsis pilosula* Nannf*.* var*. modesta*（Nannf. ）L. T. Shen | Gansu Province | 3 | 2019/11/12 |
| S107 | *Codonopsis pilosula* Nannf*.* var*. modesta*（Nannf. ）L. T. Shen | Gansu Province | 2 | 2019/11/15 |
| S108 | *Codonopsis pilosula* Nannf*.* var*. modesta*（Nannf. ）L. T. Shen | Gansu Province | 2 | 2019/11/18 |
| S109 | *Codonopsis pilosula* Nannf*.* var*. modesta*（Nannf. ）L. T. Shen | Gansu Province | 2 | 2019/11/11 |
| S110 | *Codonopsis pilosula* (Franch.) Nannf. | Gansu Province | - | 2019/11/8 |
| S111 | *Codonopsis pilosula* (Franch.) Nannf. | Gansu Province | - | 2019/11/8 |
| S112 | *Codonopsis pilosula* (Franch.) Nannf. | Gansu Province | - | 2019/11/8 |
| S113 | *Codonopsis pilosula* (Franch.) Nannf. | Gansu Province | - | 2019/12/10 |
| S114 | *Codonopsis pilosula* (Franch.) Nannf. | Gansu Province | - | 2019/12/10 |
| S115 | *Codonopsis pilosula* (Franch.) Nannf. | Gansu Province | - | 2019/11/9 |
| S116 | *Codonopsis pilosula* (Franch.) Nannf. | Gansu Province | - | 2019/11/9 |
| S117 | *Codonopsis pilosula* (Franch.) Nannf. | Gansu Province | - | 2019/11/9 |
| S118 | *Codonopsis pilosula* (Franch.) Nannf. | Gansu Province | 2 | 2019/11/10 |
| S119 | *Codonopsis pilosula* (Franch.) Nannf. | Gansu Province | 2 | 2019/10/25 |
| S120 | *Codonopsis pilosula* (Franch.) Nannf. | Gansu Province | 2 | 2019/11/15 |
| S121 | *Codonopsis pilosula* (Franch.) Nannf. | Gansu Province | 3 | 2019/10/18 |
| S122 | *Codonopsis pilosula* (Franch.) Nannf. | Gansu Province | 2 | 2020/3/9 |
| S123 | *Codonopsis pilosula* (Franch.) Nannf. | Gansu Province | 2 | 2019/10/20 |
| S124 | *Codonopsis pilosula* (Franch.) Nannf. | Gansu Province | 2 | 2019/10/19 |
| S125 | *Codonopsis pilosula* (Franch.) Nannf. | Gansu Province | 2 | 2019/10/16 |
| S126 | *Codonopsis pilosula* (Franch.) Nannf. | Gansu Province | 2 | 2019/10/15 |
| S127 | *Codonopsis pilosula* (Franch.) Nannf. | Gansu Province | 2 | 2019/10/20 |
| S128 | *Codonopsis pilosula* (Franch.) Nannf. | Gansu Province | 2 | 2019/10/15 |
| S129 | *Codonopsis pilosula* (Franch.) Nannf. | Gansu Province | 2 | 2019/9/25 |
| S130 | *Codonopsis pilosula* (Franch.) Nannf. | Gansu Province | 2 | 2019/10/20 |
| S131 | *Codonopsis pilosula* (Franch.) Nannf. | Gansu Province | 2 | 2019/10/21 |
| S132 | *Codonopsis pilosula* (Franch.) Nannf. | Gansu Province | 2 | 2019/10/20 |
| S133 | *Codonopsis pilosula* (Franch.) Nannf. | Gansu Province | 2 | 2019 |
| S134 | *Codonopsis pilosula* (Franch.) Nannf. | Gansu Province | 2 | 2019 |
| S135 | *Codonopsis pilosula* (Franch.) Nannf. | Gansu Province | 2 | 2019 |
| S136 | *Codonopsis pilosula* (Franch.) Nannf. | Gansu Province | 2 | 2019 |
| S137 | *Codonopsis pilosula* (Franch.) Nannf. | Shanxi Province | 2 | 2019/10/19 |
| S138 | *Codonopsis pilosula* (Franch.) Nannf. | Shanxi Province | 2 | 2019/10/25 |
| S139 | *Codonopsis pilosula* (Franch.) Nannf. | Shanxi Province | 2 | 2019/10/14 |
| S140 | *Codonopsis pilosula* (Franch.) Nannf. | Shanxi Province | 2 | 2020/3/25 |
| S141 | *Codonopsis pilosula* (Franch.) Nannf. | Shanxi Province | 10 | 2020/3/25 |
| S142 | *Codonopsis pilosula* (Franch.) Nannf. | Shanxi Province | 2 | 2020/5/5 |
| S143 | *Codonopsis pilosula* (Franch.) Nannf. | Shanxi Province | 2 | 2020/5/5 |
| S144 | *Codonopsis pilosula* (Franch.) Nannf. | Shanxi Province | 2 | 2020/5/5 |
| S145 | *Codonopsis pilosula* (Franch.) Nannf. | Shanxi Province | 2 | 2020/5/5 |
| S146 | *Codonopsis pilosula* (Franch.) Nannf. | Shanxi Province | 2 | 2020/5/5 |
| S147 | *Codonopsis pilosula* (Franch.) Nannf. | Shanxi Province | 2 | 2020/5/5 |
| S148 | *Codonopsis tangshen* Oliv*.* | Hubei Province | 4 | 2020/8/10 |
| S149 | *Codonopsis tangshen* Oliv*.* | Hubei Province | 3 | 2020/8/10 |
| S150 | *Codonopsis tangshen* Oliv*.* | Hubei Province | 3 | 2020/8/10 |
| S151 | *Codonopsis tangshen* Oliv*.* | Hubei Province | 2 | 2020/8/10 |
| S152 | *Codonopsis tangshen* Oliv*.* | Hubei Province | 3 | 2020/8/10 |
| S153 | *Codonopsis tangshen* Oliv*.* | Hubei Province | 4 | 2020/8/10 |
| S154 | *Codonopsis tangshen* Oliv*.* | Hubei Province | - | 2020/8/10 |
| S155 | *Codonopsis tangshen* Oliv*.* | Hubei Province | - | 2020/8/10 |
| S156 | *Codonopsis tangshen* Oliv*.* | Hubei Province | - | 2020/8/10 |
| S157 | *Codonopsis tangshen* Oliv*.* | Chongqing Municipality | 3 | 2020/8/11 |
| S158 | *Codonopsis tangshen* Oliv*.* | Chongqing Municipality | 4 | 2020/8/11 |
| S159 | *Codonopsis tangshen* Oliv*.* | Chongqing Municipality | 2 | 2020/8/11 |
| S160 | *Codonopsis tangshen* Oliv*.* | Chongqing Municipality | - | 2020/8/11 |
| S161 | *Codonopsis tangshen* Oliv*.* | Guizhou Province | 2.4 | 2020/8/13 |
| S162 | *Codonopsis tangshen* Oliv*.* | Guizhou Province | 6 | 2020/8/13 |
| S163 | *Codonopsis tangshen* Oliv*.* | Guizhou Province | 4 | 2020/8/13 |
| S164 | *Codonopsis tangshen* Oliv*.* | Guizhou Province | - | 2020/8/13 |


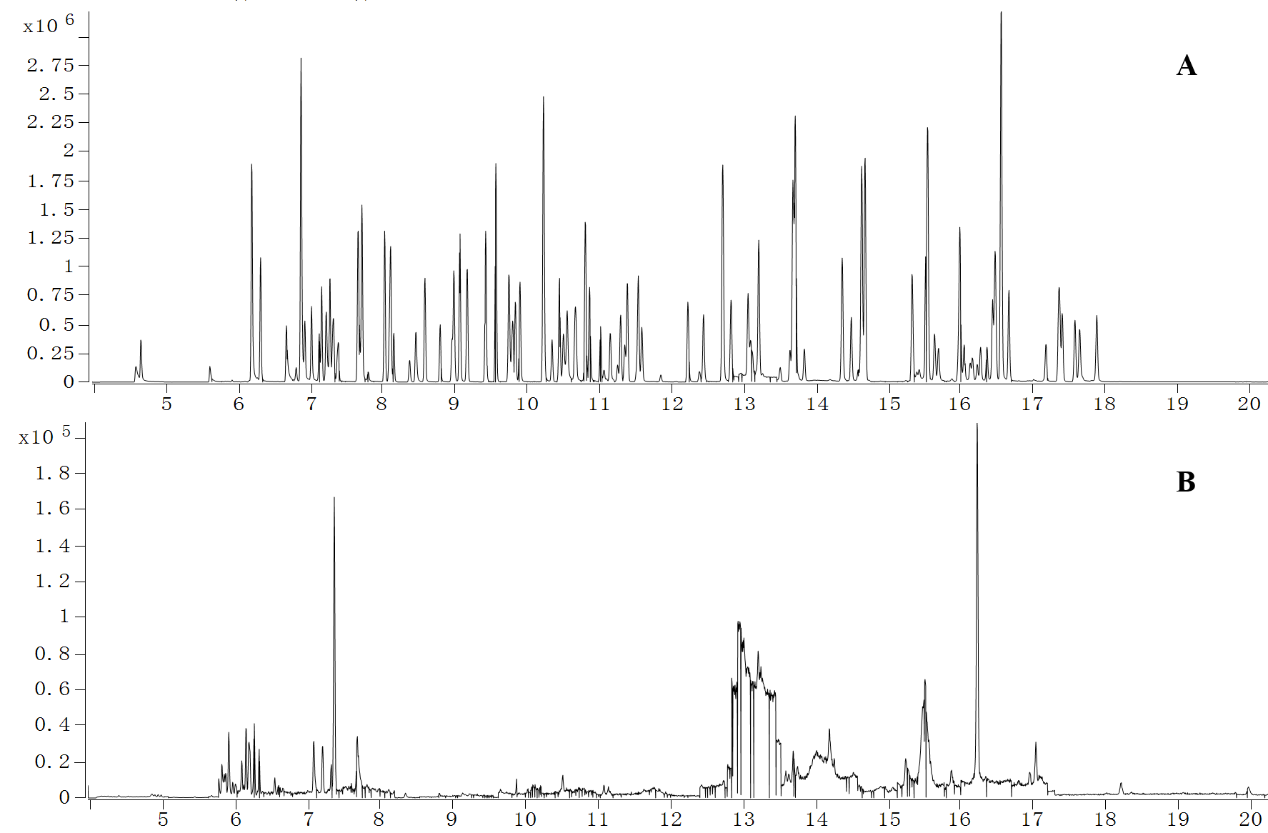


**Figure. S1** GC-MS/MS total ion flow diagrams for standard (A) and sample (B)


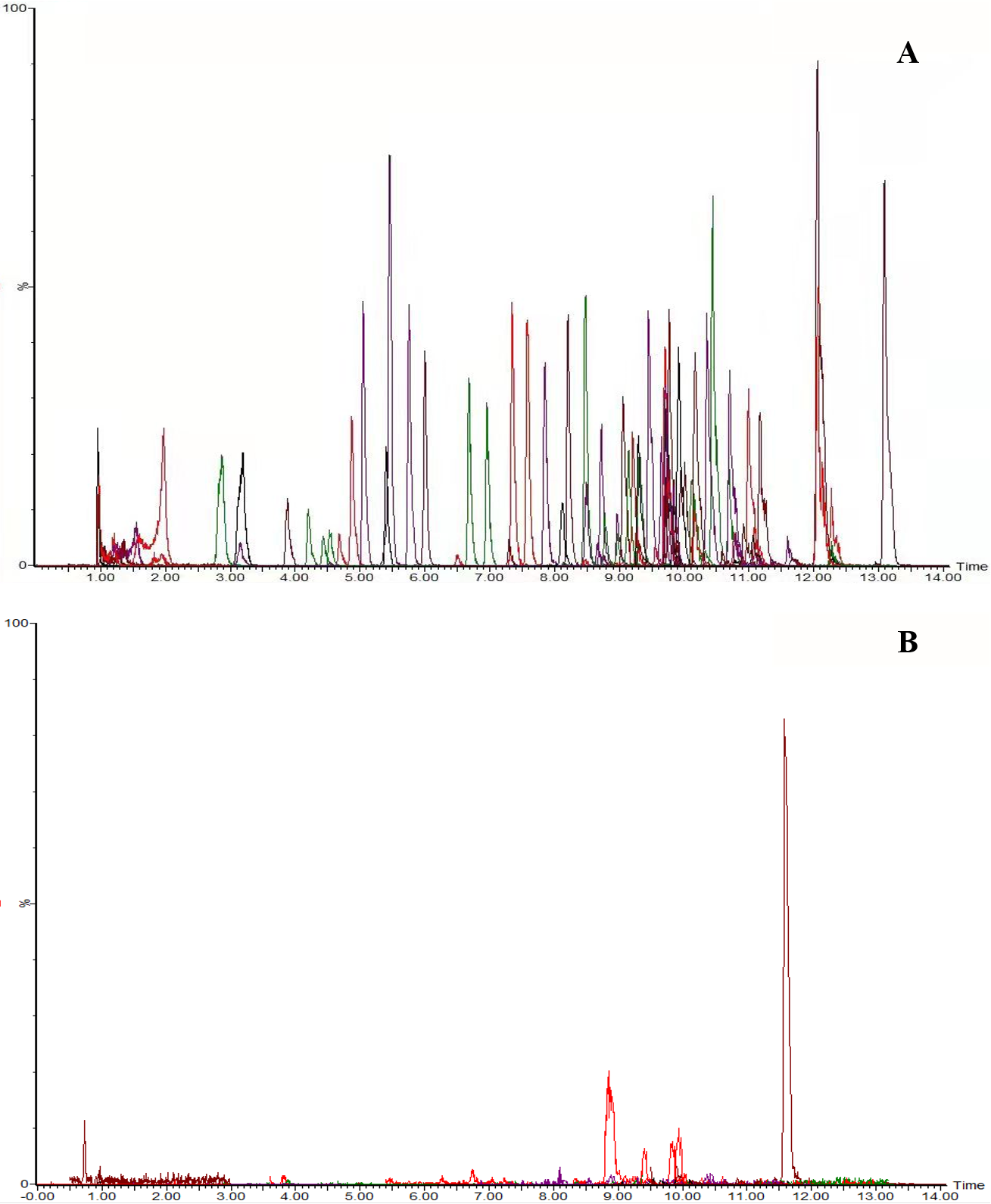


**Figure. S2** LC-MS/MS total ion flow diagrams for standard (A) and sample (B)

**A**

Methamidophos(**1**) Dichlorvos(**2**) Acephate(**3**) O-Phenylphenol(**4**) Molinate(**5**) Omethoate(**6**)

Tecnazene(**7**) Dphenylamine(**8**) Ethoprophos(**9**) Chlorpropham(**10**) Chlordimeform(**11**) Bifenazate(**12**)

Trifluralin(**13**) Monocrotophos(**14**) Sulfotep(**15**) Phorate(**16**) Cadusafos(**17**) Hexachlorobenzene(**18**)

Dicloran(**19**) Dimethoate(**20**) Carbofuran(**21**) Dimethipin(**22**) Terbufos(**23**) Quintozene(**24**)

Fonofos(**25**) Diazinon(**26**) δ-HCH(**27**) Chlorothalonil(**28**) Pirimicarb(**29**) Phosphamidon(**30**)

Vinclozolin(**31**) Parathion-methyl(**32**) chlorpyrifos-methyl(**33**) Tolclofos-methyl(**34**) Heptachlor(**35**) Metalaxyl(**36**)

Fenitrothion(**37**) Pirimiphos-methyl(**38**) Malathion(**39**) Fenthion(**40**) Aldrin(**41**) Dursban(**42**)

Parathion(**43**) Triadimefon(**44**) Isocarbofos(**45**) Cyprodinil(**46**) Isofenphos-methyl(**47**) Pendimethalin(**48**)

Probenazole(**49**) Toluenfluorosulfonamide(**50**) Fipronil(**51**) Heptachlor epoxide(**52**) Triadimenol(**53**) Procymidone(**54**)

Triflumizole(**55**) Methidathion(**56**) Chlordan(**57**) Fenoxaprop-p-ethyl(**58**) Flumetralin(**59**) Endosulfan(**60**)

Fenamiphos(**61**) Hexaconazole(**62**) Isoprothiolane(**63**) Profenofos(**64**) Pretilachlor(**65**) Endrin(**66**)

Dieldrin(**67**) Myclobutanil(**68**) Flusilazole(**69**) Chlorfenapyr(**70**) Oxadixyl(**71**) Clethodim(**72**)

Triazophos(**73**) Ediphenghos(**74**) p,p'-DDT(**75**) Tebuconazole(**76**) Propargite(**77**) Biobenzfurethrin(**78**)

Iprodione(**79**) Carbosulfan(**80**) Phosmet(**81**) Bromopropylate(**82**) Bifenthrin(**83**) Fenpropathrin(**84**)

Phosalone(**85**) Cyfluthrin and Beta-cyfluthrin(**86**) Dechlorane(**87**) Biphentriazol(**88**) Cyfluthrin and beta-cyfluthrin(**89**) Pyridaben(**90**)

Coumaphos(**91**) Prochloraz(**92**) Permethrin(**93**) Fenbuconazole(**94**) Cypermethrin and beta-cypermethrin(**95**) Ethofenprox(**96**)

Fenvalerate(**97**) Fenvalerate and S-fenvalerate(**98**) Fluvalinate(**99**) Difenoconazole(**100**) Deltamethrin(**101**)

B


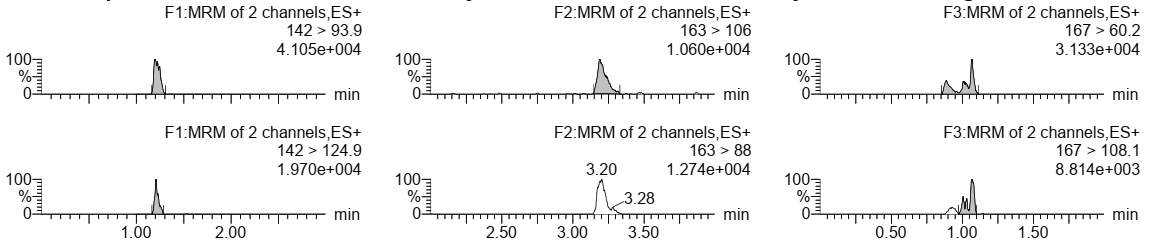

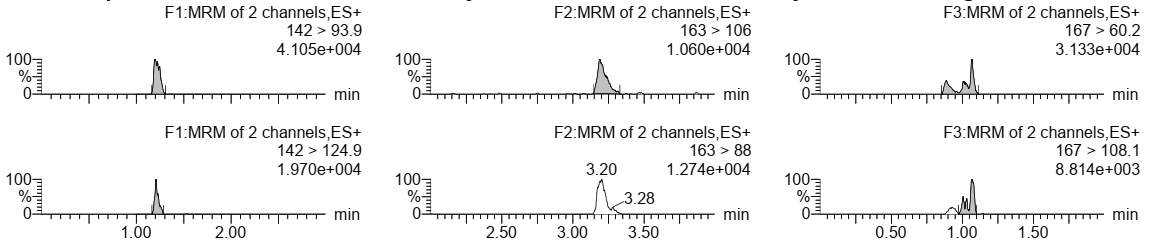

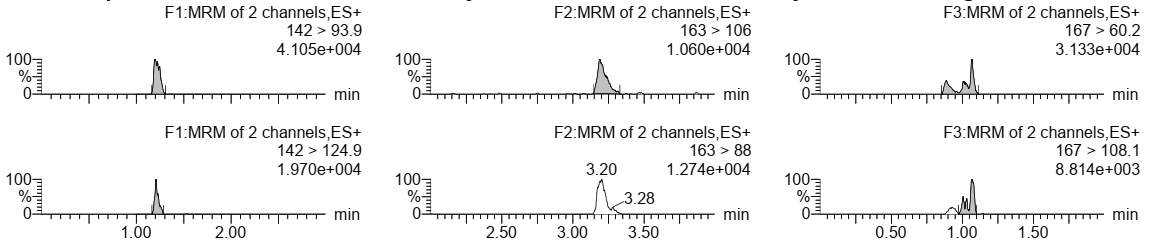


Methamidophos(**1**) Methomyl(**2**) Cyromazine(**3**)


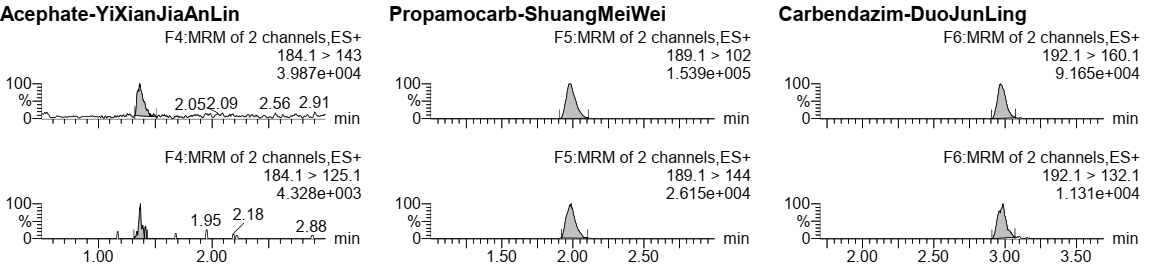

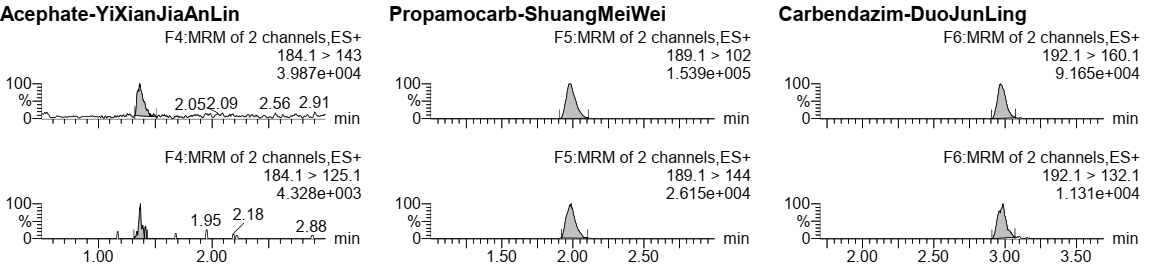

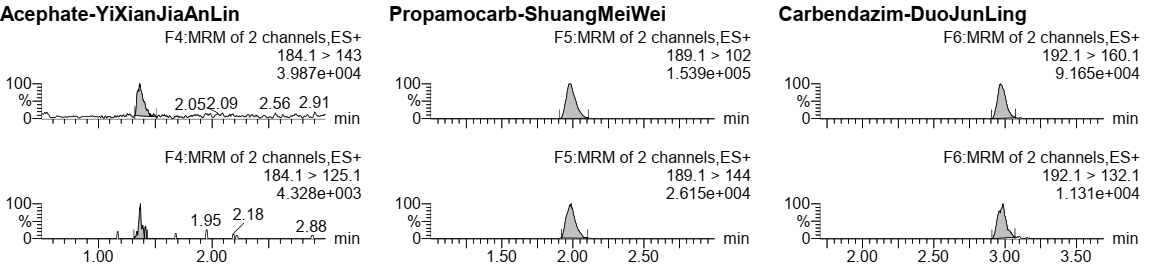


Acephate (**4**) Propamocarb (**5**) Carbendazim (**6**)


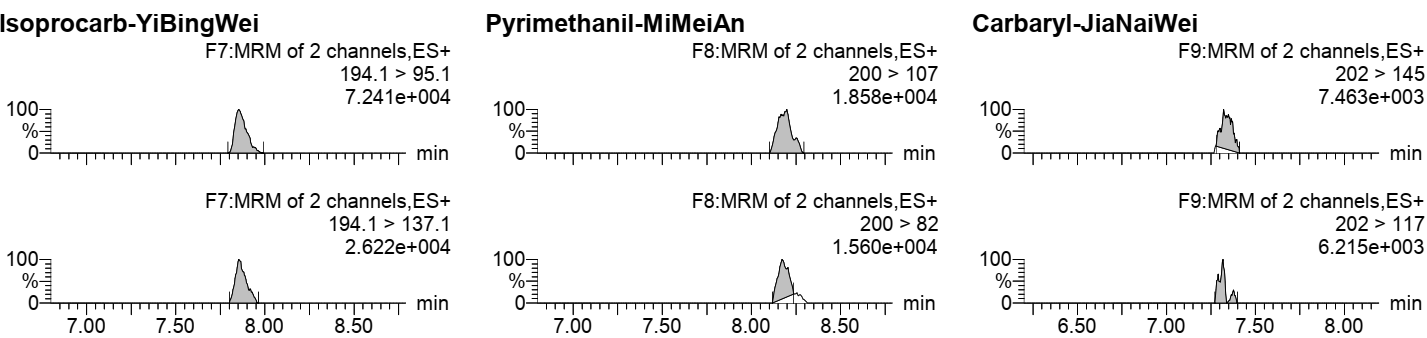

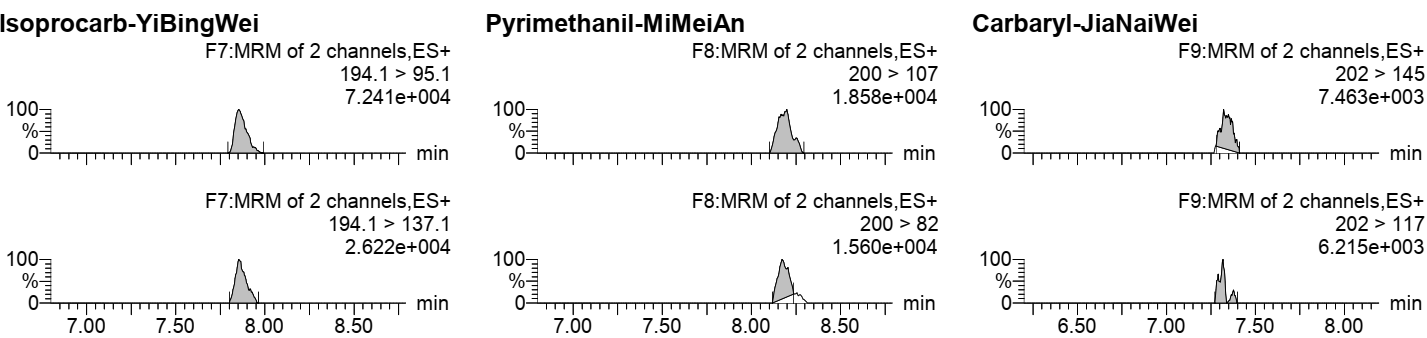

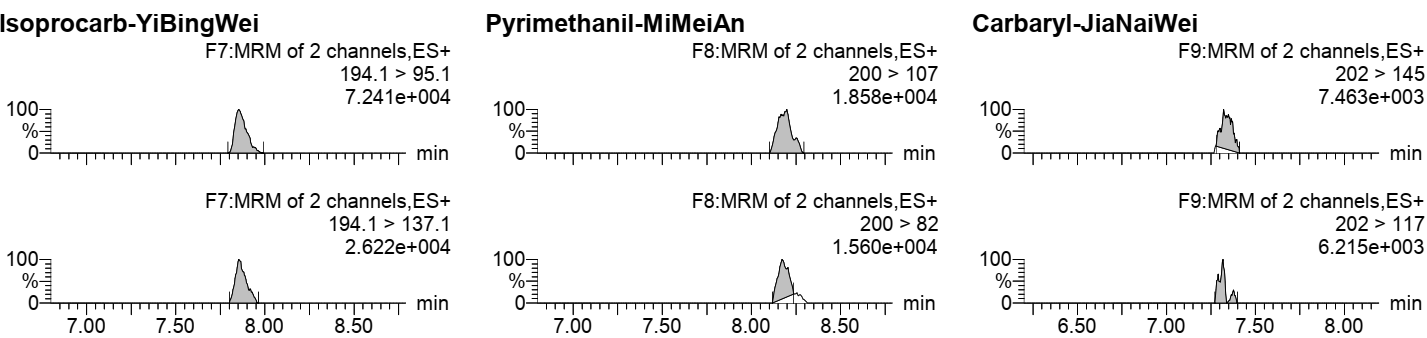


Isoprocarb (**7**) Pyrimethanil (**8**) Carbary (**9**)


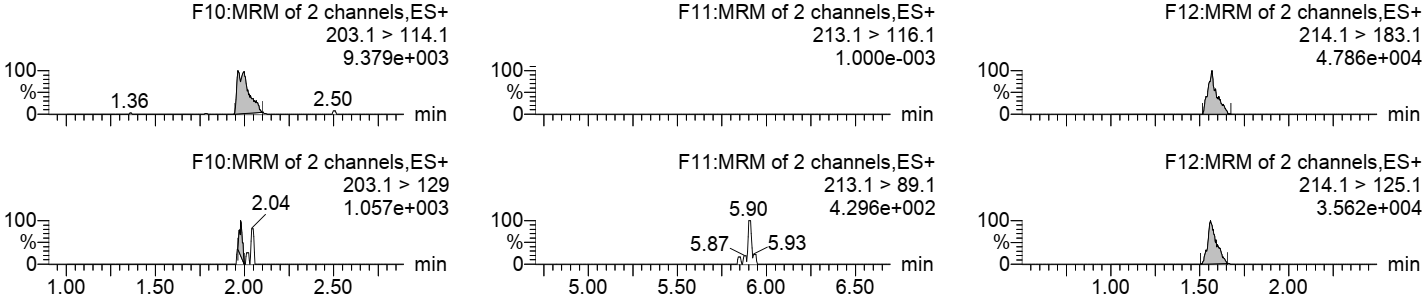

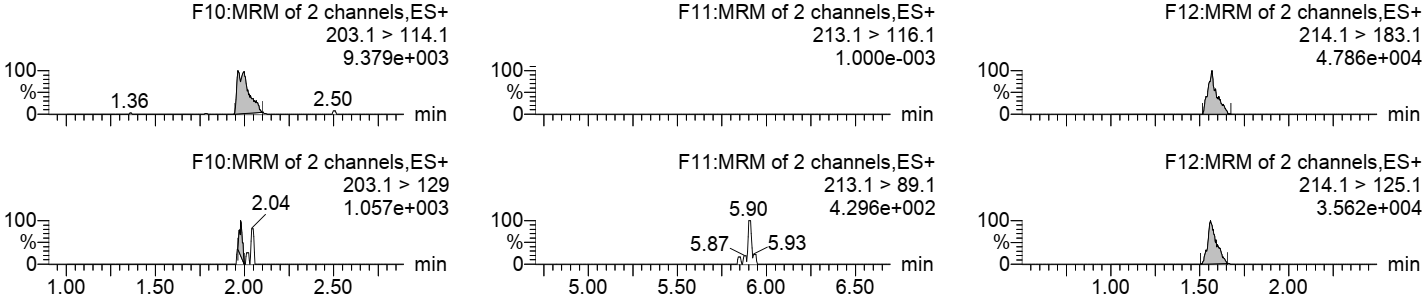

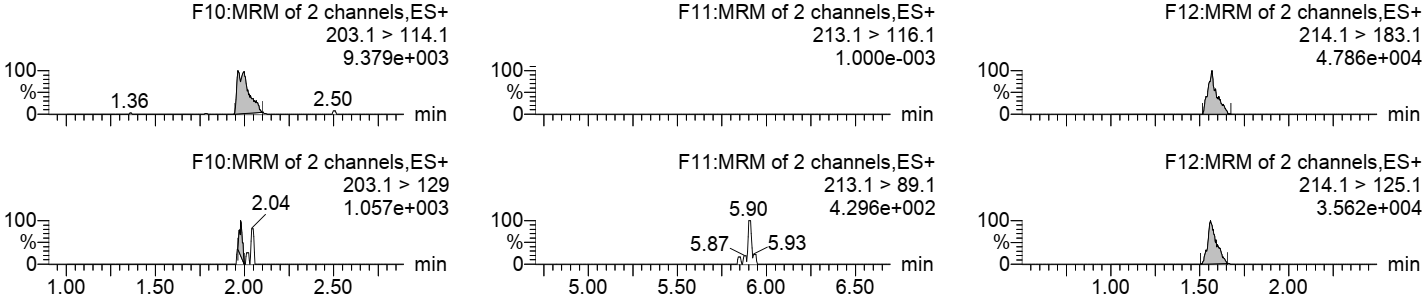


FuChongAn (**10**) Aldicarb (**11**) Omethoate (**12**)


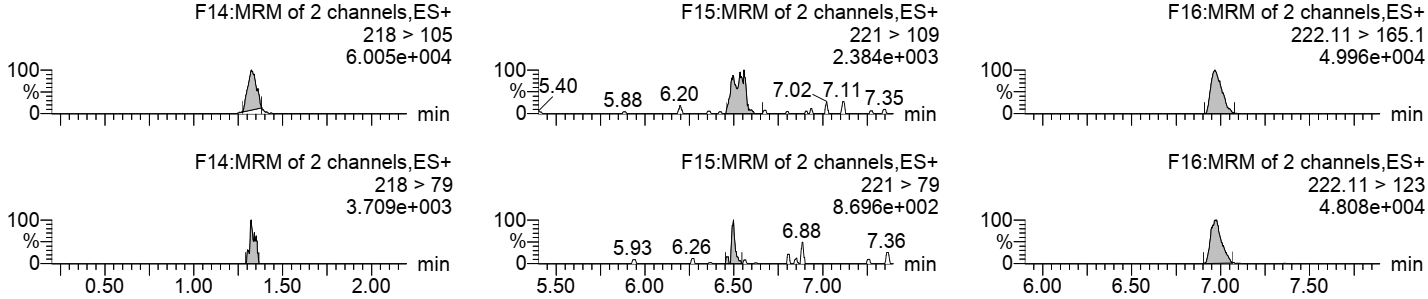

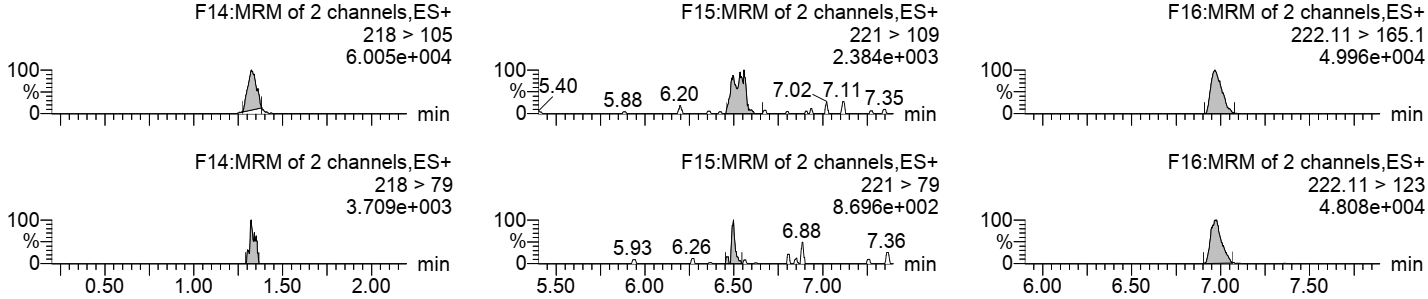

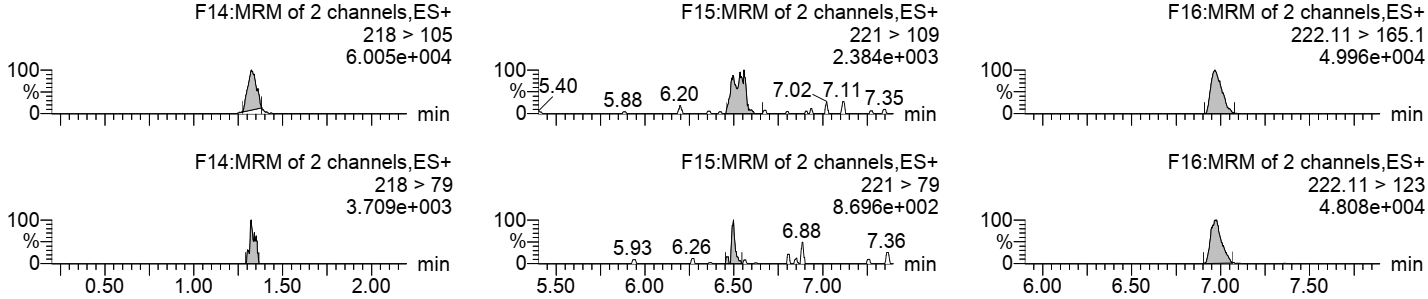


Pymetrozine (**13**) Dichlorvos (**14**) Carbofuran (**15**)


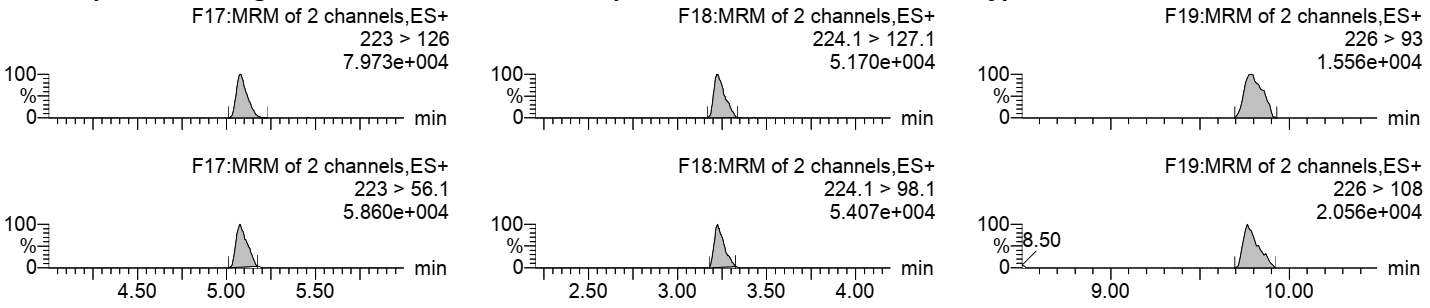

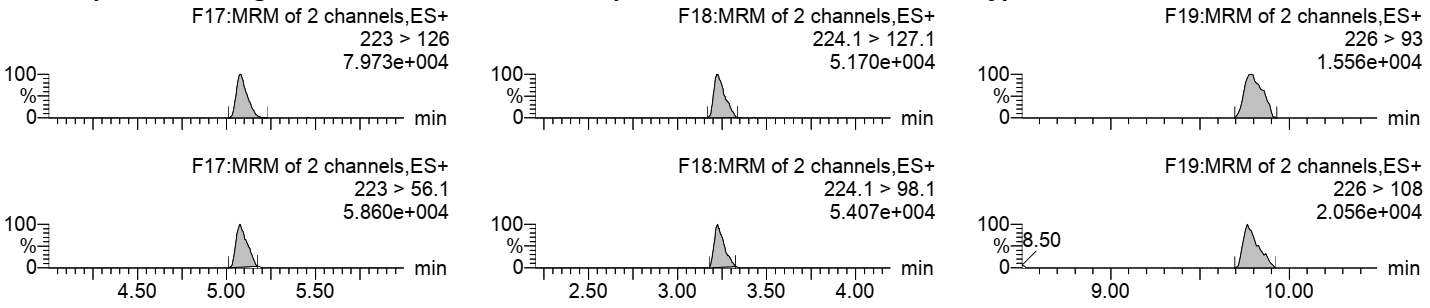

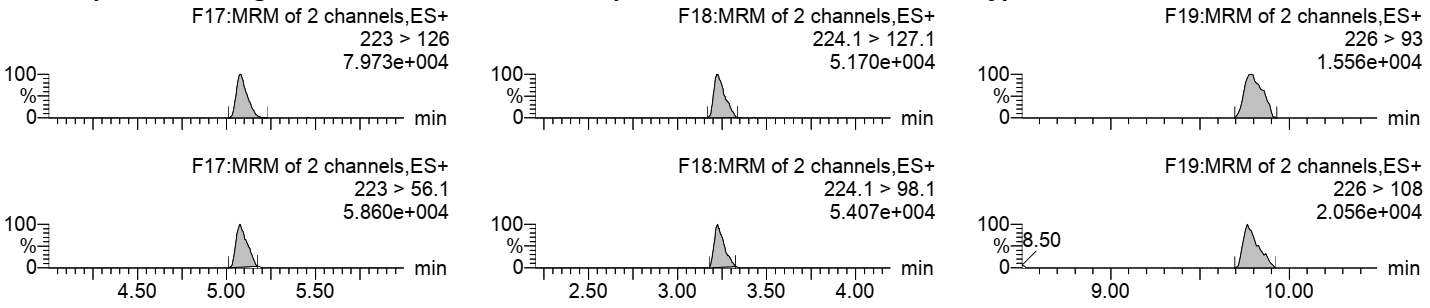


Acetamiprid (**16**) Monocrotophos (**17**) Cyprodinil (**18**)


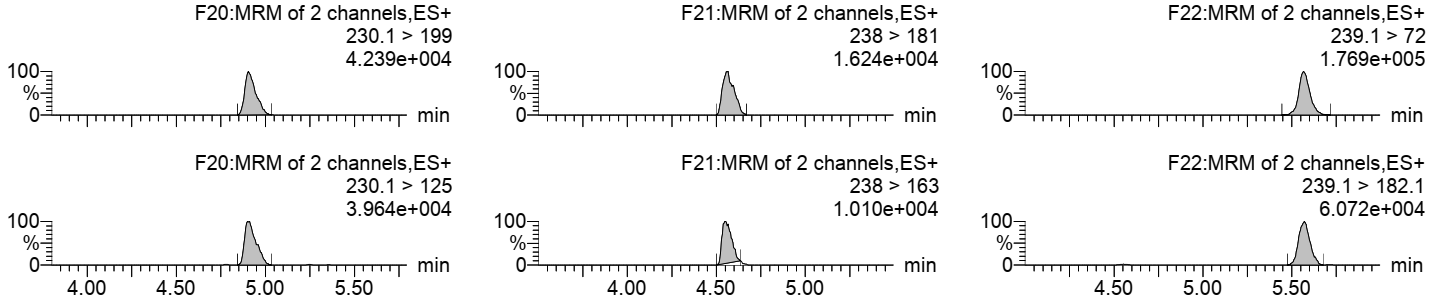

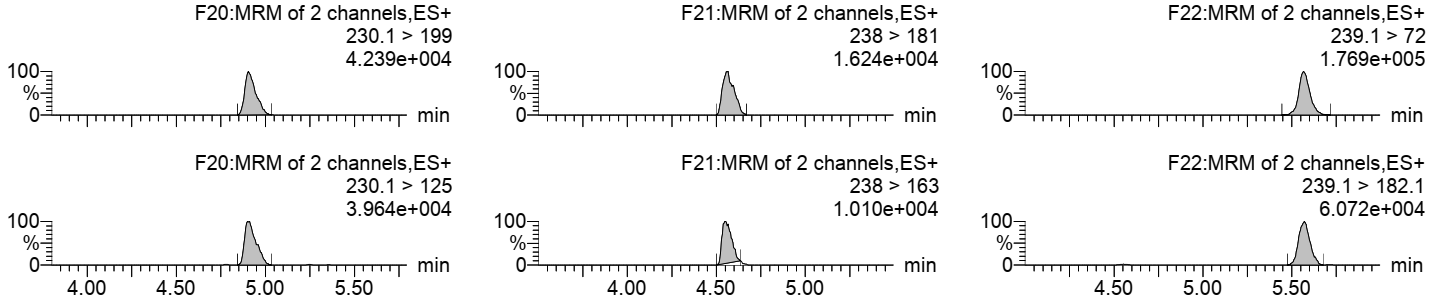

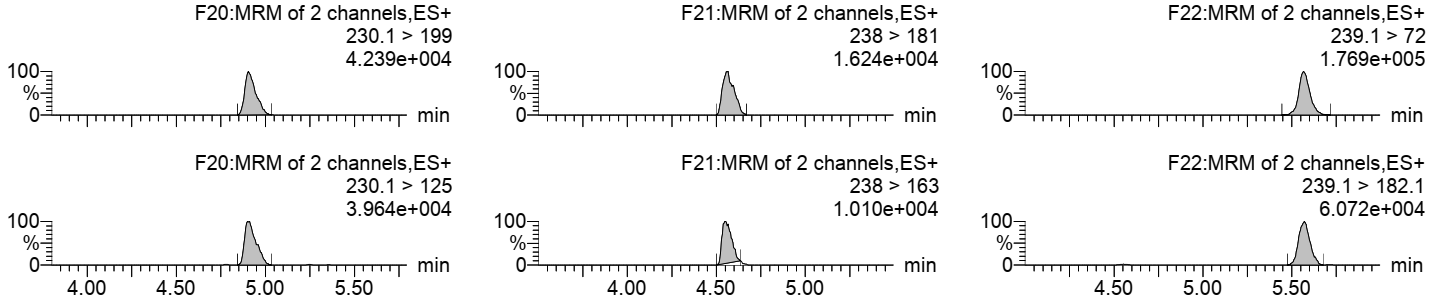


Dimethoate (**19**) Carbofuran-3-hydroxy (**20**) Pirimicarb (**21**)


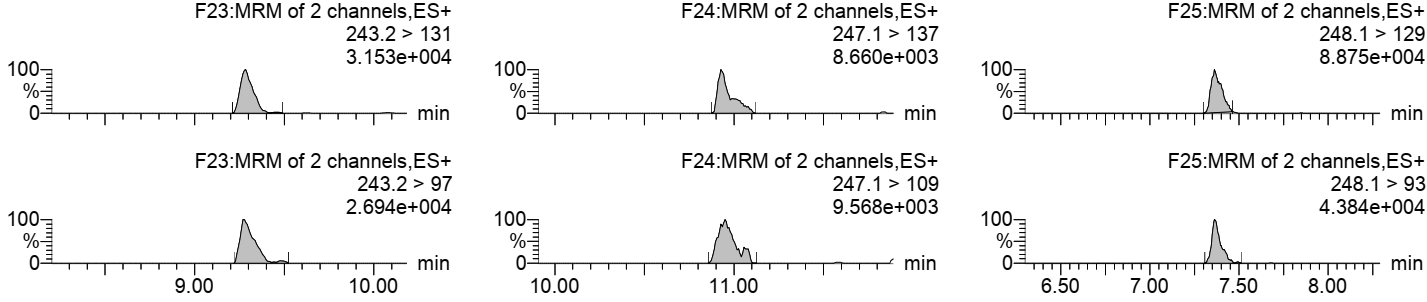

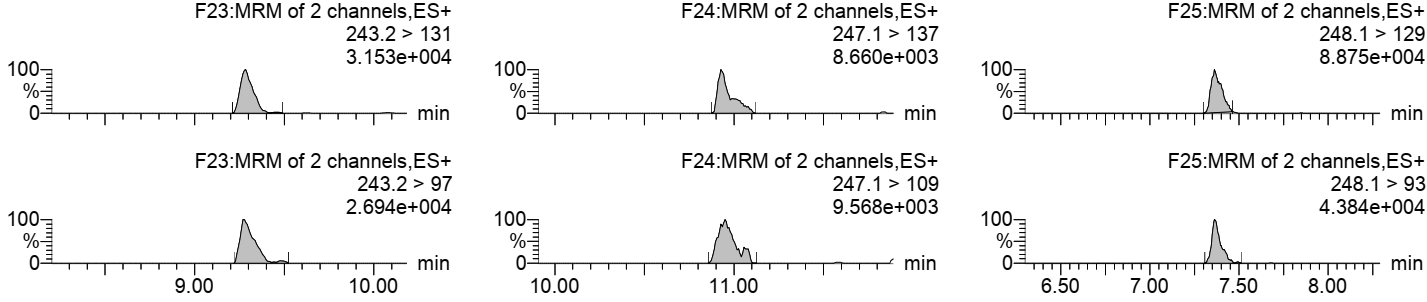

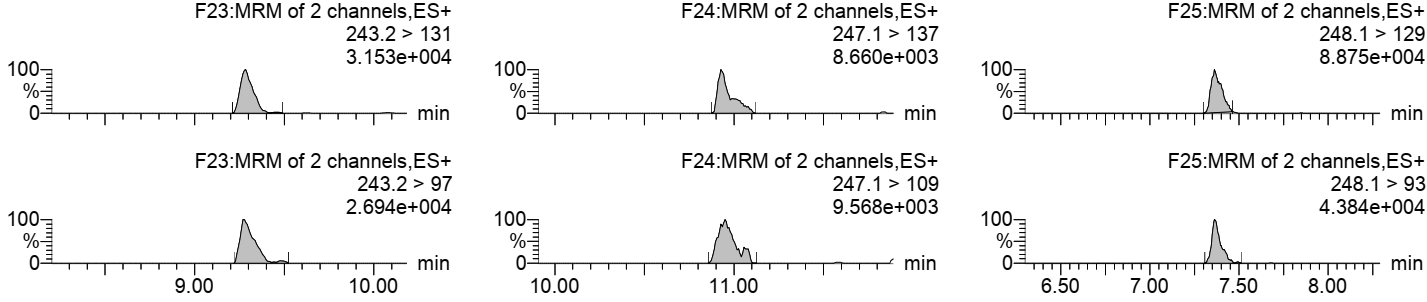


Ethoprophos (**22**) Fonofos (**23**) Forchlorfenuron (**24**)


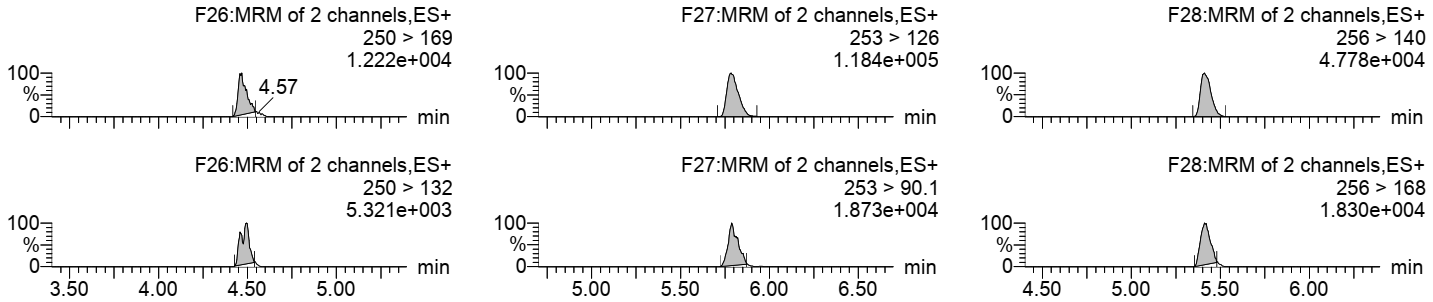

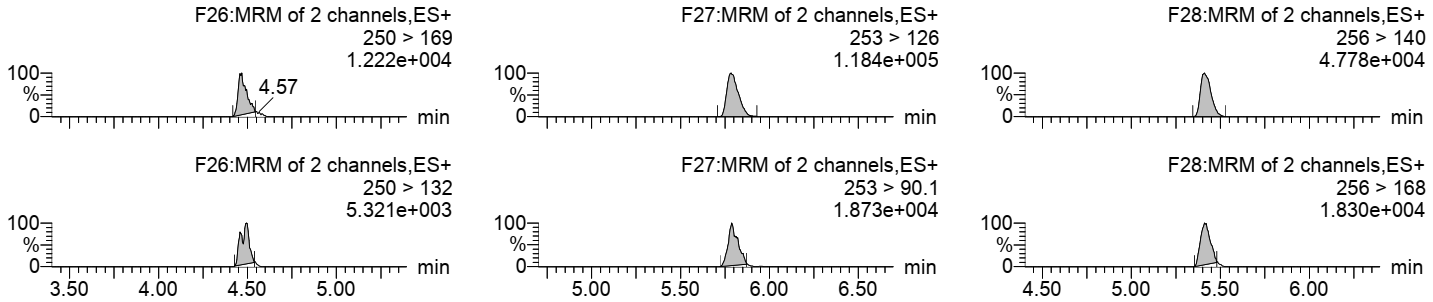

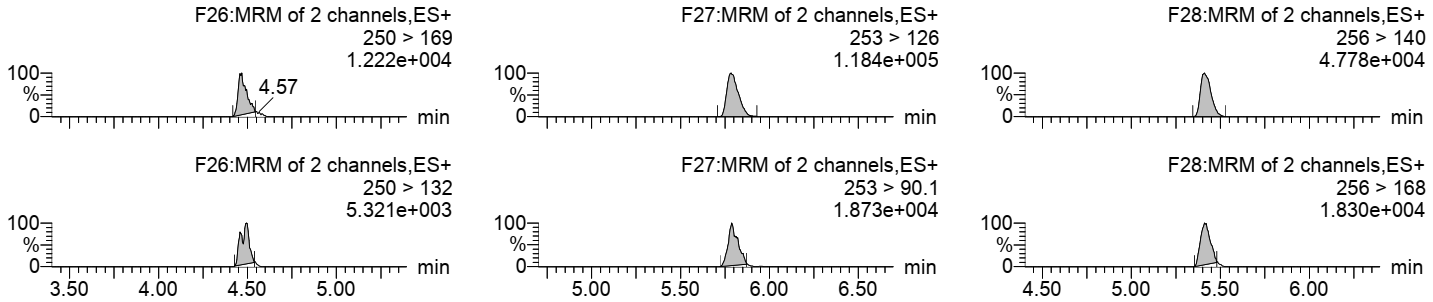


Clothianidin (**25**) Thiacloprid (**26**) Phosfolan (**27**)


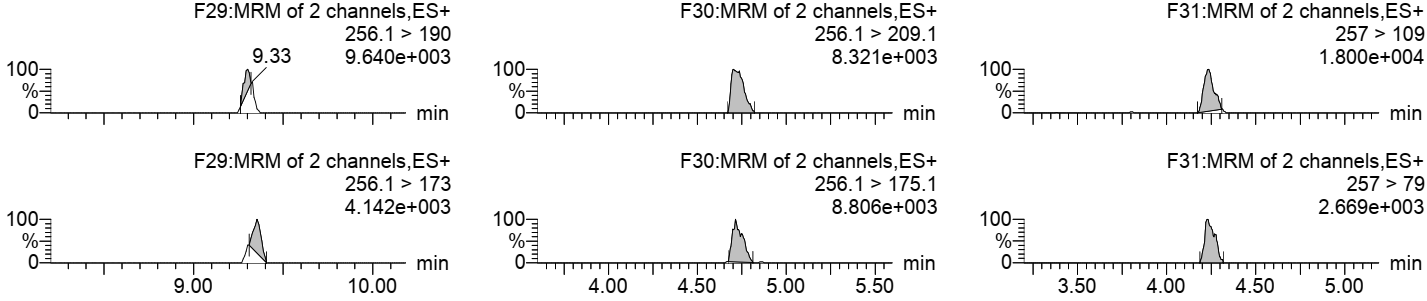

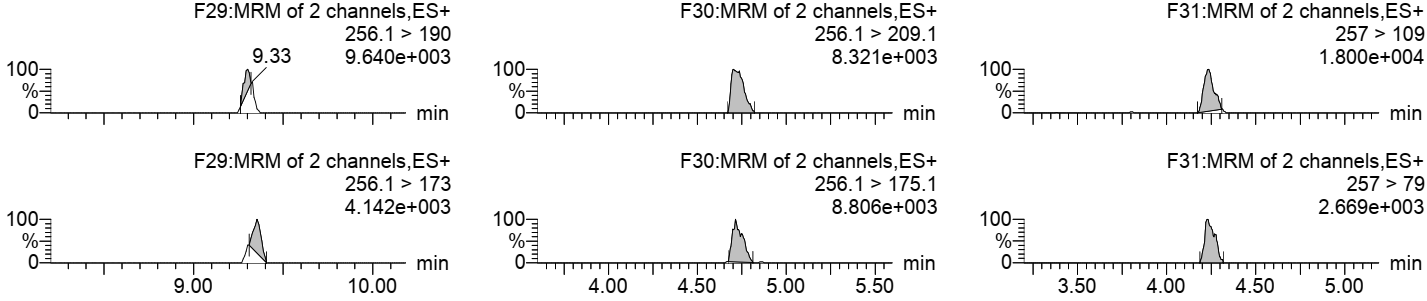

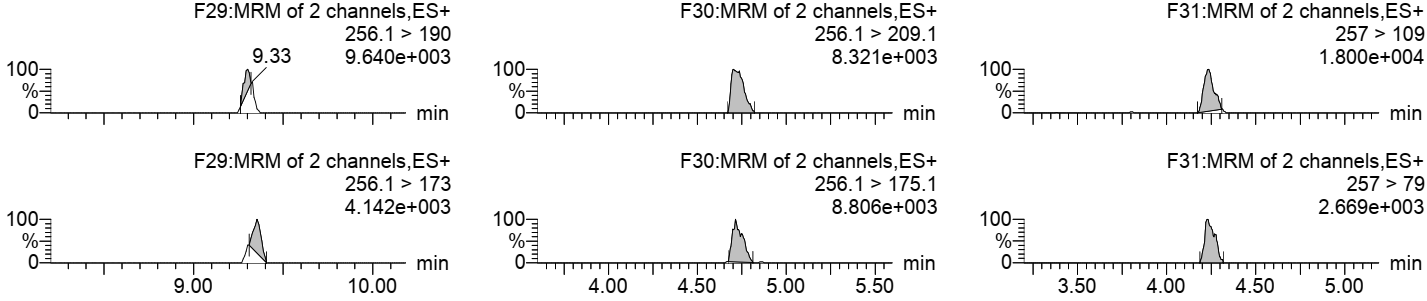


Propyzamide (**28**) Imidacloprid (**29**) Trichlorfon (**30**)


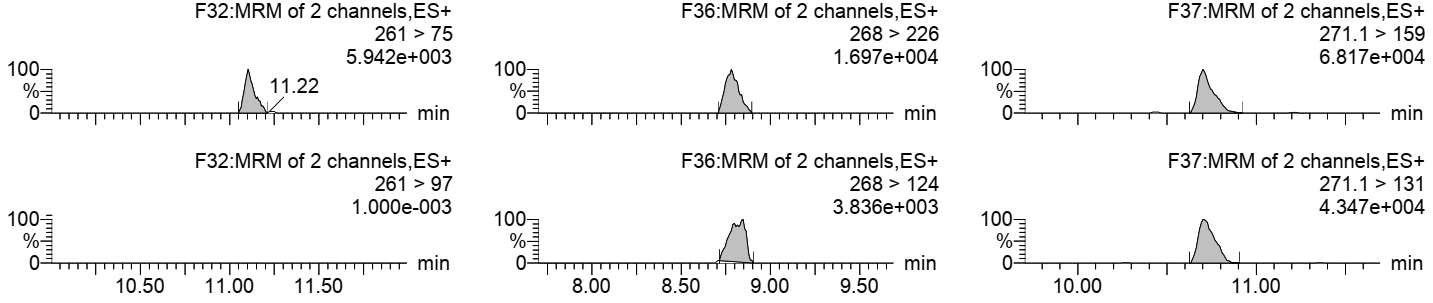

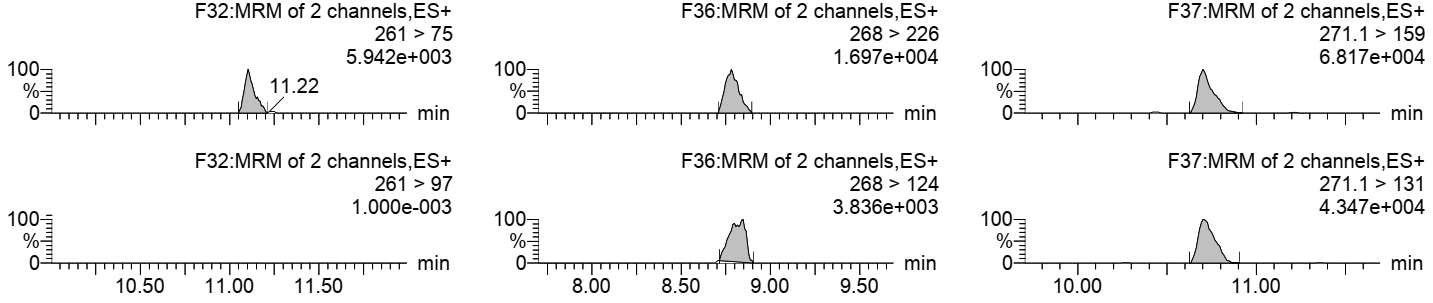

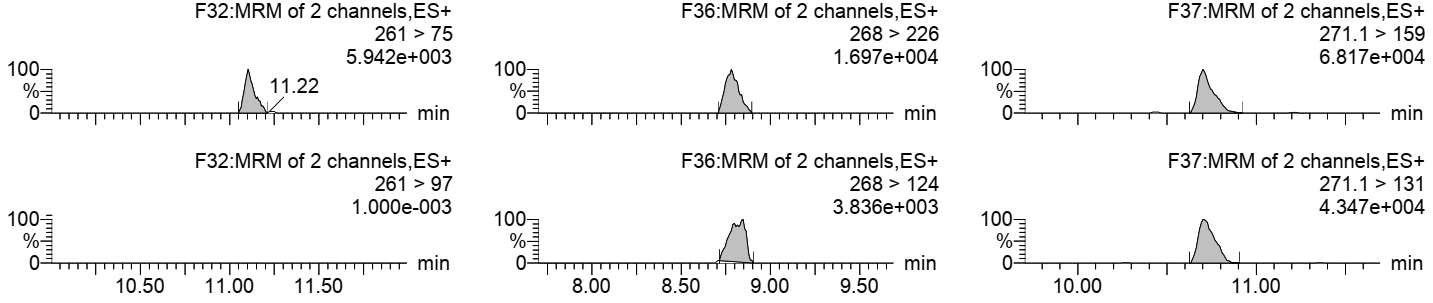


Phorate (**31**) Diethofencarb (**32**) Cadusafos (**33**)


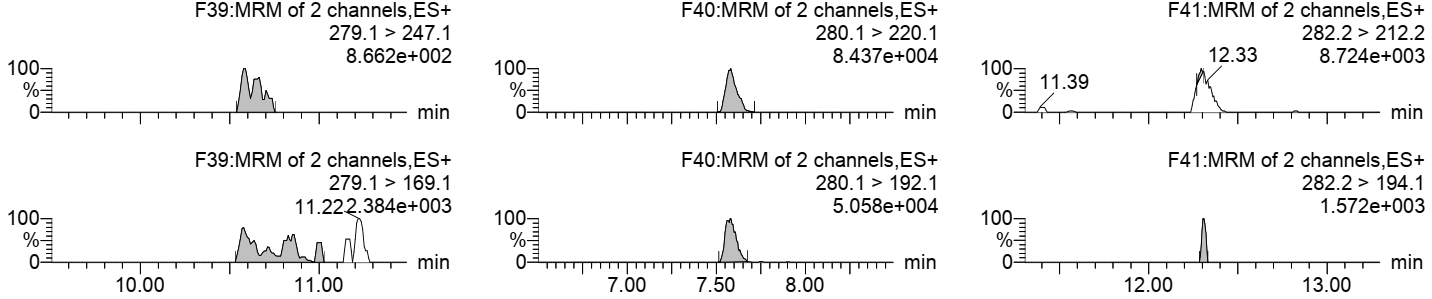

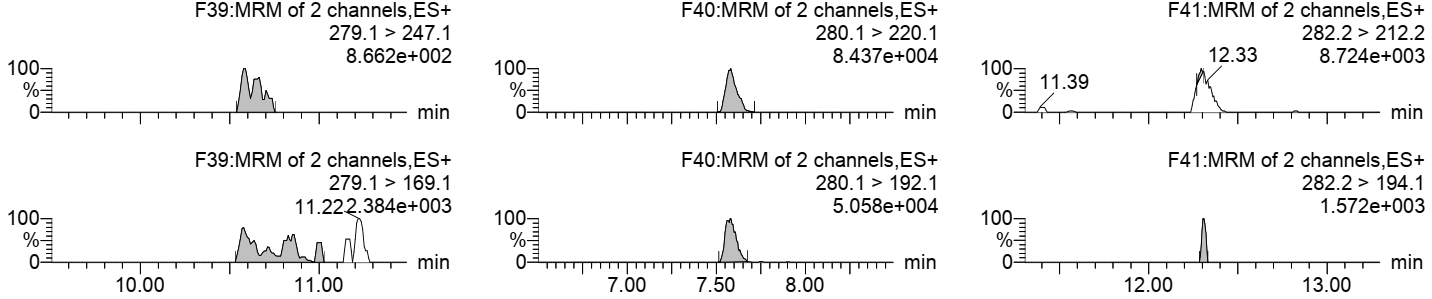

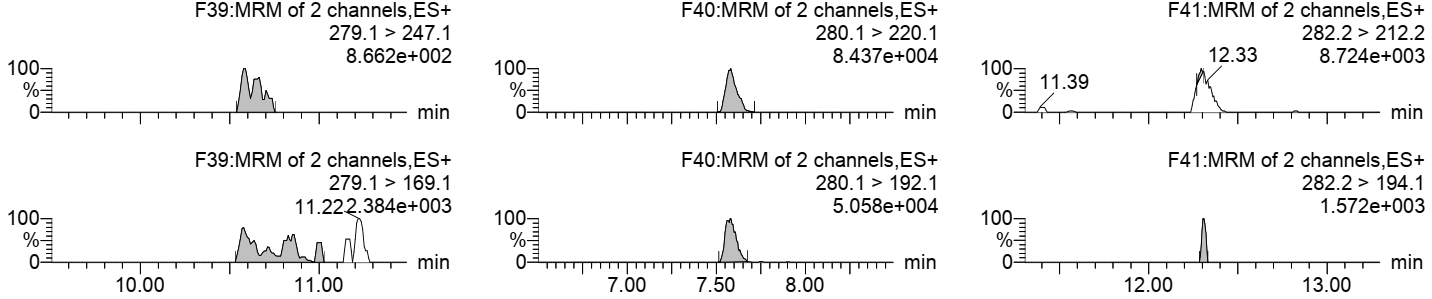


Fenthion (**34**) Metalaxyl (**35**) Pendimethalin (**36**)


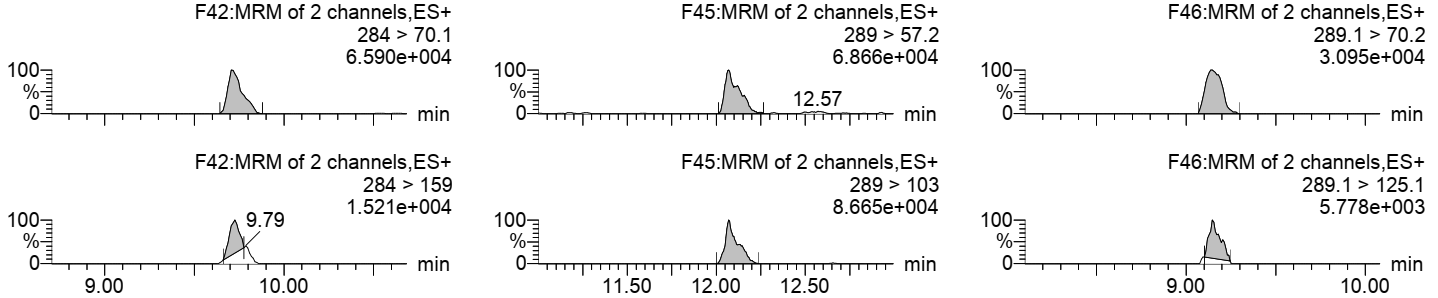

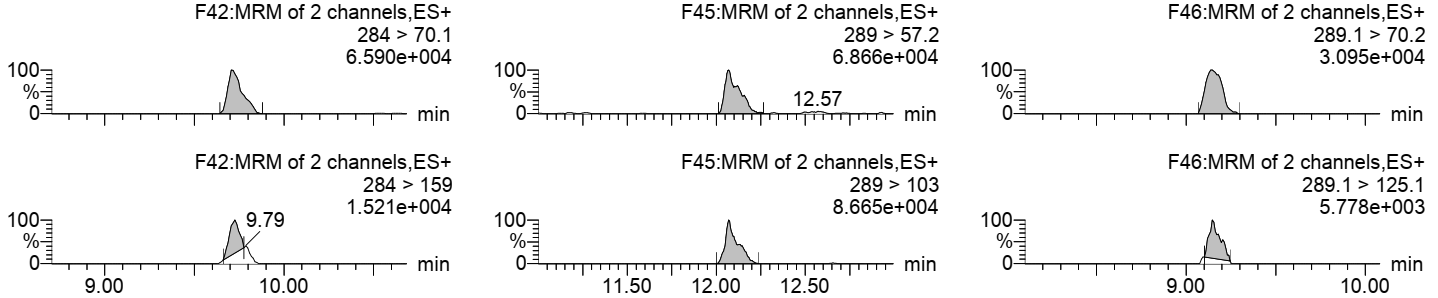

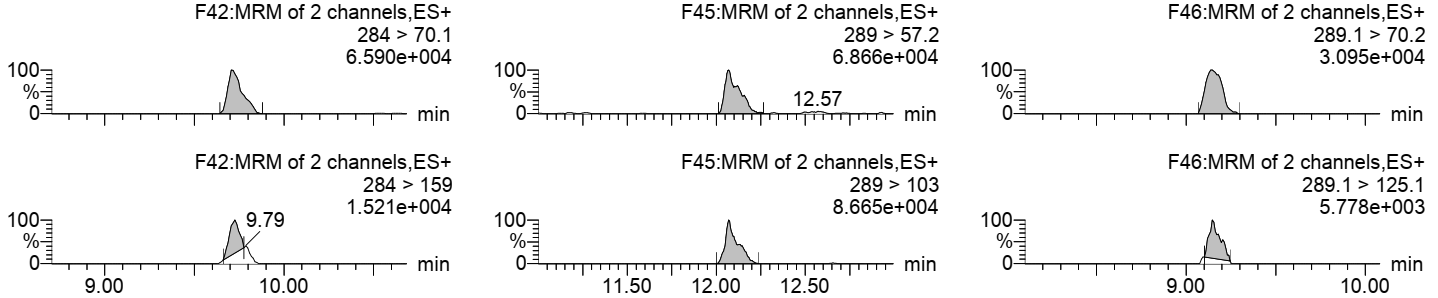


Penconazole (**37**) Terbufos (**38**) Myclobutanil (**39**)


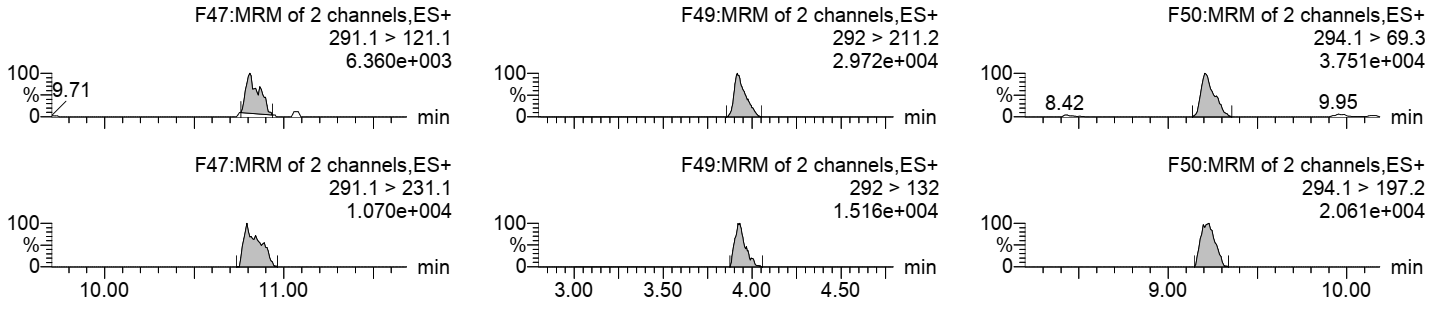

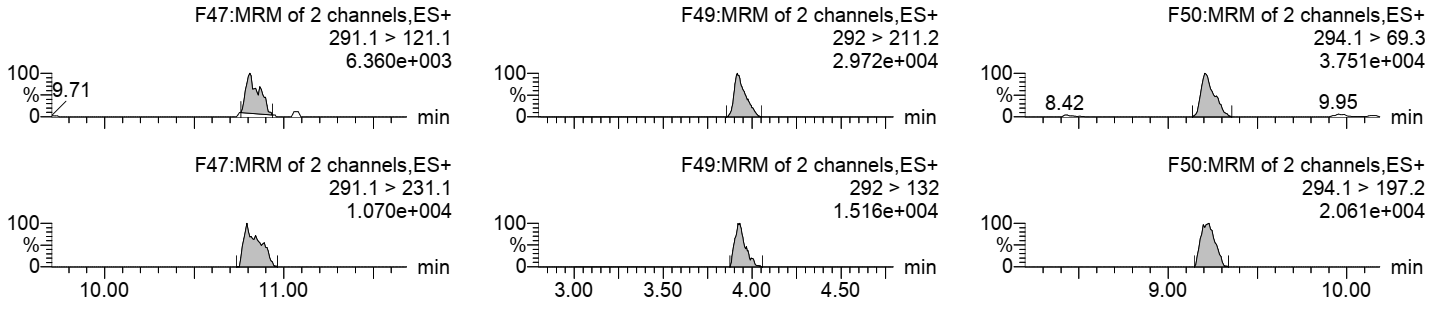

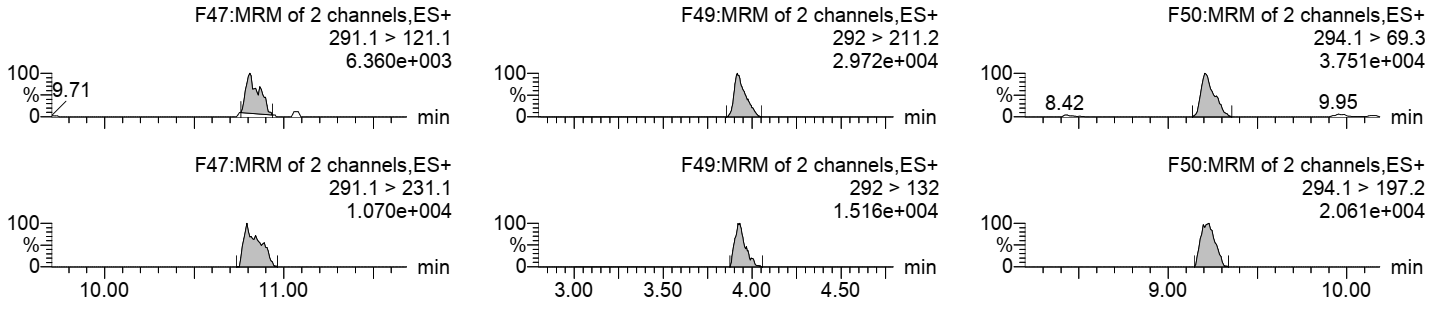


Isocarbofos (**40**) Thiamethoxam (**41**) Triadimefon (**42**)

**
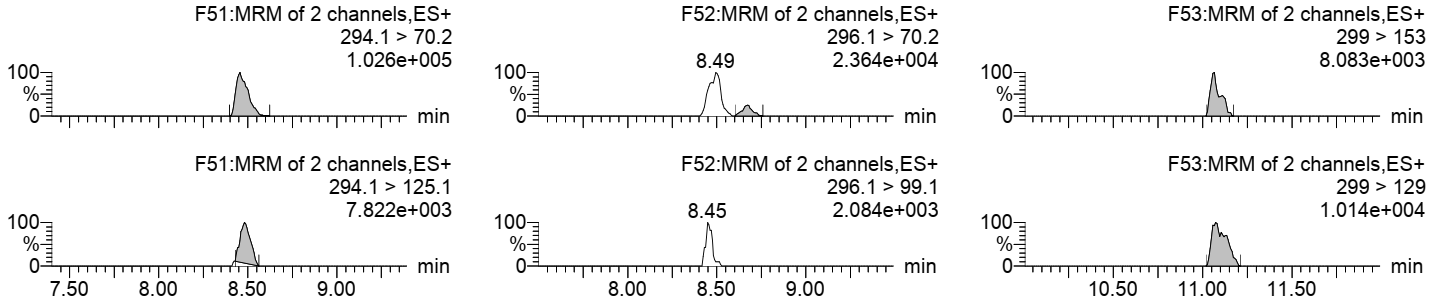

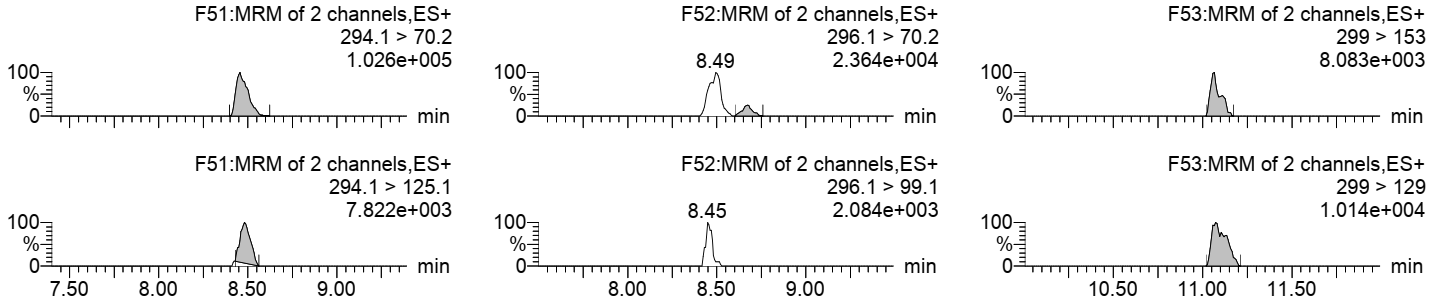
**
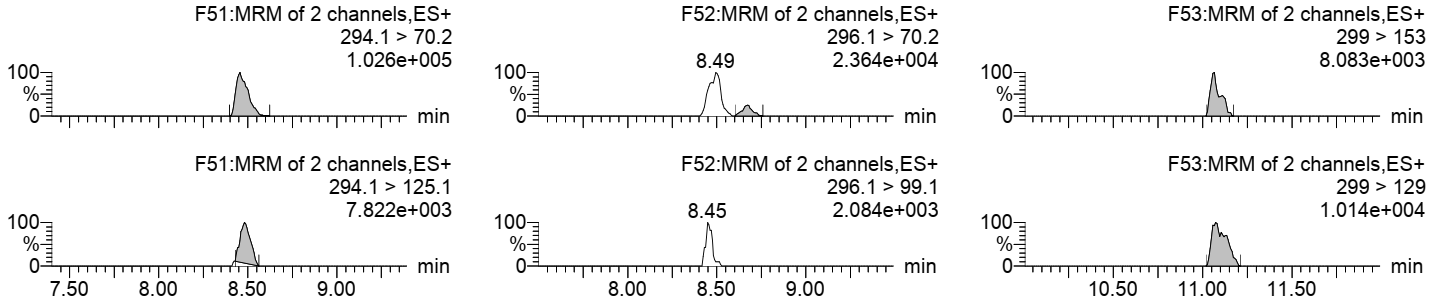


Paclobutrazol (**43**) Triadimenol (**44**) Phoxim (**45**)


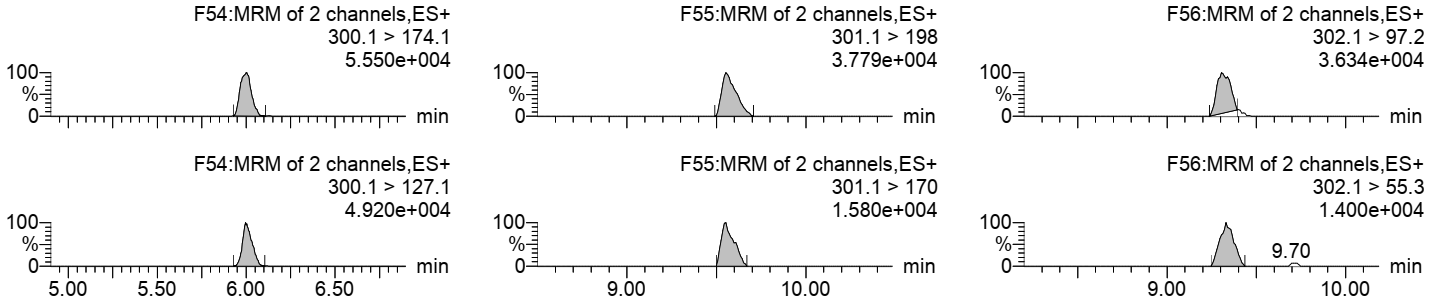

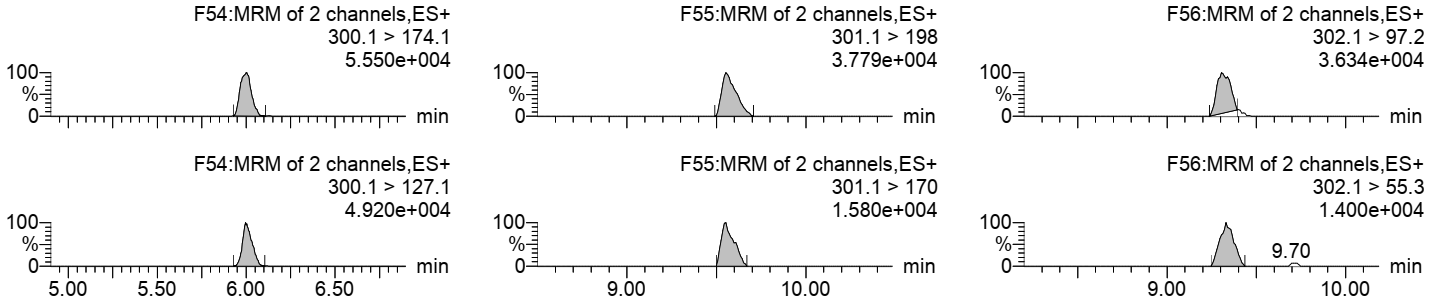

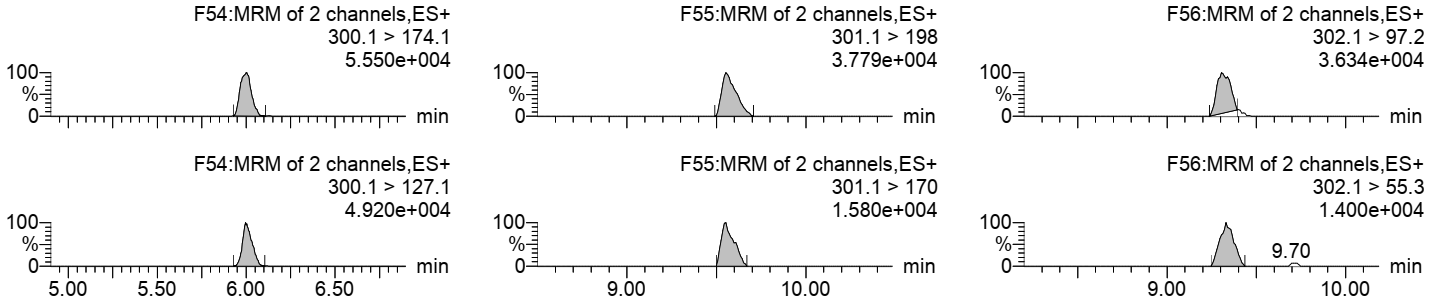


Phosphamidon (**46**) Bifenazate (**47**) Fenhexamid (**48**)


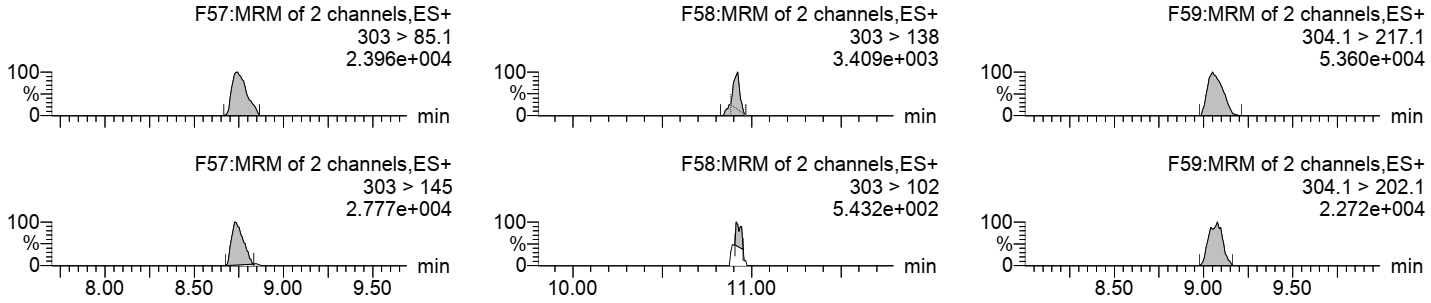

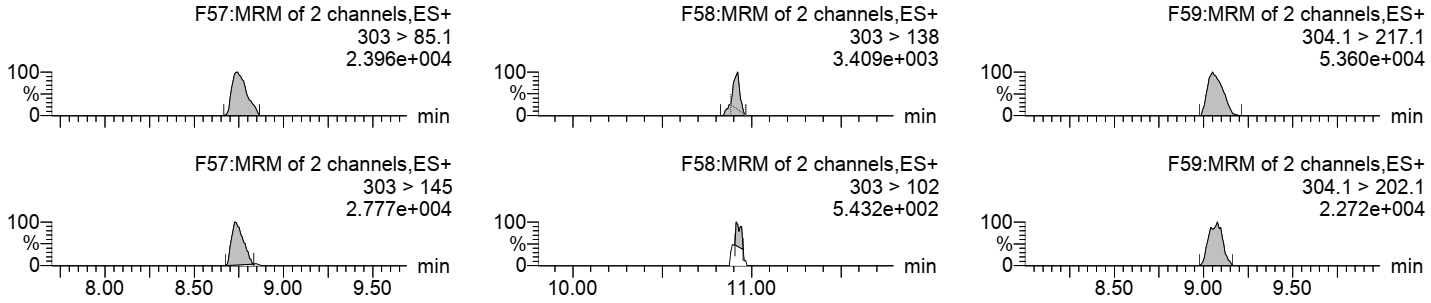

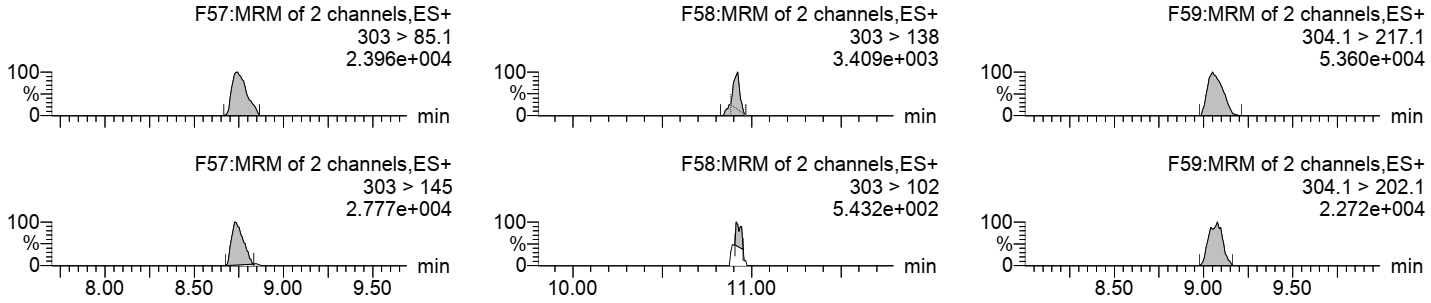


Methidathion (**49**) Clofentezine (**50**) Fenamiphos (**51**)


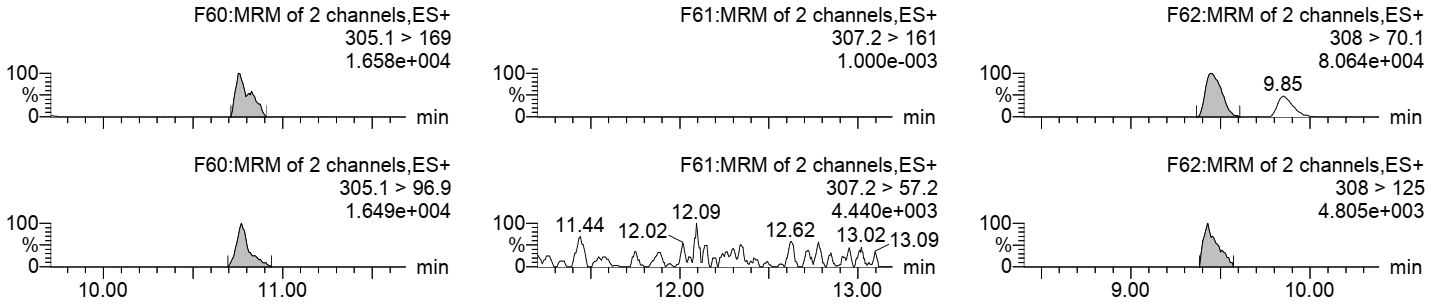

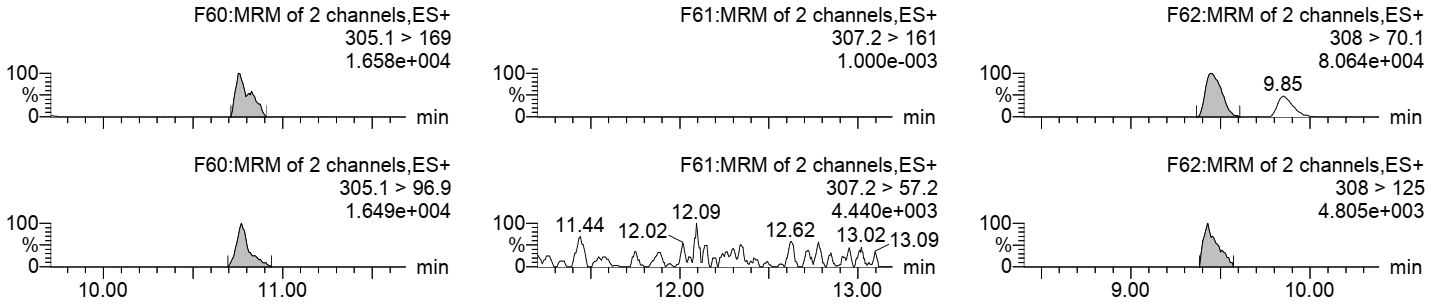

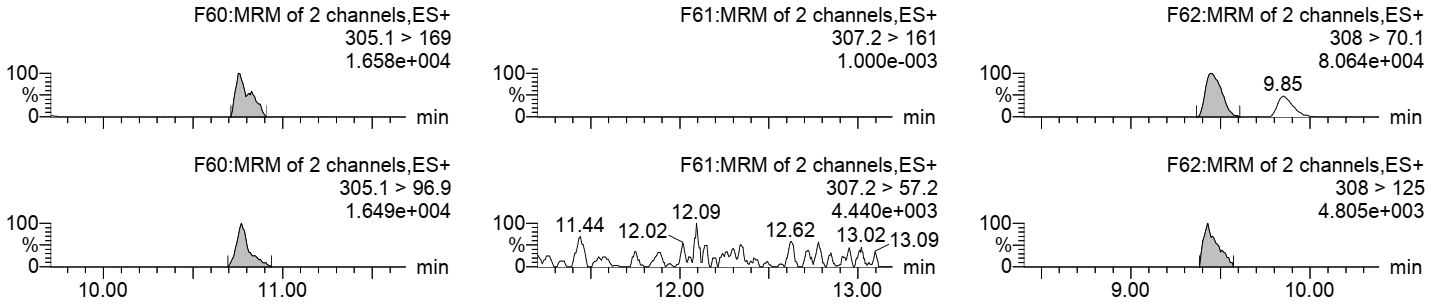


Diazinon (**52**) Fenazaquin (**53**) Tebuconazole (**54**)


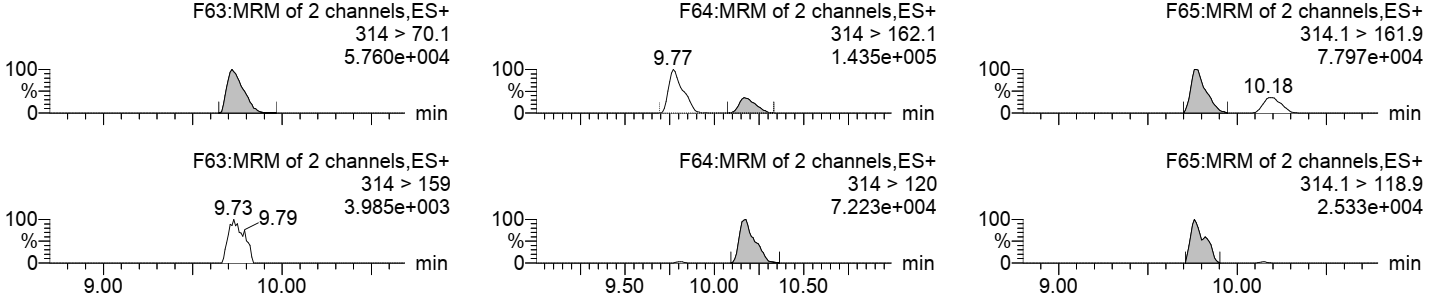

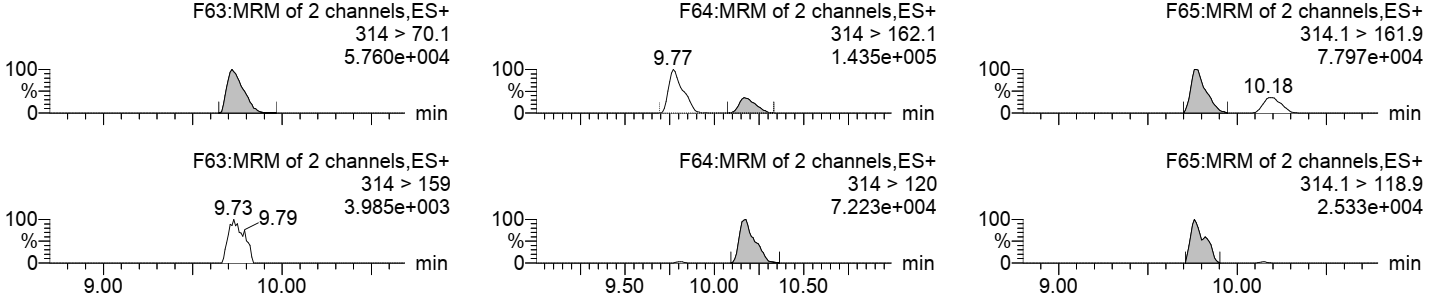

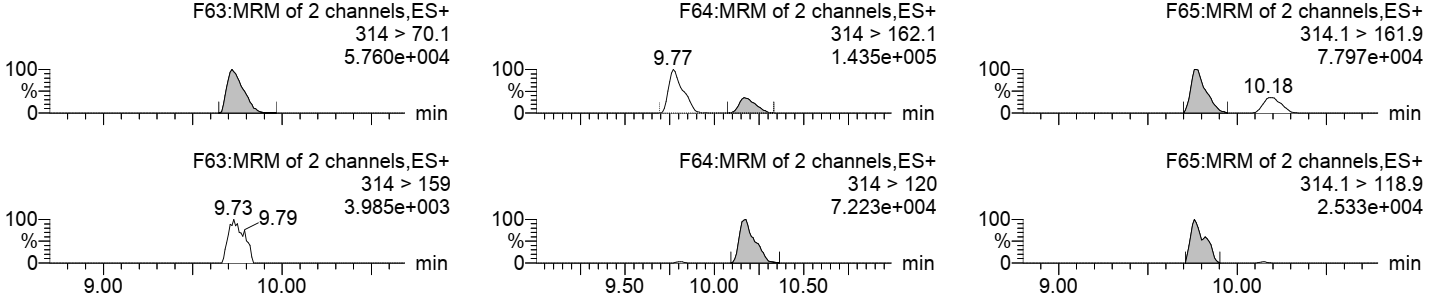


Hexaconazole (**55**) Isazophos (**56**) Triazophos (**57**)

**
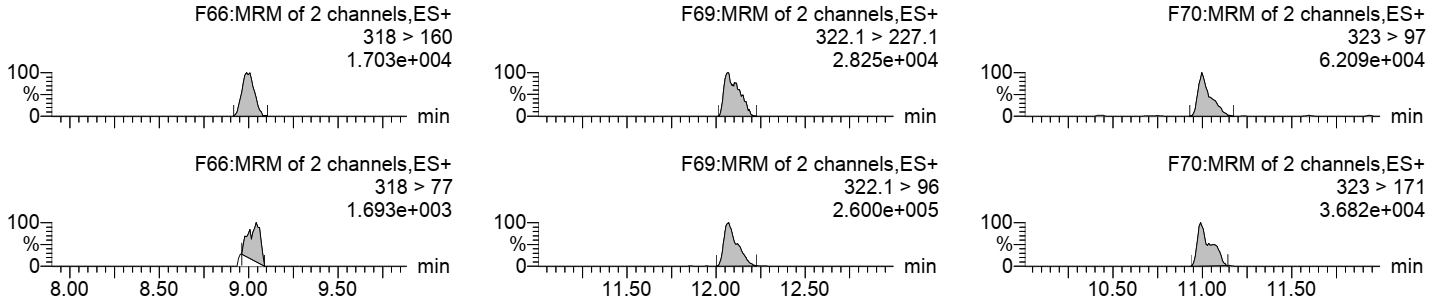

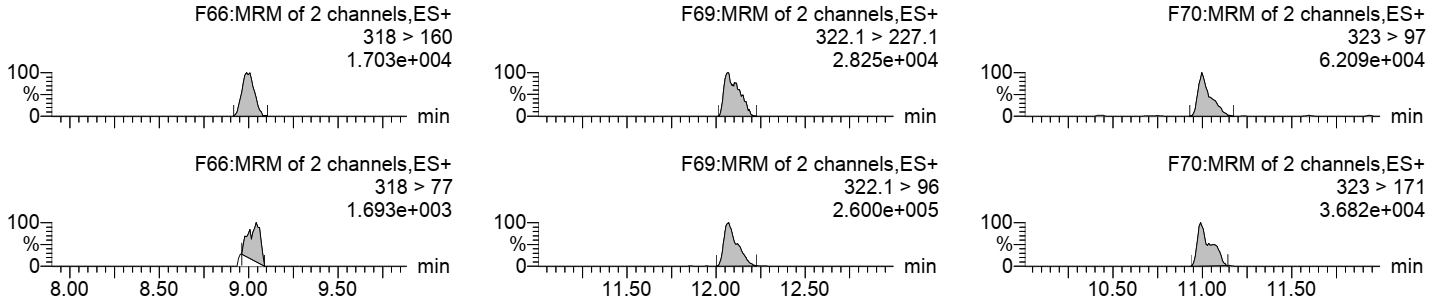

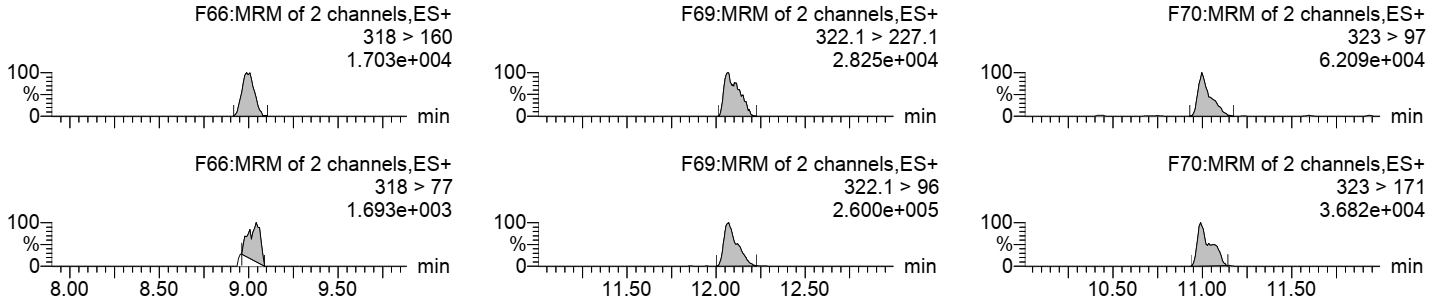
**

Phosmet (**58**) Pyriproxifen(**59**) Sulfotep (**60**)


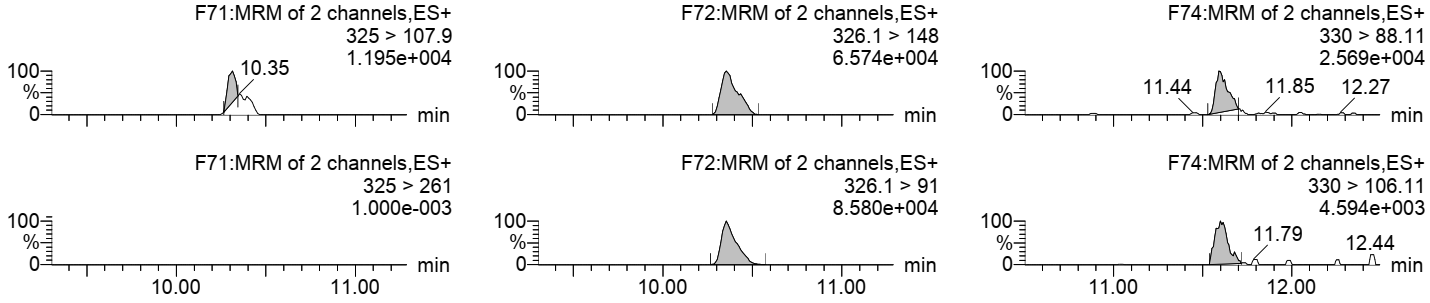

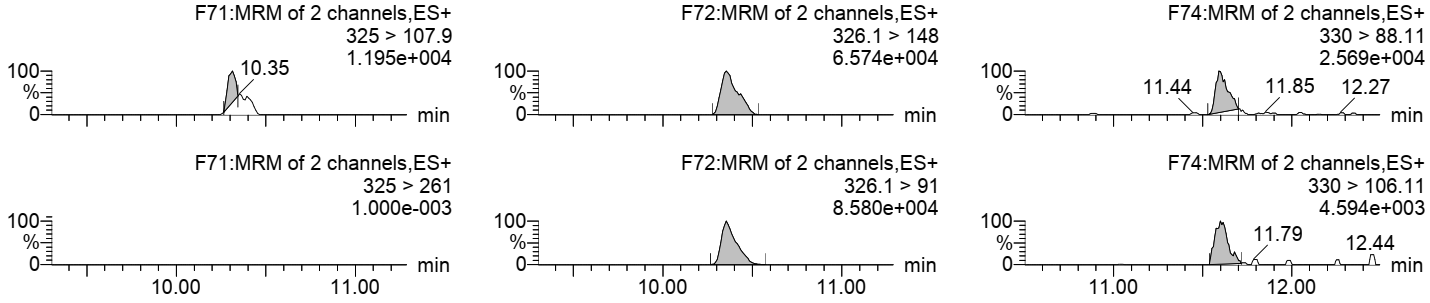

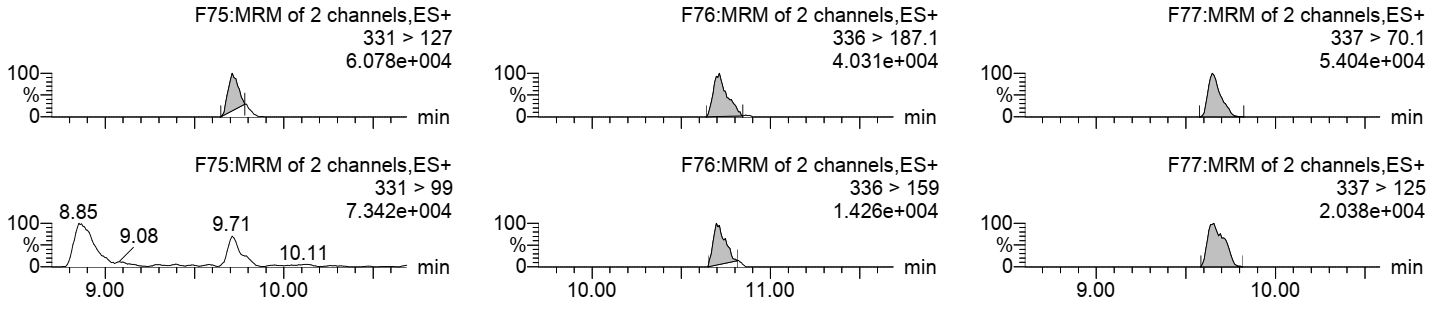


Cyazofamid (**61**) Benalaxyl (**62**) Malathion (**63**)


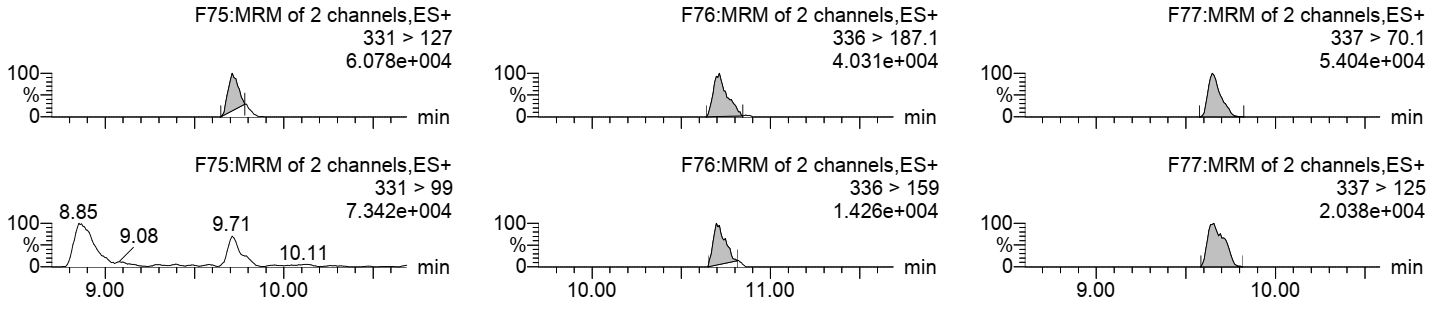

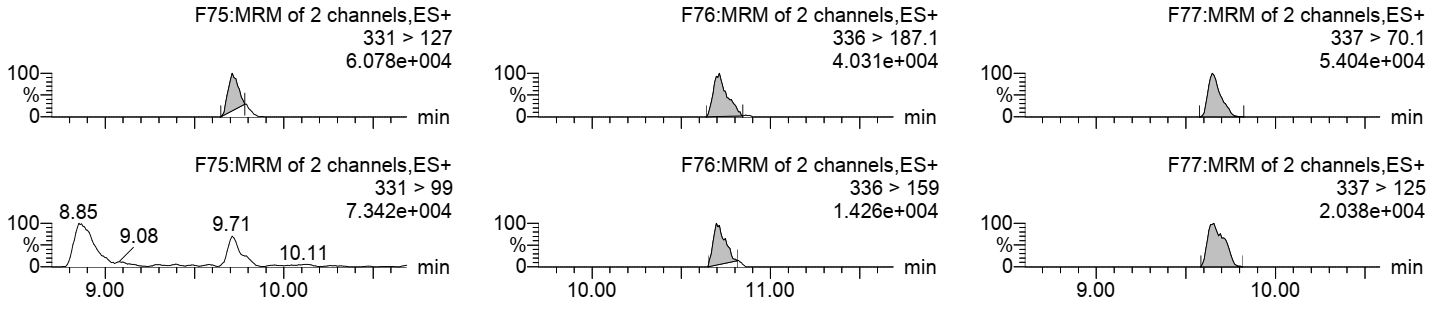

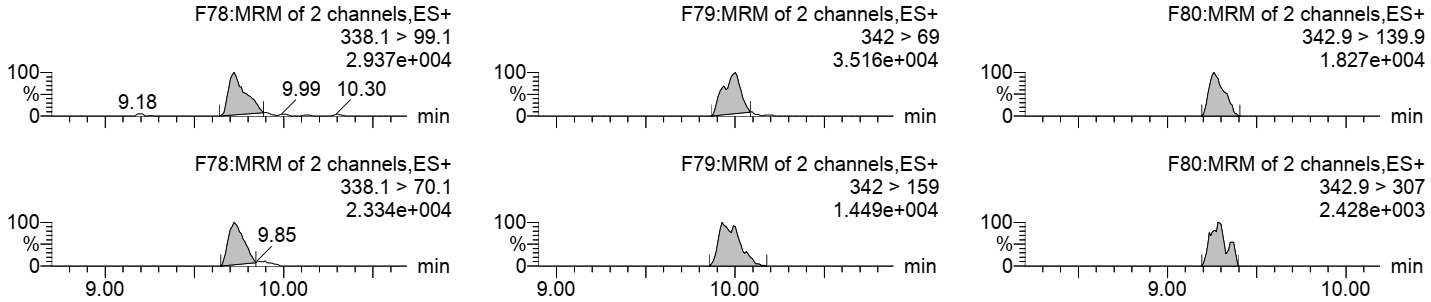


Zoxamide (**64**) Fenbuconazole (**65**) Biphentriazol (**66**)


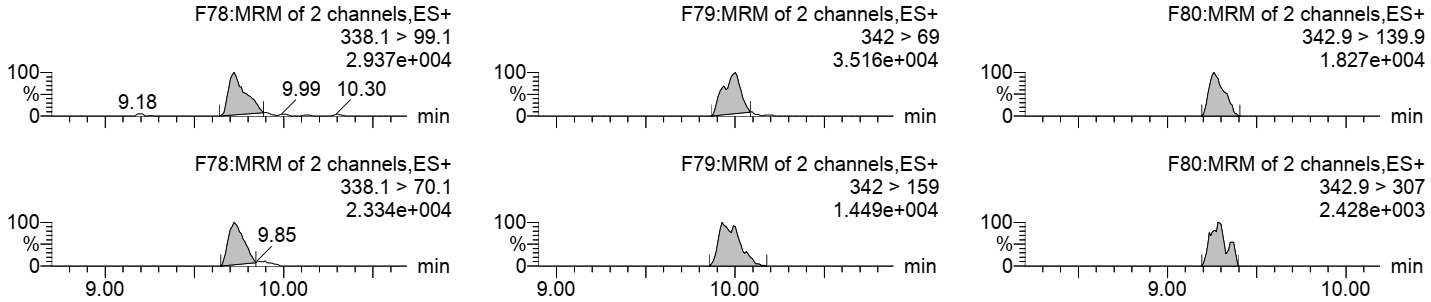

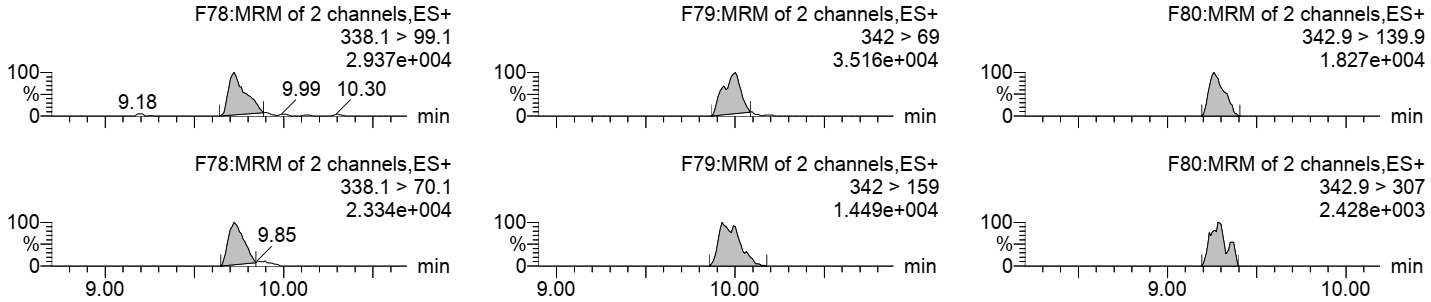

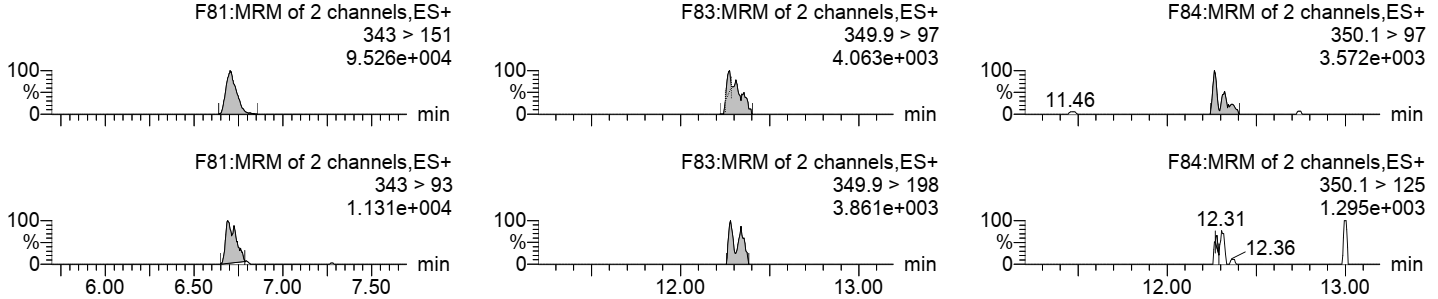


Propiconazole (**67**) Boscalid (**68**) Thiophanate (**69**)


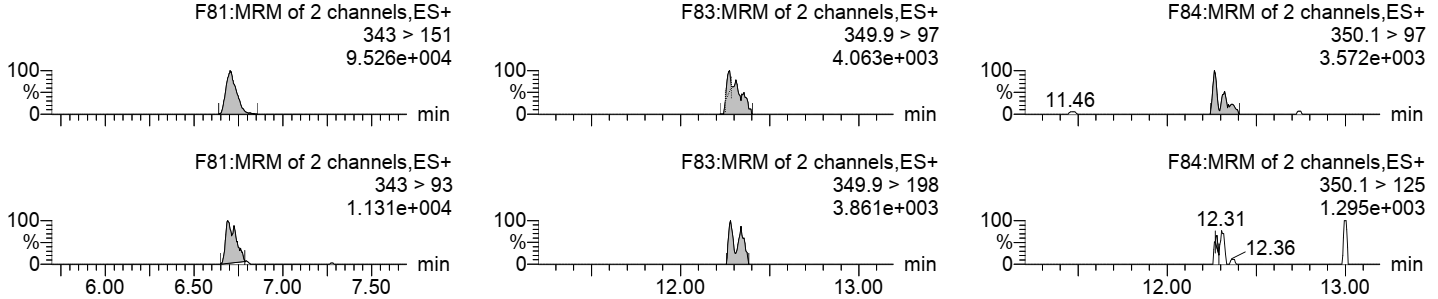

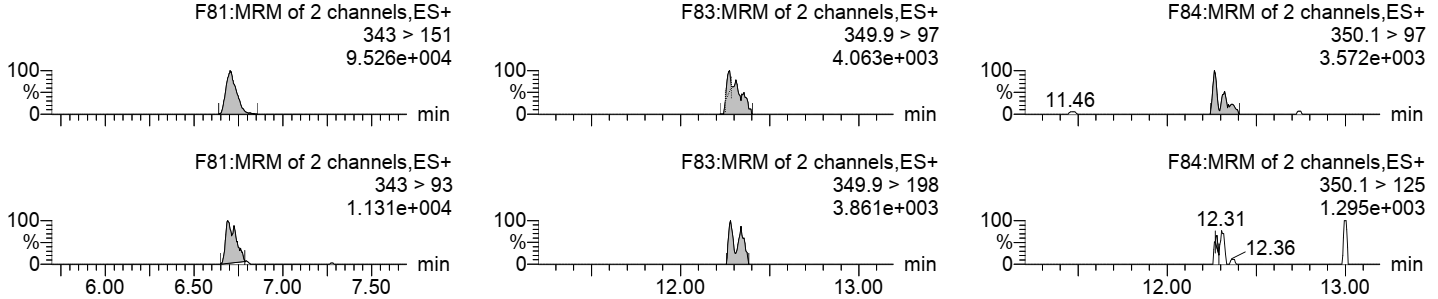
**
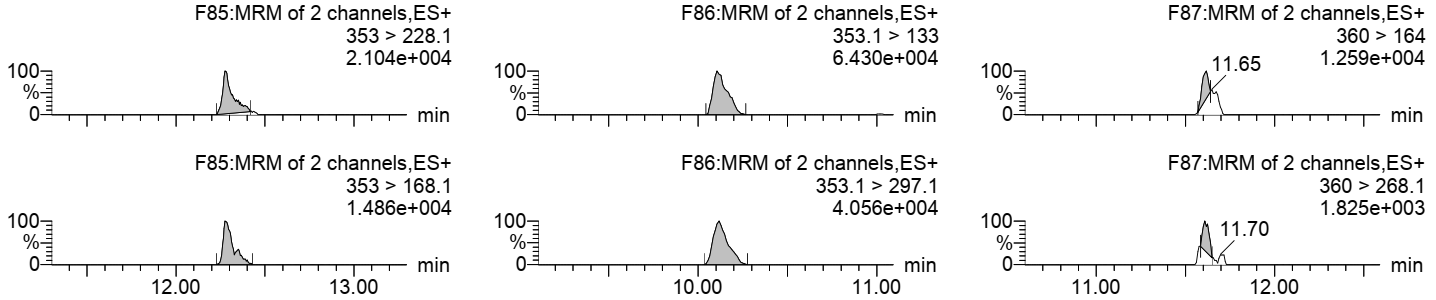
**

Chlorpyrifos (**70**) Fenpropathrin (**71**) Hexythiazox (**72**)

**
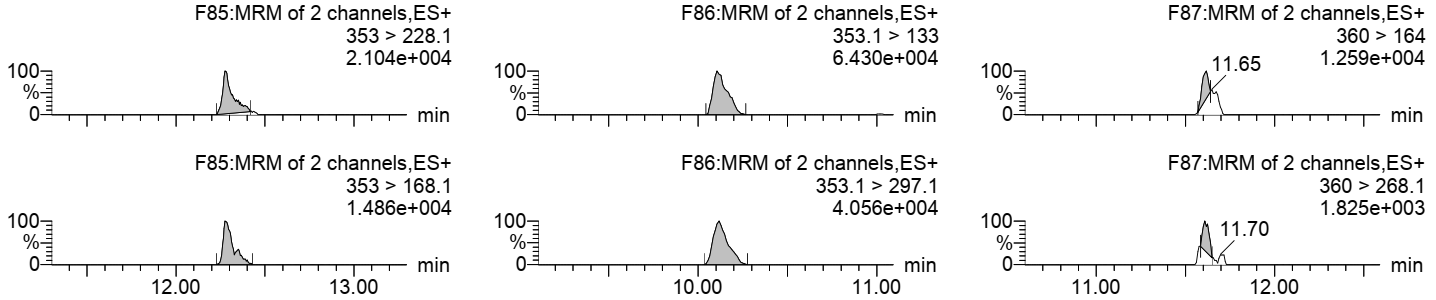

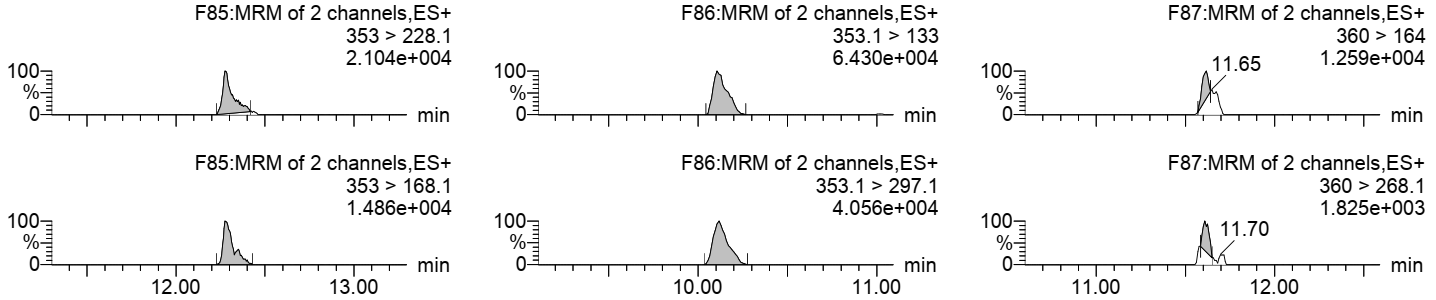
**
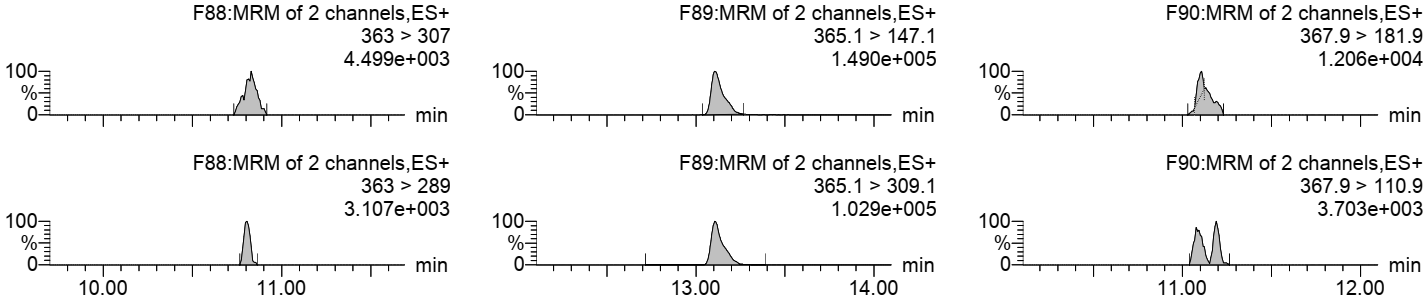


Tebufenozide (**73**) Clethodim (**74**) Coumaphos (**75**)


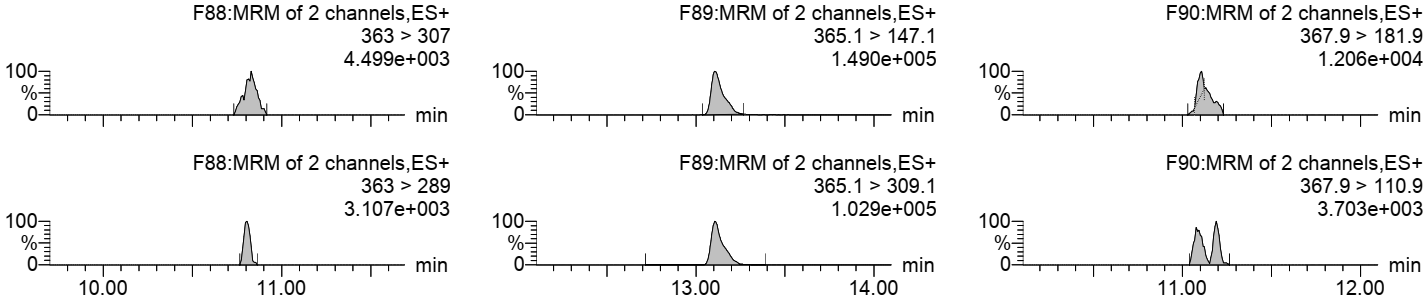

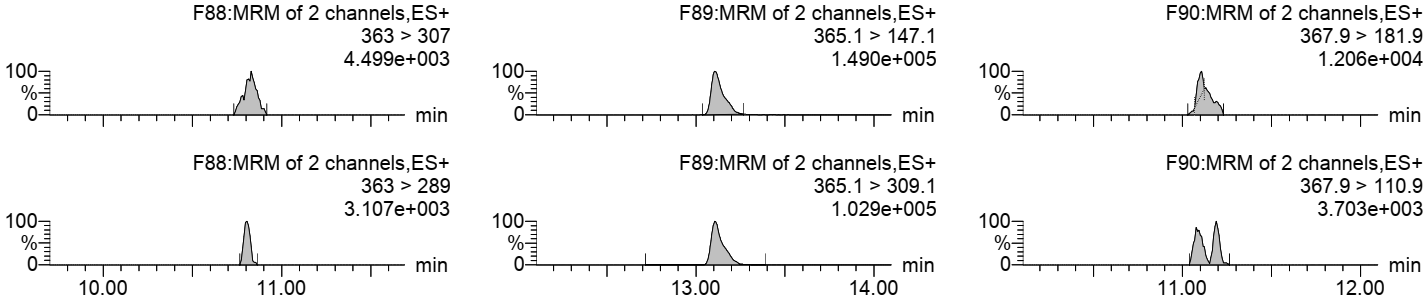

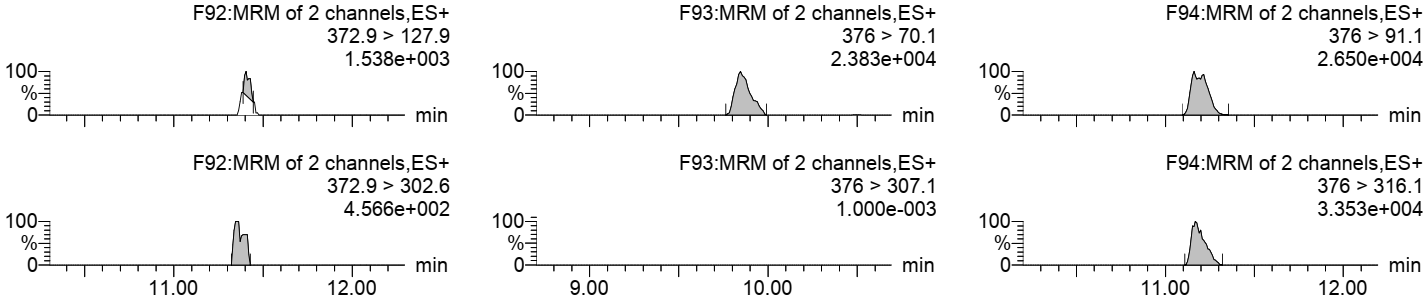


Pyridaben (**76**) Phosalone (**77**) Profenofos (**78**)


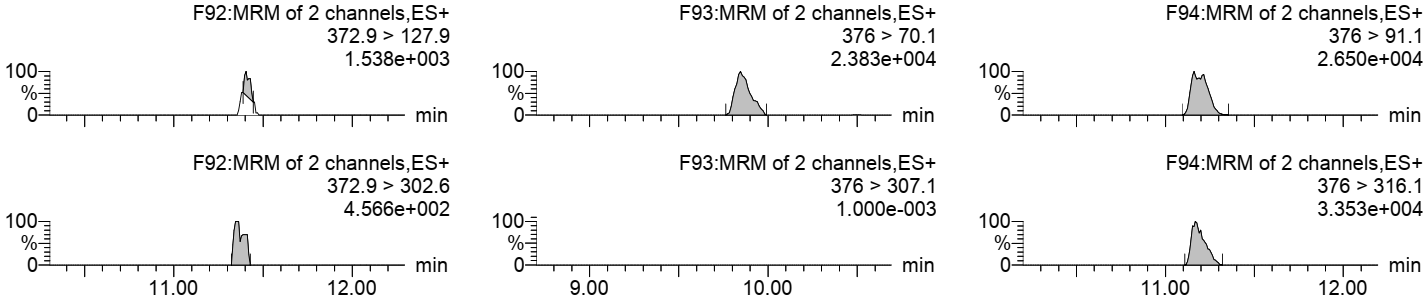

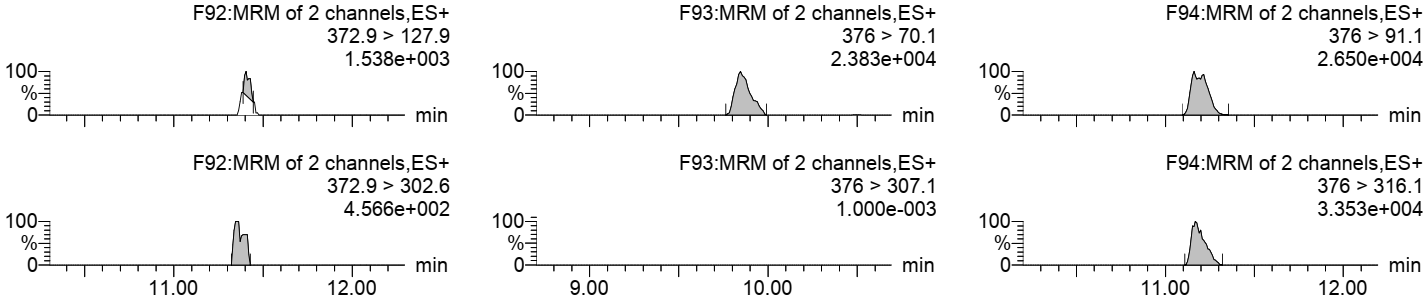

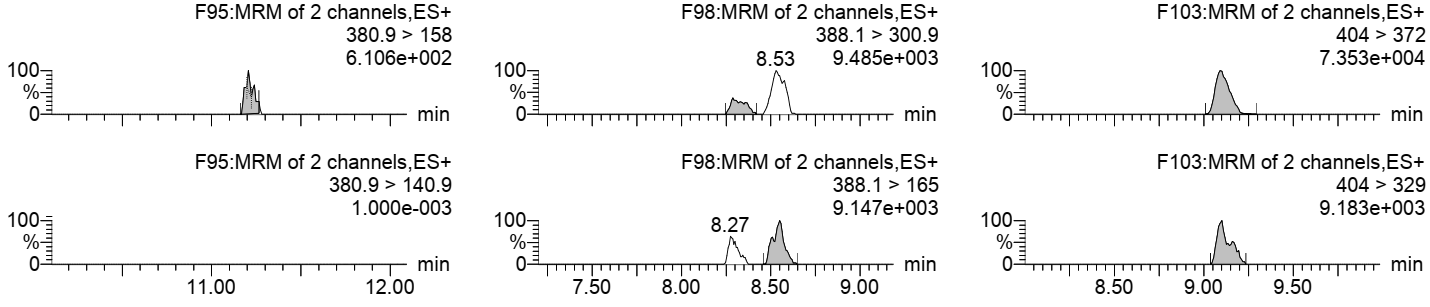


Pretilachlor (**79**) Haloxyfop (**80**) (**81**) Teflubenzuron


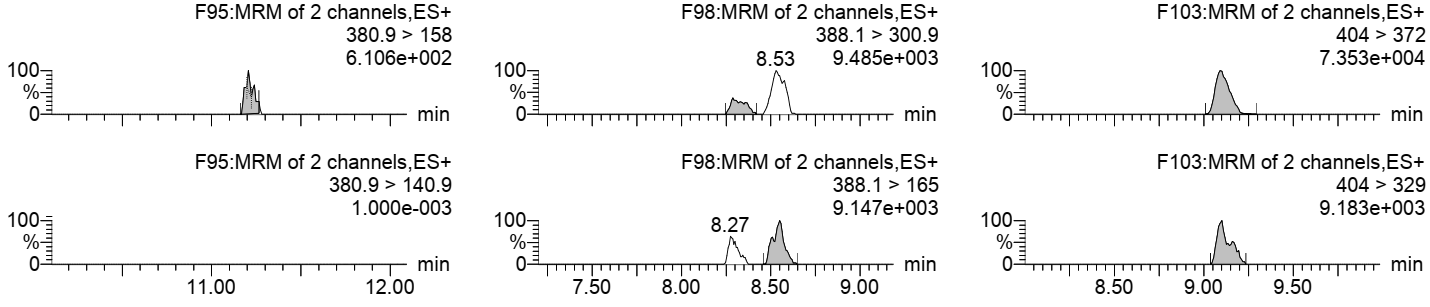

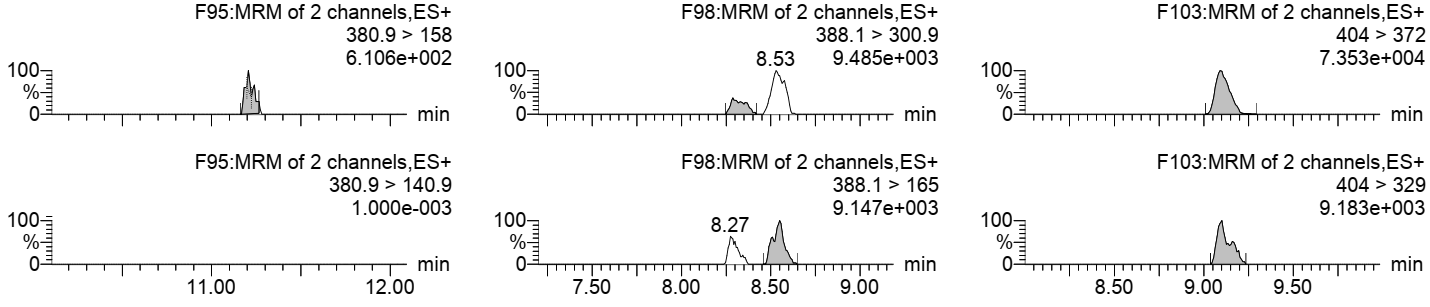

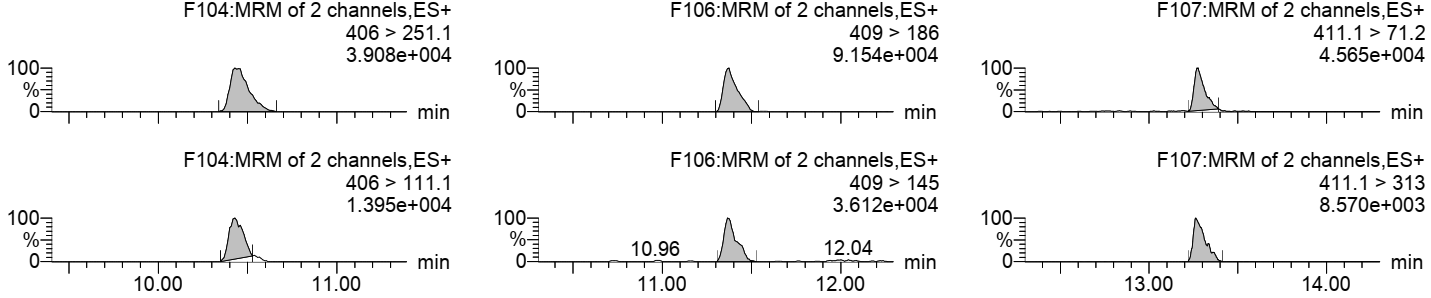


Dimethomorph( **82**) Azoxystrobin (**83**) (**84**) Difenoconazole


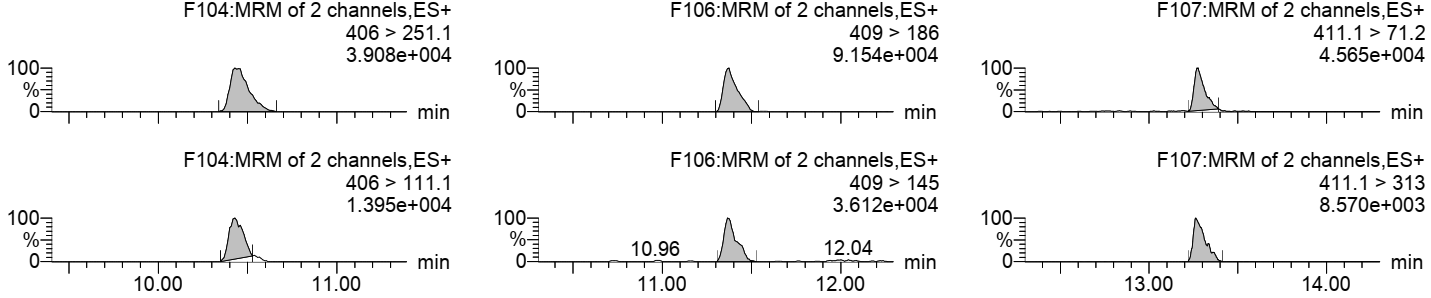

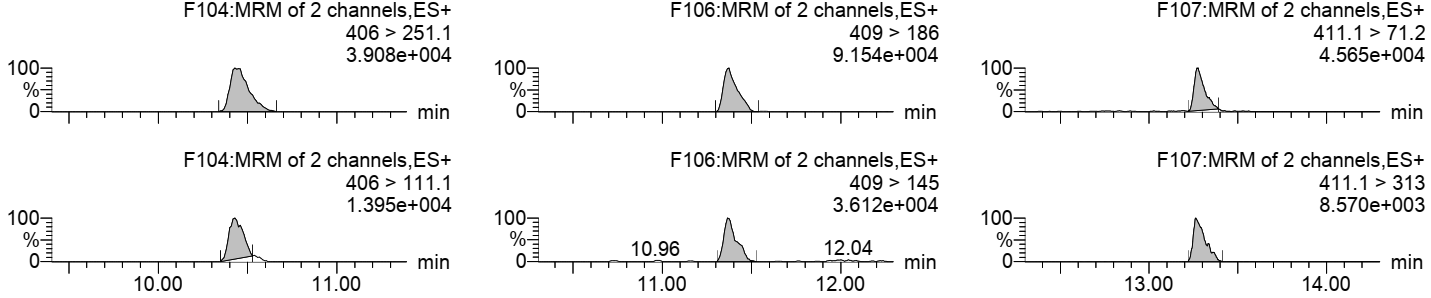
**
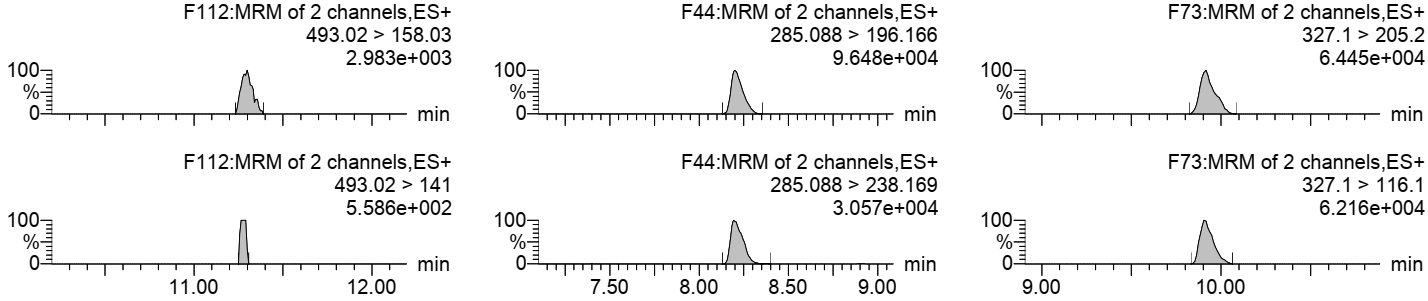
**

Trifloxystrobin (**85**) Spirodiclofen (**86**) Novaluron (**87**)


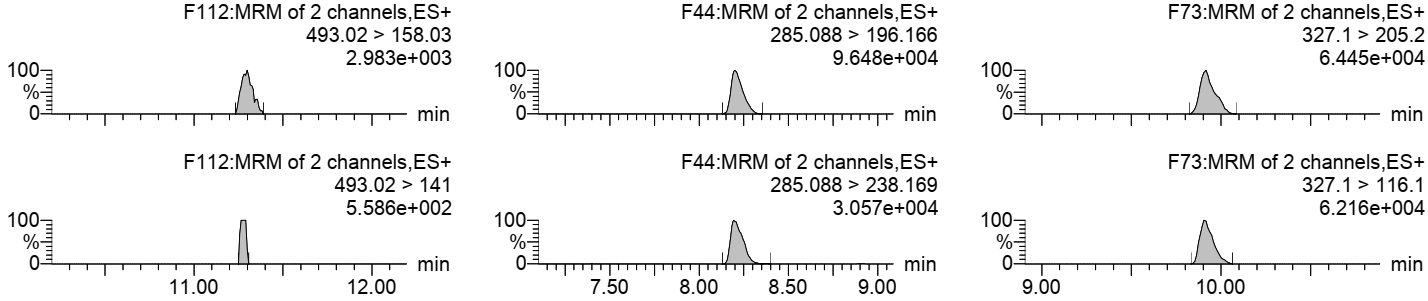
**
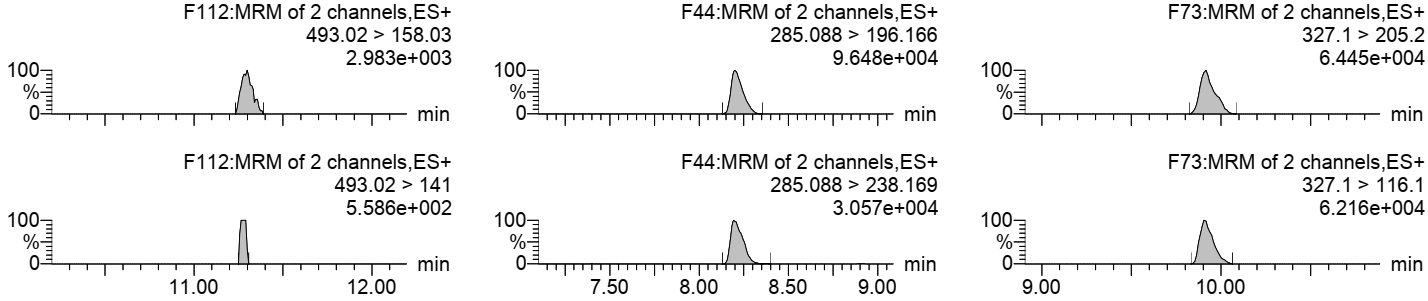
**
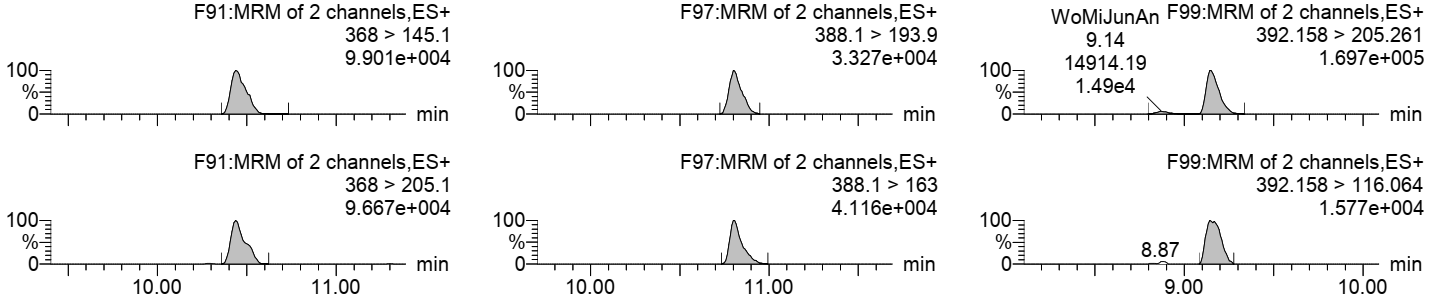


Metominostrobin (**88**) Dimoxystrobin (**89**) Boscalid (**90**)


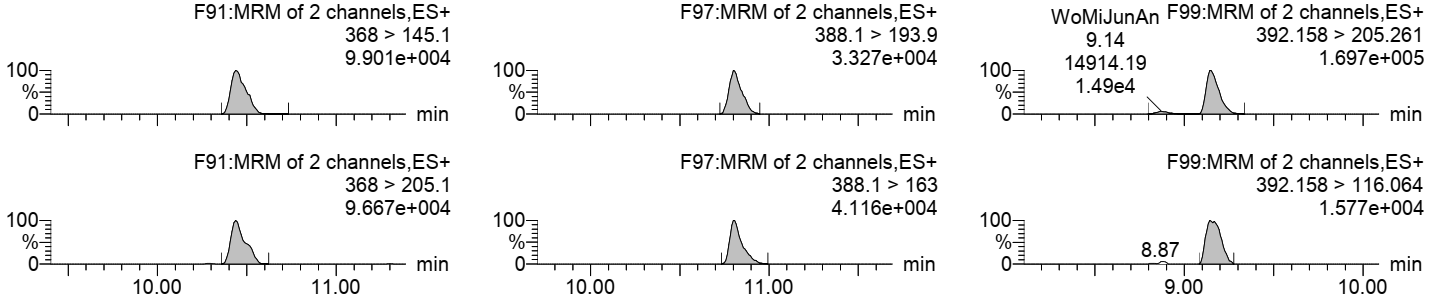

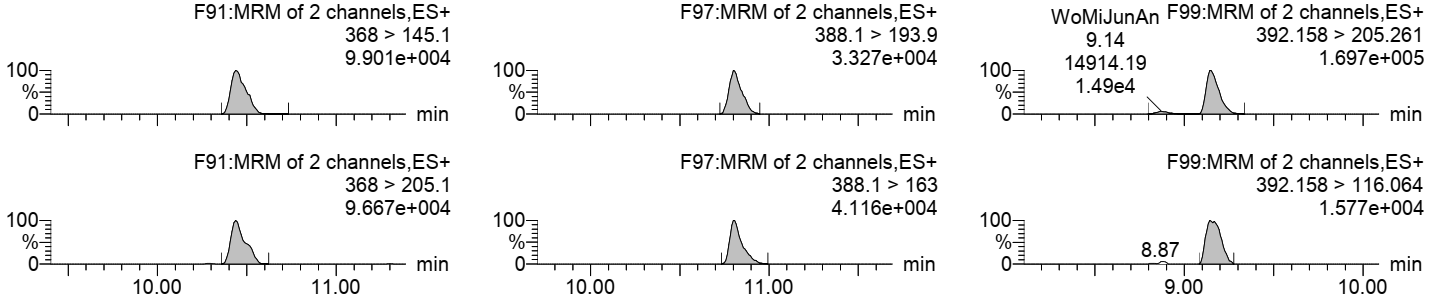

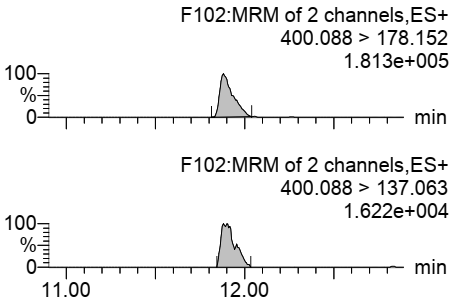


Pyraclostrobin (**91**) Orysastrobin (**92**) Enestroburin (**93**)


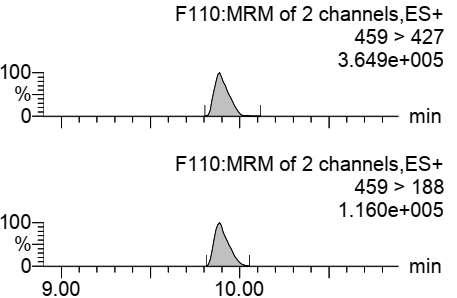


Fluoxastrobin (**94**)

**Figure. S3** The chromatograms of GC-MS/MS (A) andLC-MS/MS (B)
